# Disease effects on reproduction can cause population cycles in seasonal environments

**DOI:** 10.1111/j.1365-2656.2007.01328.x

**Published:** 2008-03

**Authors:** Matthew J Smith, Andrew White, Jonathan A Sherratt, Sandra Telfer, Michael Begon, Xavier Lambin

**Affiliations:** 1 Department of Mathematics and the Maxwell Institute for Mathematical Sciences, Heriot-Watt University Edinburgh EH14 4AS, UK; 2 Centre for Comparative Infectious Diseases, School of Biological Sciences, University of Liverpool Biosciences Building, Liverpool L69 7ZB, UK; 3 School of Biological Sciences, Zoology Building, University of Aberdeen Tillydrone Avenue, Aberdeen AB24 2TZ, UK

**Keywords:** cowpox virus, density dependence, disease model, population dynamics, seasonal forcing

## Abstract

1. Recent studies of rodent populations have demonstrated that certain parasites can cause juveniles to delay maturation until the next reproductive season. Furthermore, a variety of parasites may share the same host, and evidence is beginning to accumulate showing nonindependent effects of different infections.
2. We investigated the consequences for host population dynamics of a disease-induced period of no reproduction, and a chronic reduction in fecundity following recovery from infection (such as may be induced by secondary infections) using a modified SIR (susceptible, infected, recovered) model. We also included a seasonally varying birth rate as recent studies have demonstrated that seasonally varying parameters can have important effects on long-term host–parasite dynamics. We investigated the model predictions using parameters derived from five different cyclic rodent populations.
3. Delayed and reduced fecundity following recovery from infection have no effect on the ability of the disease to regulate the host population in the model as they have no effect on the basic reproductive rate. However, these factors can influence the long-term dynamics including whether or not they exhibit multiyear cycles.
4. The model predicts disease-induced multiyear cycles for a wide range of realistic parameter values. Host populations that recover relatively slowly following a disease-induced population crash are more likely to show multiyear cycles. Diseases for which the period of infection is brief, but full recovery of reproductive function is relatively slow, could generate large amplitude multiyear cycles of several years in length. Chronically reduced fecundity following recovery can also induce multiyear cycles, in support of previous theoretical studies.
5. When parameterized for cowpox virus in the cyclic field vole populations (*Microtus agrestis*) of Kielder Forest (northern England), the model predicts that the disease must chronically reduce host fecundity by more than 70%, following recovery from infection, for it to induce multiyear cycles. When the model predicts quasi-periodic multiyear cycles it also predicts that seroprevalence and the effective date of onset of the reproductive season are delayed density-dependent, two phenomena that have been recorded in the field.

## Introduction

Effects of disease on host population dynamics come about fundamentally via effects on host fecundity rates, survival rates, or both. Such effects have been demonstrated in a range of wild populations (summarized in Tompkins & Begon 1999; Tompkins *et al*. 2002) and a few studies have demonstrated the resulting impact on the long-term dynamics of their host populations (see Hudson, Dobson & Newborn 1998; Albon *et al*. 2002; Redpath *et al*. 2006 as examples). Further complexity may arise if disease effects do not operate independently from other factors that may also influence survivorship or fecundity, such as environmental variation (Jolles, Etienne & Olff 2006). It is also common for different parasites to coexist within the same host (reviewed in Cox 2001; Roberts *et al*. 2002; Pedersen & Fenton 2007). These could also potentially have interacting effects on host survival and fecundity. For example, epidemiological studies of humans have demonstrated that multiple infections can have important consequences for human health (Bradley & Jackson 2004; Druilhe, Tall & Sokhna 2005; Adams *et al*. 2006; Sachs & Hotez 2006). Recent studies have also shown that the multiyear dynamics of certain diseases may be influenced strongly by seasonal fluctuations in one or more biological mechanisms (reviewed recently by Altizer *et al*. 2006; see Stone *et al*. 2007 and He & Earn 2007 for recent examples). Understanding how changes in seasonality could alter host–parasite dynamics in wild populations is crucial if we are to predict the emergence of zoonoses or diseases that could threaten species survival (Daszak, Cunningham & Hyatt 2000; Harvell *et al*. 2002). Our work here builds upon previous theoretical studies that have demonstrated that, if diseases influence survival or fecundity rates strongly enough, then this can resonate with seasonal forcing to influence both host and parasite dynamics dramatically (Keeling, Rohani & Grenfell 2001; Keeling & Grenfell 2002; Dushoff *et al*. 2004; Greenman, Kamo & Boots 2004; Hosseini, Dhondt & Dobson 2004; Ireland, Norman & Greenman 2004; Koelle, Pascual & Yunus 2005; He & Earn 2007; Ireland, Mestel & Norman 2007). Here we investigate mathematically how the dynamics of seasonally reproducing host populations may be affected by diseases that induce a period of no reproduction, or chronically reduce fecundity, following recovery from infection. Previous theoretical studies have already illustrated that both macro and microparasitic infection-induced reductions in host fecundity can destabilize the dynamics of their host populations sufficiently to induce multiyear cycles (Anderson & May 1981; Dobson & Hudson 1992; White, Bowers & Begon 1996; Boots & Norman 2000). However, to our knowledge, the implications of an infection-induced period of no reproduction have not yet been investigated. How the various disease effects may interact with seasonal forcing is also poorly understood for wildlife populations. We construct a model with cyclic microtine populations in mind primarily because evidence for disease effects on fecundity in these is particularly strong. Our choice is also convenient because data on population growth rates, survivorship rates and seasonality in reproduction are available for a variety of populations and species, and the long-term host dynamics for several different populations are known. Moreover, the impacts of disease on host fecundity are known in some cases, allowing us to investigate the model predictions for certain diseases.

A wide variety of pathogens have been detected in microtine rodent populations (Findlay & Middleton 1934; Chitty 1954; Soveri *et al*. 2000; Noyes *et al*. 2002; Olsson *et al*. 2002; Telfer *et al*. 2002, 2007; Bown *et al*. 2003; Fichet-Calvet *et al*. 2003, 2004; Bown, Bennett & Begon 2004; Cavanagh *et al*. 2004; Telfer *et al*. 2005; Bown *et al*. 2006; Burthe *et al*. 2006; Niklasson *et al*. 2006; Smith *et al*. 2006a; Stenseth *et al*. 2006). The effects of most of these are unknown or are only beginning to be understood. However, increased mortality rates as a result of infection from various diseases have been known for a long time and may play a role in the dynamics of rodent population crashes (Findlay & Middleton 1934; Elton, Davis & Findlay 1935; Soveri *et al*. 2000; Singleton *et al*. 2005). Recent studies of cowpox virus infection have demonstrated its effects on both host survivorship and reproductive timing. In the case of wild populations of bank voles (*Clethrionomys glareolus* Schreber) and wood mice (*Apodemus sylvaticus* L.) in UK deciduous woodland (Manor Wood), Telfer *et al*. (2005) demonstrated that juvenile females infected with cowpox were likely to delay reproduction until the following breeding season. Such phenotypic plasticity in age to first breeding is predicted to occur in species in which reproductive maturity has significant survival costs, and is thought to be a general characteristic of rodent populations (Lambin & Yoccoz 2001; Telfer *et al*. 2005). Depending on the time of year analysed, cowpox infection was also shown to increase or decrease survival in the Manor Wood populations. Contrastingly, infection by the same pathogen resulted in dramatic reductions in survival probabilities in cyclic field vole (*Microtus agrestis* L.) populations in UK grassland habitat (Kielder Forest) (Burthe *et al*. in press). However, the significance of these effects for long-term population dynamics has not been investigated before now.

Long-term studies in Kielder Forest have shown that the prevalence of both vole tuberculosis infection and cowpox antibodies (i.e. cowpox seroprevalence: denoting the proportion of animals with recent or past infections) are related significantly to past population densities, with similar lags (Cavanagh *et al*. 2004). Moreover, the probability of a vole initially becoming infected with cowpox also shows a similar lag (Burthe *et al*. 2006). Recently, we showed that if the timing of the onset of seasonal reproduction was influenced by past population densities, as found in Kielder Forest field voles, then this could result in multiyear population cycles (Smith *et al*. 2006b). However, it is as yet unknown whether microparasitic infections, through their effects on vole reproductive timing, could cause the onset of the reproductive season to be delayed density-dependent.

In this study, we first address whether microparasitic diseases could regulate or induce multiyear cycles in seasonally reproducing populations by influencing reproductive timing and reproductive output. We then ask how widespread disease-induced multiyear cycles could be in rodent populations living in seasonal environments. To address this question we explore the model dynamics for parameters representative of a range of rodent populations and a wide variety of microparasitic diseases. Lastly, we investigate the model predictions when parameterized for Kielder Forest field vole populations, as data on the prevalence of cowpox virus are available for this system. In addition, there is mounting evidence that a guild of potentially interacting parasites infect cyclic field voles in Kielder Forest (Birtles *et al*. 2001; Noyes *et al*. 2002; Bown *et al*. 2003, 2004, 2006; Cavanagh *et al*. 2004; Smith *et al*. 2006a; Telfer *et al*. 2007). Lastly, for the Kielder Forest field vole populations, we investigate whether the empirically observed phenomena of delayed density-dependent season length and seroprevalence are predicted as epiphenomena of disease-induced multiyear cycles.

## Methods

### parameter definitions and model structure

The model we use is a modification of the classical host–parasite framework (Anderson & May 1981) to incorporate no reproduction by the infected class, an immune class that also cannot reproduce, and seasonal reproduction. The host population density is divided into four classes of individual: those that are susceptible to microparasitic infection, *S*, infected individuals that cannot reproduce,*I*, recovered and immune individuals that cannot yet reproduce, *Y* and recovered and immune individuals that can reproduce, *Z*, but potentially at a lower maximum reproductive rate than for those in the *S* class. A seasonal component to the model is included through a time-dependent birth rate. The change in the densities of the host classes over continuous time, *t*, is modelled with the four ordinary differential equations
eqn 1a
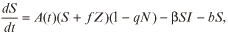

eqn 1b
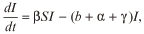

eqn 1c
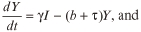

eqn 1d
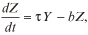

where:

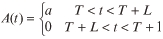


Here *L* is the reproductive season length in units of a fraction of 1 year, *T* is time in integer years and *N* = *S* + *I* + *Y* + *Z* is the total population density. We assume that disease-free per capita death rate, *b*, is constant throughout the year but that the per capita birth rate is seasonal [*A*(*t*)], with no births possible in the non-reproductive season (*A* = 0) and a constant maximum per capita birth rate in the reproductive season (*A = a*). Using other birth rate functions, such as a sinusoidal function (Barlow & Kean 1998; Greenman *et al*. 2004; Ireland *et al*. 2004; He *et al*. 2007), has only a minor quantitative effect on our results (not shown); *a* must be greater than *b* for voles (and the disease) to persist in the model; *a* > *b* is therefore assumed throughout this study. The birth rate is assumed to be density dependent and is modified due to a susceptibility to crowding coefficient, *q*, which is related the carrying capacity *K* = (*a – b*)/*aq*.

We assume density-dependent transmission at rate β, although assuming frequency dependent infection rates (β*SI*/*N*) does not affect our findings qualitatively (not shown). Infected individuals potentially have an increased mortality rate due to effects of the disease (α), and recover at a constant rate γ. Recovered individuals initially enter an immune but non-reproductive class which they leave at rate τ and regain a proportion of their reproductive capacity *f*(0 < *f* < 1). The total average reproductive delay following infection is therefore (1/γ) + (1/τ) years.

### parameterization

The model parameters can be divided into two groups: those that are specific to the rodent populations and are independent of the disease, and those that describe the effects of the disease on the rodent populations. We chose five combinations of the rodent specific parameters from published data sets to represent estimates from a variety of rodent populations (Table 1; see Supplementary material, Appendix S1 for their derivation). Differences between our estimates of the rodent population parameter values (e.g. the maximum per capita growth rate, *r* = *a – b*) reflect, at least partly, our uncertainly in the true parameter values, although true differences are expected between the different species considered. We examined a broad range of disease parameter values for each set of rodent parameters (Table 1). The two recovery rates, γ and τ, were varied to give an average recovery time from 2 years (γ or τ = 0·5 year^−1^: an extremely low value given that very few rodents would live this long) to 1 week (γ or τ = 52 year^−1^). The transmission rate, β, was varied between high (β = 0·9 ha year^−1^) and low (β = 0·05 ha year^−1^) infectivity. Disease-induced mortality was varied from a 50% mortality per month (α= 8·4 year^−1^) to no mortality (α = 0). We explored the full range of reduced fecundity following infection, *f*, from *f* = 0 to *f* = 1.

**Table 1:** List of parameters, their definitions, units and values used in this study. Site and species codes are Ki = field voles in UK grassland (*Microtus agrestis* in Kielder Forest), MW = bank voles in UK mixed woodland (*Clethrionomys glareolus* in Manor Wood), Fs = field voles in Fennoscandian grassland (*M. agrestis* in northern Finland), Ho = grey-sided voles in Japanese natural woodland (*C. rufocanus* Sundevall in Hokkaido), Fra = French common vole in agricultural habitat (*M. arvalis* Pallas in south-western France). Sources for parameter values are detailed in Supplementary material, Appendix S1. The model dynamics were systematically investigated for all possible combinations of the parameter values in brackets for each set of site-specific parameters

			Parameter values for each site				
			
Symbol	Definition	Units	Ki	MW	Fs	Ho	Fra
*K*	Maximum population density. Used to calculate *q*	Voles ha^−1^	250	150	100	80	2000
*r*	Maximum per capita growth rate. Used to calculate *a*	Year^−1^	2·5	1·8	6	5	4
*b*	Per capita death rate	Year^−1^	3·7	3·1	3·5	3·1	3·1
*L*	Fraction of year that is reproductive season	Years	7/12	7/12	6/12	6/12	8/12
*a*	Maximum per capita birth rate in the reproductive season	Year^−1^	*a* = (*r* + *b*)/*L*				
*q*	Density dependence coefficient	1/(voles ha^−1^)	*q* = (*a* – *b*)/*aK*				
α	Disease-induced mortality rate	Year^−1^	0–8·4, (0, 2·1, 4·2, 6·3, 8·4)				
β	Infection rate	ha year^−1^	0·05–1, (0·05, 0·1, 0·2, 0·5, 0·9)				
1/γ	Mean recovery time	Years	2 years –1 week (2, 1, 0.5, 1/12, 1/52)				
1/τ	Mean time to recover reproductive function following recovery from infection	Years	2 years –1 week (2, 1, 0.5, 1/12, 1/52)				
*f*	Reduced birth rate following recovery	Proportion	0–1, (0, 0·25, 0·5, 0·75, 1)				

### infection threshold

It is straightforward to show for equation 1 that the threshold density of susceptible individuals required for the infected population density to increase is:
eqn 2
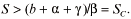


We use *S*_C_ to visualize when the rate of change of the infected population is positive or negative in population time–series plots. Another common way of expressing this threshold it to formulate it as the basic reproductive rate *R* = β*S*/(*b* + α + γ), which is the expected number of secondary infections produced on average per infected individual (Anderson *et al*. 1981). This has the simple interpretation that *I* can only increase if *R* > 1. Therefore, *R* = 1 at *S* = *S*_C_.

### mathematical analysis of the non-seasonal models

In this section we summarize briefly the dynamics predicted by the separate reproductive and non-reproductive season components of equation 1. The importance of seasonally forced birth rates in determining the multiyear dynamics can then be assessed. Appendix S2 gives details of the analyses performed in this section.

It is straightforward to show that when the disease is absent, the susceptible population density declines monotonically to zero when *A* = 0 (non-reproductive season equations) and increases monotonically to *S* = *K* when *A* = *a* (reproductive season equations). It is also straightforward to show that the long-term dynamics predicted by equation 1, when *A* = 0 and disease is present, is for the exponential decay of all component population densities to zero. For the reproductive season equations (*A* = *a*), the disease can become endemic in the population if *K* > *S*_C_. In this case, for most values of the other parameters, an equilibrium is approached through a series of damped oscillations to *S* = *S*_C_. The parameters τ and *f* have no effect on inequality (equation 2), and therefore will ultimately have no effect on the regulation of the host population. However, they do influence the equilibrium population densities of *I*, *Y* and *Z*, and the rate at which these equilibria are approached (Appendix S2).

This analysis therefore indicates that in the full seasonal model each year will consist of a period of exponential decay to the zero-steady state in the non-reproductive season and an approach to a positive steady state in the reproductive season.

### the effects of seasonal birth rates on long-term host–parasite dynamics

In the absence of disease, the full seasonal model (equation 1 with *S* > 0 and *I* = *Y* = *Z* = 0) can be solved analytically (see Appendix S3 for details). The analytical solution indicates that if the reproductive season is less than a critical length (*L* < *b*/*a*) then the host population will decay to zero over time, whereas if *L* > *b*/*a* then the long-term dynamics will be annual cycles that are repeated exactly each year.

**Fig. 1:**
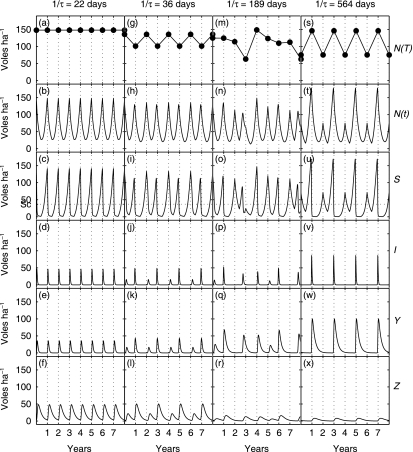
Predicted long-term dynamics for total population density (*N*) and individual component densities (susceptible, *S*, infected, *I*, recovered but not reproductive, *Y*, recovered and potentially reproductive, *Z*) for different values of τ. The thin dotted vertical lines denote the annual transition from the reproductive season to the non-reproductive season. The top row plots *N* through time, when it is sampled once a year only (at time *T*). The second row plots *N* as a function for continuous time *t*. Parameter values are the Kielder Forest field vole parameters (Table 1) with α = 4·3, β = 0·9, *f* = 0·225, 1/γ =14 days and 1/τ as detailed at the top of each row. *S*_C_ is the critical density of *S* required for *I* to increase (as detailed in the main text). All simulations started with *S* = 49 voles ha^−1^, *I* = 1 voles ha^−1^ and *Y* = *Z* = 0 voles ha^−1^.

If the model starts with a positive initial population density of infected individuals then it predicts three generally different types of long-term multiyear dynamics. The first is when the disease dies out over time, either because the susceptible host population is always less than *S*_C_ or because it is greater than *S*_C_ for such a short time each year that it results in net annual losses to the infected population density (as also shown by Ireland *et al*. 2004). In these scenarios, the long-term dynamics of the susceptible population density are the disease-free long-term dynamics. The second set of dynamics has the disease remaining endemic in the susceptible population, with annual cycles in the component population densities that are repeated exactly each year (e.g. Fig. 1a–f). With the third class of dynamics the disease again remains endemic, but the population density oscillations are not repeated exactly every year (e.g. Fig. 1g–x). In this case we observe either regularly repeated multiyear cycles (e.g. Fig. 1g–x) or quasi-periodic multiyear cycles (Fig. 1m–r, with a dominant period of 4 years).

The above results show that, providing the vole population can persist, the model always predicts oscillations in one (in the case of no-endemic disease) or all the population components, due to the seasonally varied birth rate. To aid clarity, below we define ‘regular annual cycles’ as the cases where the annual oscillations are repeated exactly every year (as in Fig. 1a–f) and ‘multiyear cycles’ as the cases where they are not (as in Fig. 1g–x).

### why does the model predict a variety of multiyear dynamics?

Figure 1(a–x) illustrates a transition from regular annual cycles to multiyear cycles, caused by reducing the rate of recovery of reproduction following infection, τ (increasing the time taken to recover), while holding all other parameter values constant. The decision to vary τ is not crucial and similar transitions could be illustrated by varying any other disease parameter.

**Fig. 2:**
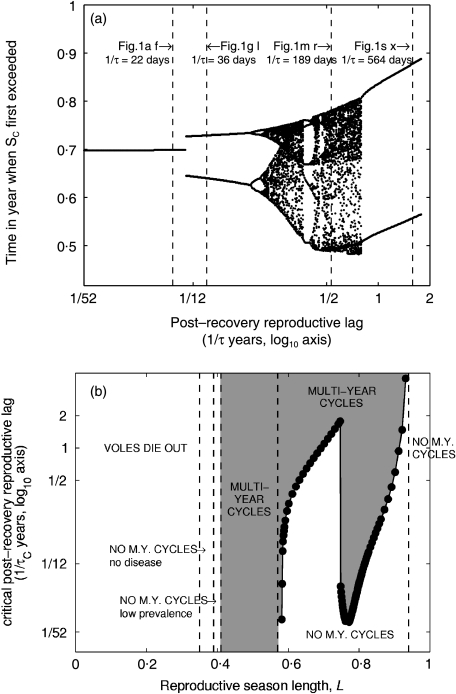
(a) Date in the year at which the infection threshold (*S*_C_) is first exceeded, as a function of 1/τ. Parameter values are as in Fig. 1. Dotted lines denote 1/τ values used for the examples in Fig. 1. The critical τ at which the multiyear cycles occur (τ_C_) is at 1/τ = 27 days in this example. (b) τ_C_ as a function of the length of the reproductive season, *L*. Regular annual cycles occur in the regions denoted ‘NO M.Y. CYCLES’. Other parameter values and initial conditions are as in Fig. 1m–r. We wrote a numerical code to run the model simulations for the full range of *L* with these parameter values, and calculate τ_C_ to three decimal places when it could be found (dots), or give details of the predicted dynamics when τ_C_ could not be found.

The key difference between the model predicting regular annual cycles and multiyear cycles is a difference in the rate at which the susceptible population recovers following a population crash. If this rate allows the period of oscillation of the host–disease dynamics to match the period of seasonal forcing (i.e. 1 year), usually with one or two annual disease oscillations per year, then the model settles on regular annual cycles. For example, when the time taken to recover reproductive function is sufficiently fast in Fig. 1 (1/τ≅ 22 days), then the predicted long-term dynamics are regular annual cycles (Fig. 1a–f) with the population densities of all four population components peaking once each year. With a slight increase in the time taken to recover reproductive function (1/τ≅ 36 days) the model predicts low-amplitude 2-year multiyear cycles (Fig. 1g–l). In this case the rate of recovery of susceptible individuals is sufficiently slow for the annual population dynamics not to be repeated in consecutive years. Figure 2a illustrates that as 1/τ is increased from 22 days (Fig. 1a–f) to 36 days (Fig. 1g–l) there is a critical point at which the multiyear dynamics change, from the susceptible population first exceeding *S*_C_ at the same time every year to this event occurring relatively late in one year and relatively early in the following year. Analysis of this transition reveals that the critical reproductive lag at which this transition occurs, τ_C_, is dependent upon the initial population densities used in simulations. In particular, between 1/τ = 23 days and 1/τ = 66 days in Fig. 2a the model either predicts regular annual cycles or 2-year multiyear cycles, depending on initial conditions; mathematically, there is a hysteresis in the bifurcation diagram (not shown). However, the remainder of the bifurcation diagram is unaffected by initial conditions.

As 1/τ is increased further in Fig. 2a the difference between the dates of the early and late disease outbreaks diverge until there is a period doubling bifurcation, followed closely by further bifurcations and chaos, at around 1/τ = 80 days in this example. Figure 1m–r is an example from the chaotic region in Fig. 2a (1/τ = 189 days). In this scenario, the times in the year at which the different component populations peak are different in different years (Fig. 1m–r). Extensive numerical simulations suggest that the transition from regular annual cycles to multiyear cycles is always associated with the susceptible population density exceeding *S*_C_ later in the reproductive season. Reducing τ and/or *f* (which increases the recovery lag) and varying other parameters (more details in next section) can all reduce the rate at which the susceptible population recovers from low densities. This, in turn, can induce multiyear cycles.

The smallest value of τ (1/τ≅ 1·6 years) in Fig. 1 (Fig. 1s–x) causes the population dynamics to settle on regular 2-year cycles. In this case the infected population density peaks every 2 years exactly (Fig. 1v). This is because it takes 2 years for the susceptible population density to induce a sufficiently large outbreak in the infected population density to cause the susceptible population density to crash. In other scenarios (not illustrated) a disease outbreak may reduce the susceptible population to such an extent that it may take longer than 1 year for the susceptible population density to recover past *S*_C_, also resulting in multiyear cycles. In a few rare scenarios, increasing the time it takes for the susceptible population to recover from a population crash can result in a transition from multiyear cycles to regular annual cycles. Such transitions are associated with a reduction in the number of disease outbreaks per year. For example, when the model predicts multiyear cycles with the disease breaking out on average twice a year, reducing the rate of recovery of the susceptible population following a population crash (for instance, by reducing τ) can then cause the disease to break out only once a year and can induce regular annual cycles.

### the importance of seasonal forcing in determining the multiyear dynamics

In Fig. 2b we illustrate the importance of seasonal reproduction for the multiyear dynamics by varying the relative lengths of the reproductive and non-reproductive season and plotting the critical τ (τ_C_) required to induce multiyear cycles (with all other parameters held constant and starting from identical initial conditions). Slightly above the critical reproductive season length necessary for the voles to exist (*L* = *b*/*a* = 0·35 in Fig. 2b) is a region in which the hosts exist at densities that are too low to allow the disease to become endemic (0·35 < *L* < 0·39), and slightly beyond this is a small region in which the disease persists at such low prevalence that it has only a minor impact on the host population (0·39 < *L* < 0·41).

Once the reproductive season length is sufficiently long there is a region (0·41 < *L* < 0·57) in which the disease induces multiyear cycles for all values of τ. Note, however, that in this region the presence of the disease is still necessary to induce multiyear cycles. For higher reproductive season lengths there is a region (0·57 < *L* < 0·94) in which τ_C_ is a non-monotonic function of reproductive season length. Analysis of the numerical dynamics along the τ_C_ line reveals that the ‘bump’ (at *L* = 0·78) corresponds to the transition between the disease breaking out once a year (lower *L*) and twice a year (higher *L*). Therefore, once the reproductive season is sufficiently long, the disease can have a significant impact on the stability of the multiyear host population dynamics.

Finally, as the length of the non-reproductive season becomes very short (*L* > 0·94), there is a region in which multiyear cycles do not occur for any values of τ (Fig. 2b). This illustrates our general finding that the non-reproductive season must have a sufficiently large impact on the population dynamics for the model to predict multiyear cycles.

### systematic sampling of parameter space: how common could disease-induced cycles be?

In this section we describe the regions of disease parameter space that predict disease-regulation and multiyear cycles in the population dynamics. This will indicate the sorts of values of τ and *f* necessary to induce multiyear cycles.

**Fig. 3:**
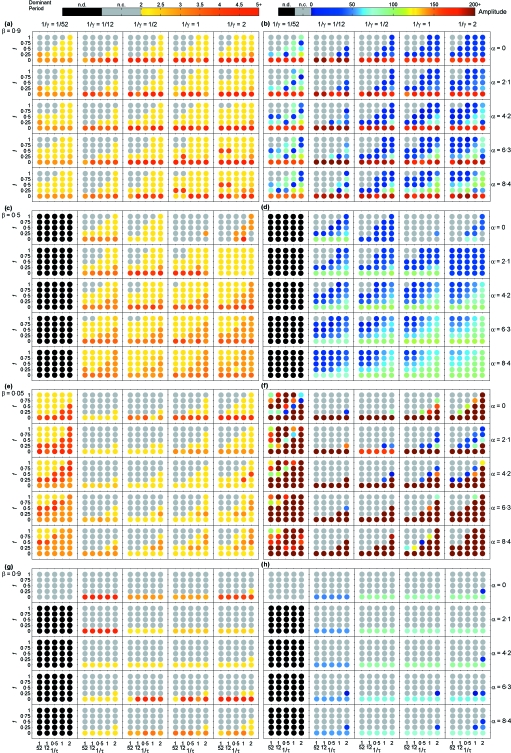
Effects of variation in disease parameters on the period (left column) and amplitude (right column) of the multiyear cycles predicted by model (1), for fixed values of β (labelled on plot) and different rodent population parameters (Table 1). Parameter values are: (a, b) Kielder forest field voles; (c, d) Manor Wood bank voles; (e, f) French common voles; (g, h) northern Fennoscandian field voles. Results for all values of β, for all rodent population parameters, are given in Appendix S4. In the colour bar ‘n.d.’ denotes simulations in which the disease prevalence decays to zero during the course of the simulation. In these cases the susceptible population density exhibits regular annual cycles. Similarly, ‘n.c.’ denotes simulations in which disease remains endemic in the population and all four population components exhibit regular annual cycles. The dominant period of the multiyear dynamics was measured by spectral analysis (using fast Fourier transform) of 256 years of equilibrium population data, measured annually (see Turchin 2003 for a review). The amplitude was the difference between the maximum and the minimum total population density in this data set. For brevity we do not distinguish between regularly repeated multiyear cycles and irregular (pseudo-periodic) multiyear cycles.

Figure 3 shows how the dominant period or amplitude of the multiyear cycles predicted by the model are affected by variation in four of the disease parameters for four sets of rodent parameter values (see Appendix S4 for the results of the full analysis). For illustration purposes we chose sufficiently high β values for each set of rodent parameters in Fig. 3 so that the disease can become endemic for variation in the other parameter values. Varying β has less effect on the model predictions than varying the other parameter values, provided that it is sufficiently high for the disease to become endemic in the population. For brevity we omit showing representative results for the grey-sided voles data set (Table 1), as they were very similar to the results obtained for the field voles in Fennoscandia (see Appendix S4 for these results).

Each subfigure in Fig. 3 has a series of plots of the dominant period or amplitude of the multiyear cycles. We plot these as a function of 1/τ and *f* for 25 sets (per subfigure) of the other parameters. The individual 1/τ against *f* plots all share some basic features. Regular annual cycles are predicted when the rate of recovery of reproductive function is fast and *f* is large (1/τ = 7 days, *f* = 1) and multiyear cycles are predicted when the rate of recovery of reproductive function is slow and *f* is small (1/τ ≥ 1 year, *f* = 0) (Fig. 3). Between these two corners of 1/τ – *f* space there is a bifurcation structure similar to that illustrated in Fig. 2a, with a region of low-amplitude, period-2 multiyear cycles close to the stability transition, and behind it a region of period-doubling and chaos, associated with larger amplitude cycles. Generally, increasing the time taken to recover reproductive function results in multiyear population cycles being predicted at higher values of *f*. Numerical investigation showed that the dynamics predicted when the reproductive delay is shortest (1/τ = 7 days) are qualitatively similar to those when recovery of reproductive function following infection is instantaneous [τ = ∞ (not shown)], in which case the model collapses down to a classical host–parasite framework (Anderson *et al*. 1981). Increasing α generally increases the size of the region predicting multiyear cycles in the individual 1/τ against *f* plots. Note, however, that cycles can be predicted by the model even when infection causes no increase in the mortality rate (α = 0). The effect of variation in γ appears more complicated, with the size of parameter space predicting multiyear cycles sometimes initially contracting, and then expanding, as 1/γ is increased from 7 days to 2 years (explained below).

In rodent populations, long reproductive delays following infection (1/τ and 1/γ ≥ 1 year) would mean that most rodents would die before recovering their reproductive ability. Similarly, a small *f* would mean that even if individuals did recover they would not make a large per capita contribution to the susceptible population. Low values of τ, γ and *f* therefore cause the time taken for the susceptible population to recover from a disease outbreak to be relatively long and, as shown in the previous section, this can induce multiyear cycles. However, a rapid recovery rate from infection (1/γ ≤ 1 month) can also increase the time it takes the susceptible population to exceed *S*_C_. This is because increasing γ also increases *S_C_*, and if the recovery rate is sufficiently rapid (1/γ ≤ 1 month) *S*_C_ can get close to, but still be less than, *K*. In these scenarios, density dependence slows down the growth of the susceptible population as it approaches *S_C_*. This increases the date at which the infection threshold is exceeded, and this extra time delay is sufficient to cause multiyear cycles in these cases.

The importance of the host population parameters to the model predictions is also apparent in Fig. 3. A relatively high proportion of simulations with the Kielder Forest and Manor Wood parameters (when disease is endemic) predict multiyear cycles (Fig. 3a–d). This is associated with their lower (estimated) maximum per capita growth rates (*r* = 2·5 and 1·8, respectively) which increase the time taken for the susceptible population to recover following a disease outbreak. The high maximum population density of the French common vole populations (*K* = 2000 voles ha^−1^) allows them to support endemic infections with lower infection rates (Fig. 3e,f). In this scenario, multiyear cycles are associated with slow rates of recovery of reproductive function and small *f*, for all values of β analysed. The northern Fennoscandian field vole and Hokkaido grey-sided vole populations have the lowest estimated maximum population densities and highest estimated maximum growth rates. Endemic infections are predicted only at high infection rates for these parameter combinations (Fig. 3g,h). Multi-year cycles are also less commonly predicted for these populations and are usually seen only when the rate of recovery of reproductive function is very slow or *f* is extremely low (1/τ = 2 years, *f* = 0) (Fig. 3g,h). The model therefore predicts that disease-induced effects on fecundity are more likely to induce multiyear cycles in rodent populations if those populations recover more slowly from low density than those rates estimated for the Fennoscandia and Hokkaido populations (*r* < 5).

### detailed analysis into the possible effects of cowpox in the kielder forest system

We performed a more detailed exploration of the region of model parameter space that corresponds to cowpox virus in Kielder Forest field voles. Our aim was to estimate the τ and *f* values that a disease such as cowpox virus, and potential secondary, possibly chronic infections, would need in order to induce multiyear cycles in their host populations. We estimated disease parameters from studies of the cowpox virus in the Kielder Forest and Manor Wood rodent populations (Telfer *et al*. 2002, 2005; Cavanagh *et al*. 2004; Burthe *et al*. 2006, in press) and provide details in Appendix S1.

**Fig. 4:**
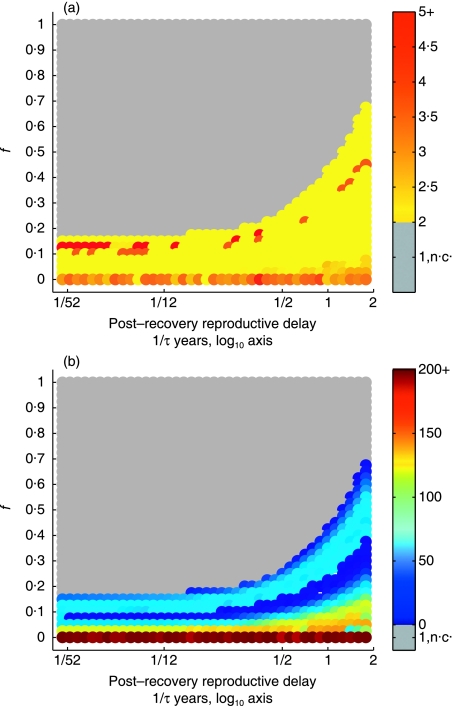
Dominant period (a) and amplitude (b) predicted by the model as 1/τ and *f* are varied for the Kielder Forest field vole parameters (Table 1), with α = 4·3, β = 0·9 and 1/γ = 28 days. Colour bar conventions are as in Fig. 3. Dominant period and amplitude were measured as detailed in the legend of Fig. 3.

The model predicts that if cowpox virus infection has a sufficiently large impact on rodent fecundity following recovery from infection (*f* = 0·2) then the host population will exhibit multiyear cycles for all reproductive lags (Fig. 4). However, as *f* is increased from 0·2, the delay in reproduction required to produce multiyear cycles increases until, beyond *f* = 0·7, the model does not predict multiyear cycles for any values of τ analysed. Therefore, for the range of τ and *f* used in Fig. 4, the model predicts that in order to induce multiyear cycles in the Kielder Forest field voles, a disease such as cowpox would have to induce significant chronic reductions in fecundity following recovery of reproductive function (*f* < 0·4). We explored plots of 1/τ against *f* for different values of the other disease parameters. This caused some changes in the size of the parameter space predicting multiyear cycles and in the period and amplitude of the multiyear cycles; however, the results did not differ qualitatively from those in Fig. 4.

### does the model predict delayed density-dependent reproductive timing and seroprevalence in cases of disease-induced multiyear cycles?

We investigated whether two phenomena that have been observed in studies in Kielder Forest are predicted by the model, namely delayed density-dependent seroprevalence of cowpox virus (Cavanagh *et al*. 2004) and an apparent delay in the onset of the reproductive season (Ergon *et al*. 2001, 2003). Although season length is fixed in the model, the growth rate of the susceptible population at the onset of the reproductive season differs depending on the relative sizes of the component population densities. We measured the ‘effective onset’ of the reproductive season as the time in the year at which the susceptible population achieves a growth rate equal to what it would have achieved at the actual onset of the reproductive season, if the disease was absent. The effective delay in the onset of the reproductive season is therefore the difference between this ‘effective onset’ and the actual onset of the reproductive season. We then measured the strength of linear correlations between the effective delay in onset of the reproductive season and population densities and at varying times in the past. We also investigated whether seroprevalence at the start of the reproductive season, measured as the proportion of the total population in the *I*, *Y* and *Z* classes, was correlated significantly with the population density at various times in the past. We selected simulations with quasi-periodic multiyear cycles, from our detailed exploration of parameter space outlined in the previous section, to investigate these relationships. This is because quasi-periodic multiyear cycles give a range of values for these output variables when the population is sampled at the same time for multiple years. This avoids having to incorporate noise into the model to obtain a range of different values for which to analyse correlations.

**Fig. 5:**
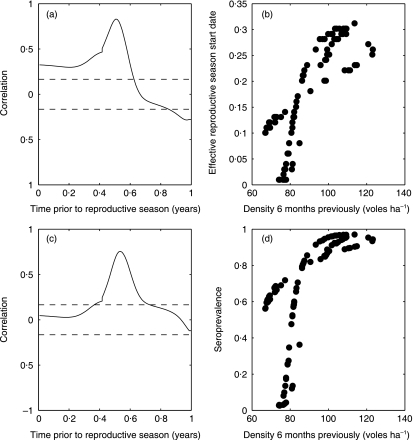
(a) Correlation between ‘effective start date’ of the reproductive season, as defined in the main text, and the population density at varying times in the past. The dashed lines denote the correlation coefficient at which the probability of no significant relationship is *P* = 0.05. (c) The same analysis as in (a), but correlating seroprevalence at the start of the reproductive season with past population densities; (b, d) ‘effective season start date’ and seroprevalence plotted against past population density for the corresponding highest significant positive correlation in (a) and (c), respectively. Zero on the vertical axis in (b) corresponds to no effective delay in the onset of the reproductive season. Parameter values are the same as for Fig. 1m–r.

Figure 5 illustrates, for the Kielder Forest field vole parameters, that both delayed density-dependent reproductive timing and seroprevalence can be predicted by the model when it predicts quasi-periodic multiyear cycles. Figure 5a shows that the effective date at which the reproductive season starts is correlated most highly with the population density around 6 months previously. Figure 5b plots the data for the time at which this most significant correlation occurs. Seroprevalence is also correlated most significantly with the population density around 6 months previously. This means that the model predicts a high seroprevalence (over 90% of individuals with cowpox antibodies in Fig. 5d) and a long delay in the onset of the reproductive season (around 4 months in Fig. 5b) at the actual onset of the reproductive season, if total rodent density 6 months previously was high, and vice versa.

## Discussion

Our key finding is that both delayed reproduction following microparasitic infection and reduced fecundity following recovery from infection can destabilize the multiyear dynamics of host populations. It is already known that disease-induced reductions in fecundity can destabilize host population dynamics (Anderson *et al*. 1981; Dobson & Hudson 1992; White *et al*. 1996; Boots & Norman 2000). Dobson & Hudson's (1992) model of the red grouse interaction with a parasitic nematode (see also Hudson *et al*. 1998), for example, predicts population cycles when infection-induced reductions in host fecundity are sufficiently higher than infection-induced reductions in survival (see Hudson *et al*. 1998; Lambin *et al*. 1999; Tompkins & Begon 1999; Turchin 2003; Redpath *et al*. 2006 for discussion on the empirical support for this hypothesis). To our knowledge, no studies have incorporated a period of no reproduction following recovery from infection. This is obviously another mechanism by which the birth rate of the population can be reduced, and so it is not surprising that its effects should be destabilizing. However, in contrast to previous non-seasonal models predicting disease-induced cycles, the effects of the disease cannot induce sustained population cycles in our model in the absence of seasonal forcing. This supports studies that have shown that seasonality is a key determinant of the multiyear dynamics of rodent populations (Hansen, Stenseth & Henttonen 1999; Stenseth 1999; Stenseth *et al*. 2003, 2006; Hornfeldt 2004; Bierman *et al*. 2006; Smith *et al*. 2006b).

We included a seasonally varying birth rate because it features so widely across rodent populations with cyclic dynamics. Seasonal forcing has been incorporated in a variety of ways into other theoretical studies, most commonly in the infection rate (Altizer *et al*. 2006). Other parameters in our model would perhaps be modelled more realistically as seasonally varying, and the incorporation of such seasonality would probably alter the predicted dynamics. The wide range of other studies, showing that exogenously generated oscillations can resonate with the endogenous oscillations within a biological system (Altizer *et al*. 2006), suggests to us that the incorporation of such seasonality could also lead to multiyear population cycles. It also seems likely that the incorporation of demographic and environmental stochasticity would tend to destabilize the dynamics predicted by our model (Greenman & Benton 2003, 2005; McKane & Newman 2004; Alonso, McKane & Pascual 2007) and would probably increase the size of parameter space predicting cycles. Future studies incorporating demographic stochasticity are also needed to investigate the model scenarios in which the population density declines repeatedly to very low values; in reality these could result in the local extinction of the disease or of the disease and the host population together.

To date, it has been shown that acute cowpox virus infections affect subadult maturation rates only in bank voles and wood mice (Telfer *et al*. 2005). Empirical studies should investigate the generality of these findings for other diseases and other rodent populations, as we have shown here that such effects can have important population dynamic consequences. Our findings are especially relevant to taxa such as microtine rodents, in which the subadults adapt their life history strategy as a consequence of their general health and their environment (Lambin & Yoccoz 2001). The impacts of infection on reproduction in other subclasses of animals (e.g. adults), the long-term effects of infection on fecundity and the effect of primary infections on the susceptibility to secondary infections (which may themselves affect fecundity) are currently being investigated for cowpox and other microparasitic diseases in the Kielder Forest field vole populations.

The findings of Telfer *et al*. (2005) also raise the question of whether the incorporation of different life history stages would significantly alter our model predictions. For example, a more realistic representation of the reported delayed-maturation effects would be to model the disease dynamics in the juvenile, subadult and adult populations separately, with cowpox infection delaying the maturation of the subadults to adults. It seems likely that this modification would tend to have a stabilizing effect on the multiyear host dynamics, as only a fraction of the total host population would be affected significantly by the disease. Preliminary modelling investigations have supported this conjecture.

For the Kielder Forest field vole–cowpox interactions, our results show that the disease could cause multiyear cycles, provided that its effects on fecundity are sufficiently strong (Fig. 4). Ireland *et al*. (2004, 2007) found, using a seasonally forced SIR (susceptible, infected, recovered) model, that cowpox virus infections were unlikely to cause multiyear cycles in UK bank vole populations. However, Ireland *et al*. (2004, 2007) did not incorporate the two effects of disease on reproduction upon which we focus in this study. These differences lead to damped oscillations in the reproductive season dynamics rather than a monotonic approach to equilibrium values, and are the difference between the model predicting repeated annual cycles and multiyear cycles in the full seasonal model.

The fact that two previously unexplained phenomena, delayed density-dependent seroprevalence and onset of reproduction in the spring, are predicted by our model (Fig. 5) supports the hypothesis that diseases may play a role in the multiyear population dynamics of Kielder Forest field voles (Ergon 2003; Cavanagh *et al*. 2004). In a previous study we showed that a delayed density-dependent onset of the reproductive season could induce multiyear cycles in host populations (Smith *et al*. 2006b). In contrast, in this study we have shown that delayed density-dependent seroprevalence and effective onset of the reproductive season could simply be epiphenomena of disease-induced multiyear cycles. However, it is unknown whether similar disease dynamics would be predicted if some other mechanism is generating host population cycles (such as a predator–prey interaction) and an asymptomatic disease simply tracks the population cycles. This is a natural direction for future theoretical studies. Our Kielder Forest case study also predicts that if disease-driven multiyear cycles are occurring in the field, then these could be indicated by multiyear variation in other phenomena that could be measured or looked for in data sets already collected, such as the date in the year at which seroprevalence peaks.

Could repeated multiyear cycles in certain microtine rodents be caused by diseases acting singly or in combination? The wide range of realistic parameter values for which disease-induced cycles are predicted by our model (Fig. 3) suggests that such mechanisms are at least plausible.

## Supplementary material

The following supplementary material is available for this article.

Appendix S1 Parameter estimates for different rodent populations.

Appendix S2 Mathematical analysis of non-seasonal models.

Appendix S3 Analysis of the critical season length for the existence of voles.

Appendix S4 Results of systematic analysis of disease parameter space for five different rodent population parameters.

This material is available as part of the online article from: http://www.blackwell-synergy.com/doi/full/10.1111/j.1365-2656.2007.01328.x (This link will take you to the article abstract.)
