# Supplementary material for: Disease effects on reproduction can cause population cycles in seasonal environments
Source: J Anim Ecol. 2008 Mar;77(2):378–89. doi: 10.1111/j.1365-2656.2007.01328.x (PMC2408661; doi:10.1111/j.1365-2656.2007.01328.x)
Supplement: Appendix S1 — Parameter estimates for different rodent populations. [file jae0077-0378-SD1.doc]

**Supplementary material**

**Appendix S1. Parameter estimates for different rodent populations**

*S1.1. UK field voles (*Microtus agrestis *L.) in grassland habitat (Kielder Forest)*

Kielder Forest is a man-made spruce plantation (620km^2^) situated on the border between Scotland and England (53˚13’N, 2˚33’W). Field voles inhabit the grasslands that have formed in the woodland clear-cuts which are dominated by *Deschampsia caespitosa* Beauv., *Agrostis tenuis* Sibth., and *Juncus effusus* L . Field vole densities in the forest have been shown to fluctuate cyclically with a 3-4 year period (Lambin, Petty & MacKinnon, 2000).

Instead of calculating a maximum per capita birth rate, $a$, directly we calculated it using $a=(r+b)/L$ where the maximum per-capita population growth rate, $r$, reproductive season length, $L$, and per capita mortality rate, $b$, were estimated from data. Burthe et al. (in press) give a median cowpox-free monthly survival figure of 0.735 which implies a per capita death rate of $b=3.7$. The data we use to estimate maximum per capita growth rates and the length of the reproductive season is a collection of longitudinal mark-recapture estimates, taken approximately monthly from 21 different sites over differing periods of time. This data was collected during various different studies from 1996 to 2005 (Lamb*in et a*l., 2000; Erg*on et a*l., 2001; Ergon, 2003; Cavana*gh et a*l., 2004). Following the protocol of Turchin and Ostfeld (1997) we calculated monthly per capita growth rates ($pgr$) from this data set (485 data points) and calculated $r$ as the intercept of the linear regression between population density ($N_{t}$) and per capita growth rate ($pgr=-0.03N_{t}+2.5\Rightarrow r=2.5$).

Visual inspection of the monthly per capita growth rates also showed that the reproductive season generally started at the beginning of March and continued until the end of September. This was also found by Ergon et al. (2001) for sites that were in the increasing phase of the population cycle. This gave a reproductive season length of seven months. Our maximum population size of $K=250$ voles ha^-1^ was also taken from this population data.

*S1.2. Estimates for cowpox virus in Kielder Forest*

From Burthe et al. (in press) we estimated that$\alpha=4.3$. Given this value, the high disease free mortality rate ($b=3.7$), and the fact that the recovery rate is also likely to be quite rapid ($1/\gamma=28$ days in Manor Wood bank voles) (Blasdell, 2006), together mean that for values of $\beta$ estimated by Begon et al. (1998; 1999) for bank voles in Manor Wood ($\beta=0.05$ makes voles ha^-1^ whereas $K=250$ voles ha^-1^). However, cowpox virus seroprevalence in the Kielder Forest field voles is over an order of magnitude higher than that recorded in Manor Wood bank voles (Beg*on et a*l., 1999; Cavana*gh et a*l., 2004). We therefore assume that infection rate is also an order of magnitude higher ($\beta=0.9$) which gives similar maximum seroprevalences in simulations to the field data. We also assume that the recovery rate of field voles from cowpox virus infection is similar to that found for bank voles in Manor Wood ($1/\gamma=28$ days) (Blasdell, 2006). Numerical analysis into the effects of varying $\gamma$ on the model predictions showed that large amplitude (>50 voles ha^-1^) multi-year cycles were only predicted when $\gamma$ was sufficiently high ($1/\gamma>36$ days).

*S1.3. UK bank voles (*Clethrionomys glareolus *Schreber) in mixed woodland habitat (Manor Wood)*

The Manor Wood and Rake Hey sites are two 1ha mixed woodland sites in North West England (Manor Wood: N53˚19’ W03˚03’; Rake Hey: N53˚20’ W03˚02’). In this study we combine the data for both sites and refer to this combined data set as “Manor Wood”. Bank vole densities have been monitored monthly at these sites using mark-recapture techniques since 1995 (Telfer et al., 2005). Time series analysis of this data set suggests a tendency towards biennial cycles in the bank vole population (Carslake et al., 2005).

We used the same technique as for the Kielder Forest data to calculate parameters for the maximum per capita birth rate, reproductive season length and maximum population size. We estimated a monthly survival rate of 0.77 from Telfer et al. (2002) to give a per capita death rate of $b=3.1$.

*S1.4. Field voles (*M. agrestis*) in Fennoscandian grassland*

The Fennoscandinavian rodent populations have perhaps been the most extensively studied cyclic microtine populations in recent decades. Various different species across a wide range of habitats and climates exhibit multi-year fluctuations in abundance with a 3-5 year periodicity (Turchin, 2003). Several previous theoretical studies have estimated parameter values and parameterised models of these populations. In this study we obtained representative parameter estimates from Turchin and Hanski (1997) and Hanski et al. (1993).

*S1.5. Japanese grey-sided voles (*Clethrionomys rufocanus *Sundevall) in woodland (Hokkaido)*

The grey-sided vole populations towards the north-east of the island of Hokkaido exhibit multi-year density cycles. The parameters used in this study were taken from Yoccoz et al. (1998) who parameterised a seasonal demographic model for a population from mixed natural forest at Mizuho (43˚42’N, 142˚39’E) exhibiting 2-year multi-year cycles.

*S1.6. French common voles (*Microtus arvalis *Pallas) in agricultural habitat*

Some common vole populations in south-western France exhibit regular 3-year multi-year cycles (Lambin, Bretagnolle & Yoccoz, 2006). Our parameter estimates for the maximum per capita growth rate and the maximum population size come from Lambin et al. (2006). We estimate monthly survival ($b=3.1$) and the length of the reproductive season (8 months) in line with the other populations (above).

**Appendix S2. Mathematical analysis of non-seasonal models**

In this appendix we analyse mathematically the non reproductive season and the reproductive season equations separately. We treat each system of equations as if the season were infinitely long and look for steady states of interest and analyse their local stability. Our intention is to demonstrate the predicted dynamics of the equations in the absence of seasonal forcing.

*S2.1. Non-reproductive season dynamics*

The equations for the non-reproductive season are the simplest to analyse and are given by,

$\frac{dS}{dt}=-\beta SI-bS$, (B1a)

$\frac{dI}{dt}=\beta SI-(b+\alpha+\gamma)I$, (B1b)

$\frac{dY}{dt}=\gamma I-(b+\tau)Y$, (B1c)

$\frac{dZ}{dt}=\tau Y-bZ$, (B1d)

with parameter definitions given in the main paper. The only realistic steady state for these equations is when all component population densities are zero ($S=I=Y=Z=0$). The stability of this steady state is analysed in the standard way by linearising equations B1 about this steady state to give the stability matrix:

(B2)

The characteristic polynomial is

. (B3)

Therefore all four eigenvalues are real and negative, implying that the steady state is stable.

*S2.2. Reproductive season dynamics*

The equations for the reproductive season are more complicated to analyse and are given by

$\frac{dS}{dt}=a(S+fZ)(1-qN)-\beta SI-bS$, (B4a)

$\frac{dI}{dt}=\beta SI-(b+\alpha+\gamma)I$, (B4b)

$\frac{dY}{dt}=\gamma I-(b+\tau)Y$, (B4c)

$\frac{dZ}{dt}=\tau Y-bZ$. (B4d)

This system of equations has three realistic steady states. One of these is when all population components are of zero density ($S=I=Y=Z=0$) and another is when there is no disease in the system ($I=Y=Z=0$) and the susceptible population density is at carrying capacity ($S=K=(a-b)/qa$). The third steady state is when disease is endemic in the population. The population density of susceptibles at this steady state is

$\tilde{S}=(b+\alpha+\gamma)/\beta$. (B5a).

Furthermore the equilibrium densities for the $Y$and $Z$classes can be expressed in terms of the equilibrium density of infecteds ($\tilde{I}$) as

, and (B5b)

, respectively. (B5c)

Substituting these steady state densities into equation B4a and simplifying gives

, (B5d)

with $\xi=\gamma/(b+\tau)$ and $\theta=\tau/b$. Equation B5d can be solved to give two different values for $\tilde{I}$. Furthermore, since $K-\tilde{S}$ is positive and the coefficient of $\tilde{I}^{2}$ is negative we know equation (B5d) can be solved to give at least one positive equilibrium value for $\tilde{I}$. To determine whether equation B5d predicts one or two positive equilibrium values for $\tilde{I}$ we re-write it as

(B5e)

which shows that the two values for $\tilde{I}$ are predicted at the intersection between a linear expression in $\tilde{I}$ (left hand side expression) and a parabola in $\tilde{I}$ (right hand side expression). The left hand side expression is positive when $\tilde{I}=0$ and $K>\tilde{S}$ and has a negative slope for increasing $\tilde{I}$ whereas the right hand side expression equals zero when $\tilde{I}=0$ and has a minimum at $\tilde{I}>0$ (since $1-1/\tilde{S}q<0$). Straightforward plotting of these as functions $\tilde{I}$of confirms that equation (B5d) must predict one positive and one negative value for $\tilde{I}$.

The stability matrix for equations (B4) is

(B6)

The characteristic equation at the zero-steady state is simply

(B7)

which is unstable providing $a>b$, otherwise the zero-steady state is stable.

The characteristic equation when the susceptible population is at carrying capacity and the disease is absent is

(B8)

This steady state is therefore unstable if $K>\tilde{S}$, otherwise the steady state is unstable and the disease-endemic steady state is stable (see below).

The characteristic equation for the disease-endemic steady state is cumbersome and is omitted here for brevity. We have so far been unable to determine, using this equation, whether or not this steady state is stable. It is possible to show that the steady state is stable when parameters $f$, $\tau$ or $\gamma$ equal zero. Moreover, Norman et al. (1994) studied a model that is the same as ours if we assume $f=1$ and $\tau=\infty$, and showed a stable disease-endemic steady state. More generally, if we assume that the steady state does become unstable in some region of parameter space then there is a sign change in either a real eigenvalue or the real part of a complex eigenvalue. Therefore, at the point at which stability changes, the critical eigenvalue is , with $\omega$ real. Substituting this into the characteristic equation gives.

, (B9)

where $A$, $B$, $C$, and $D$ are functions of the model parameters with rather complicated algebraic forms. If a real eigenvalue changes sign then $\omega=0$ which implies that $D=0$. It is possible to show that $D>0$ (Maple code demonstrating this is available from the corresponding author on request) and, therefore, that the disease endemic steady state does not become unstable through a real eigenvalue becoming positive.

In the case where the real part of a complex eigenvalue changes sign, the imaginary part of (B9) implies that . Since we know that $\omega\neq0$ this must occur when . Substituting this back into the real part of (B9) implies that the expression

(B10)

must be zero. Extensive numerical calculations of (B10) for a wide range of parameter values (Maple code to run these calculations is available from the corresponding author on request) suggest that (B10) is always negative which would imply that the disease-endemic steady state is stable. However we have been unable to confirm this analytically.

**Appendix S3 - Analysis of the critical season length for the existence of voles**

Here we derive an equation for the multi-year host dynamics in the absence of disease and give conditions for the local stability of the equilibrium dynamics.

The ordinary differential equation for the dynamics in the reproductive season in the absence of disease is

$\frac{dS}{dt}=aS(1-qS)-bS$ (C1)

where we assume throughout that $a>b$. Equation (C1) can be solved exactly to give

$S(t)=\frac{(a-b)S(0)e^{(a-b)t}}{(a-b)-qaS(0)(1-e^{(a-b)t})}$ (C2)

where S(t) is the susceptible population density at time t and S(0) is the susceptible population density at time 0.

The ordinary differential equation for the dynamics in the non reproductive season is

$\frac{dS}{dt}=-bS$ (C3)

which has the simple solution

$S(t)=S(0)e^{-bt}$. (C4).

Equations (C2) and (C4) can be combined to give a difference equation for the population size measured once per year

$S_{T+1}=\frac{(a-b)S_{T}e^{aL-b}}{(a-b)-qaS_{T}(e^{-b(1-L)}-e^{aL-b})}=F(S_{T})$ (C5)

where S_T_ is the susceptible vole population density at discrete time, T, which is the point at which the reproductive season ends and the non-reproductive season begins, and L is the length of the reproductive season, where $0<L<1$.

We define as the susceptible population density at which losses in the non-reproductive season are exactly compensated for by the gains in the reproductive season. Substituting this into (C5) and rearranging gives the two steady state solutions $\hat{S}=0$ and

. (C6)

Since equation (C5) is a first order difference equation its steady states are locally stable if and only if

. (C7)

When $\hat{S}=0$

, (C8)

which is positive and less than 1 (stable) if $L<b/a$ and greater than 1 (unstable) if $L>b/a$.

At the positive steady state (C6) the stability condition (C7) becomes,

(C9)

Given $a>b$ and $0<L<1$, expression (C9) must always be positive. Moreover it approaches positive infinity as $L\to0$. To determine whether expression (C9) is decreasing in the range $0<L<1$ we need to analyse its derivative with respect to L, which is

$\frac{a(-e^{L(2b-a)-b}+2e^{b(L-1)}-e^{aL-b})}{(e^{b(L-1)}-e^{aL-b})^{2}}$. (C10)

For (C9) to decrease with increasing $L$ therefore requires that

. (C11)

Since $1<e^{L}<e$ and , inequality (C11) must be true and (C9) is decreasing in the range $0<L<1$. When $L=b/a$

.

Therefore (C9) is a decreasing function of $L$ that starts at positive infinity when $L=0$, crosses 1 at $L=b/a$, and remains positive as $L$ increases to 1. The positive steady state solution (C6) must therefore be locally unstable when $L<b/a$ and locally stable when $L>b/a$. Furthermore, when $L>b/a$, small perturbations from this steady state return to the steady state monotonically since (C9) is always greater than zero.

**Appendix S4. Results of systematic analysis of disease parameter space for five different rodent population parameters.**

Figures are as detailed in Fig. 3 but for different values of $\beta$.

**Kielder Forest field voles**

**
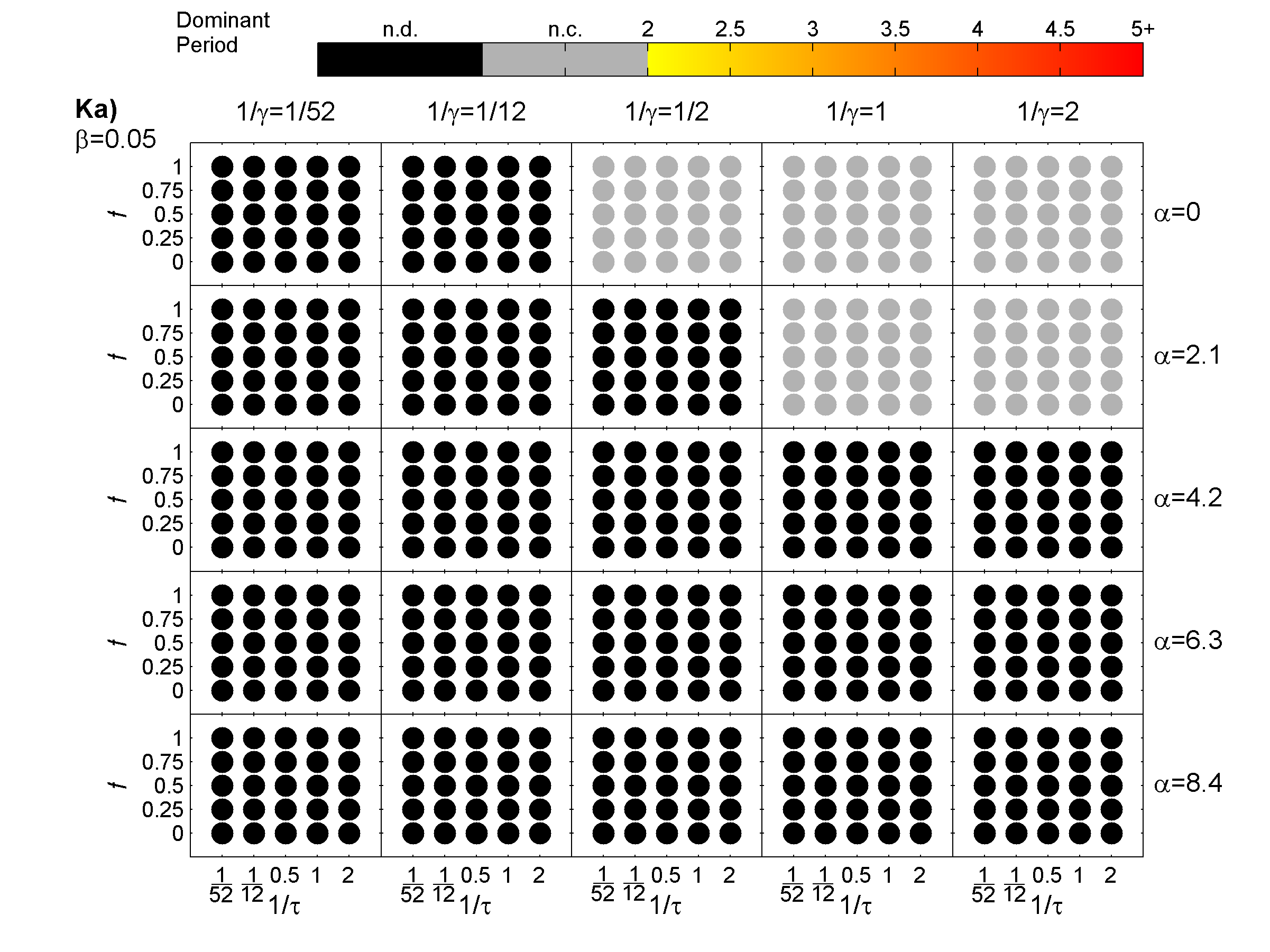

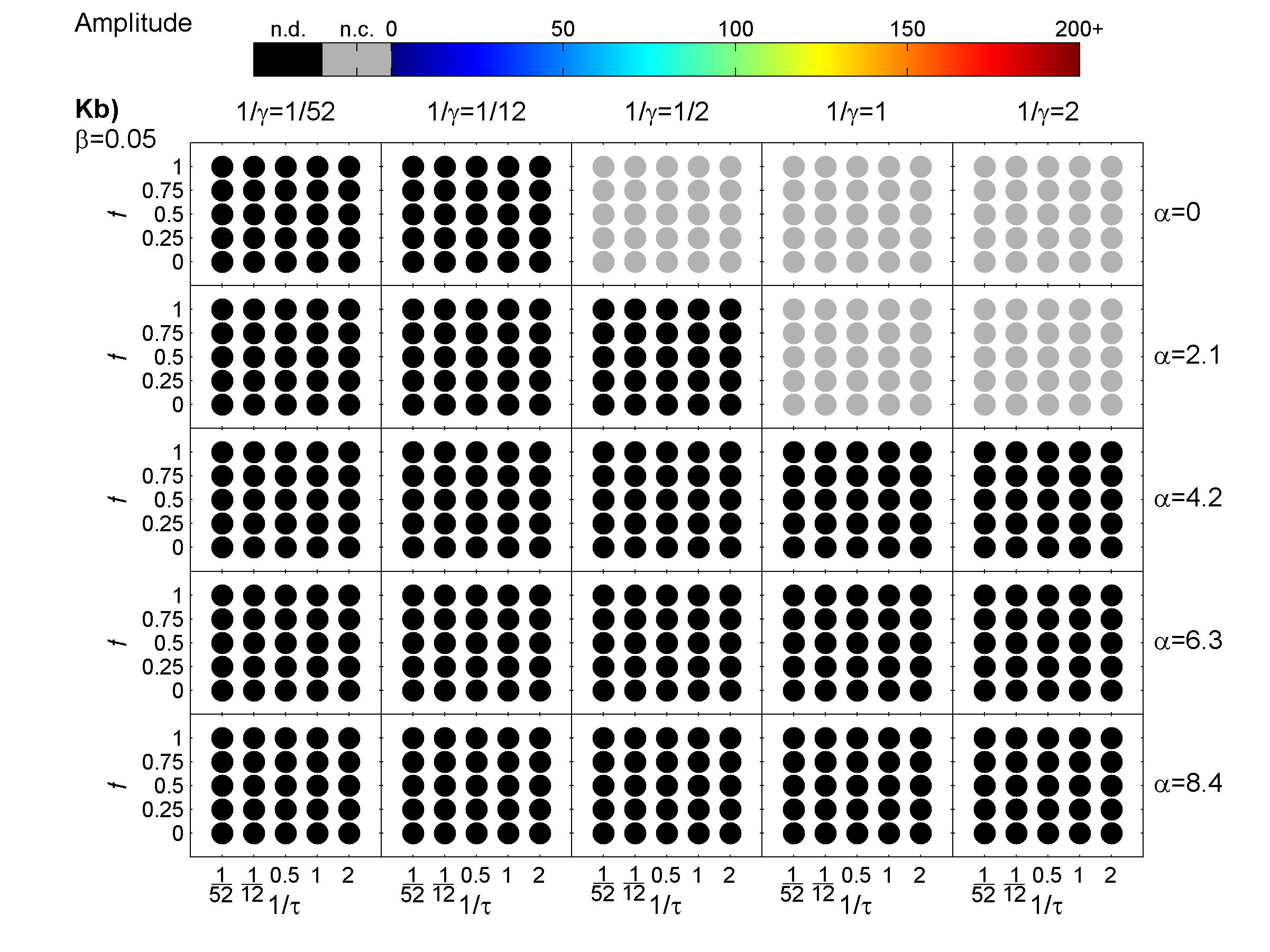

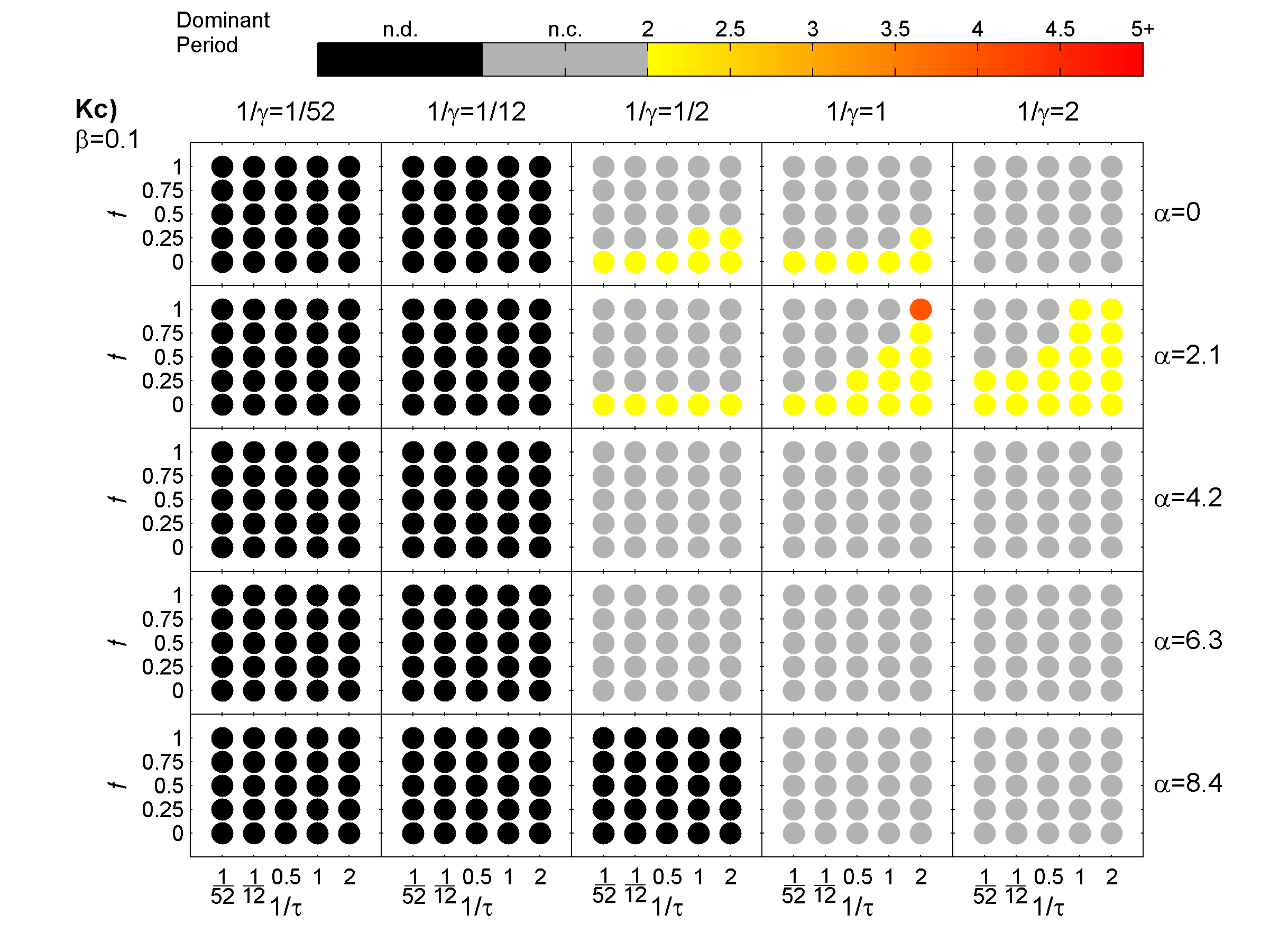

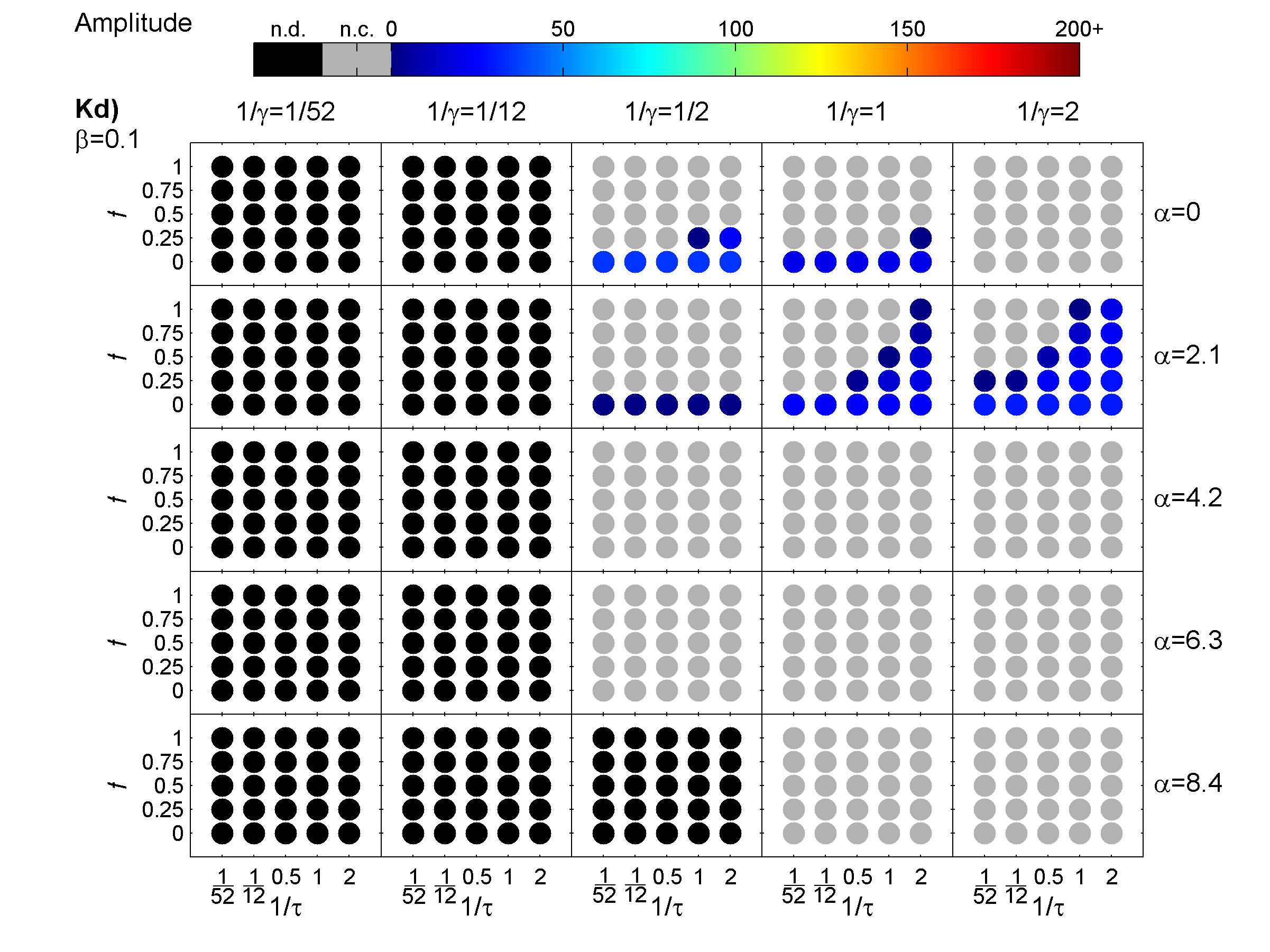

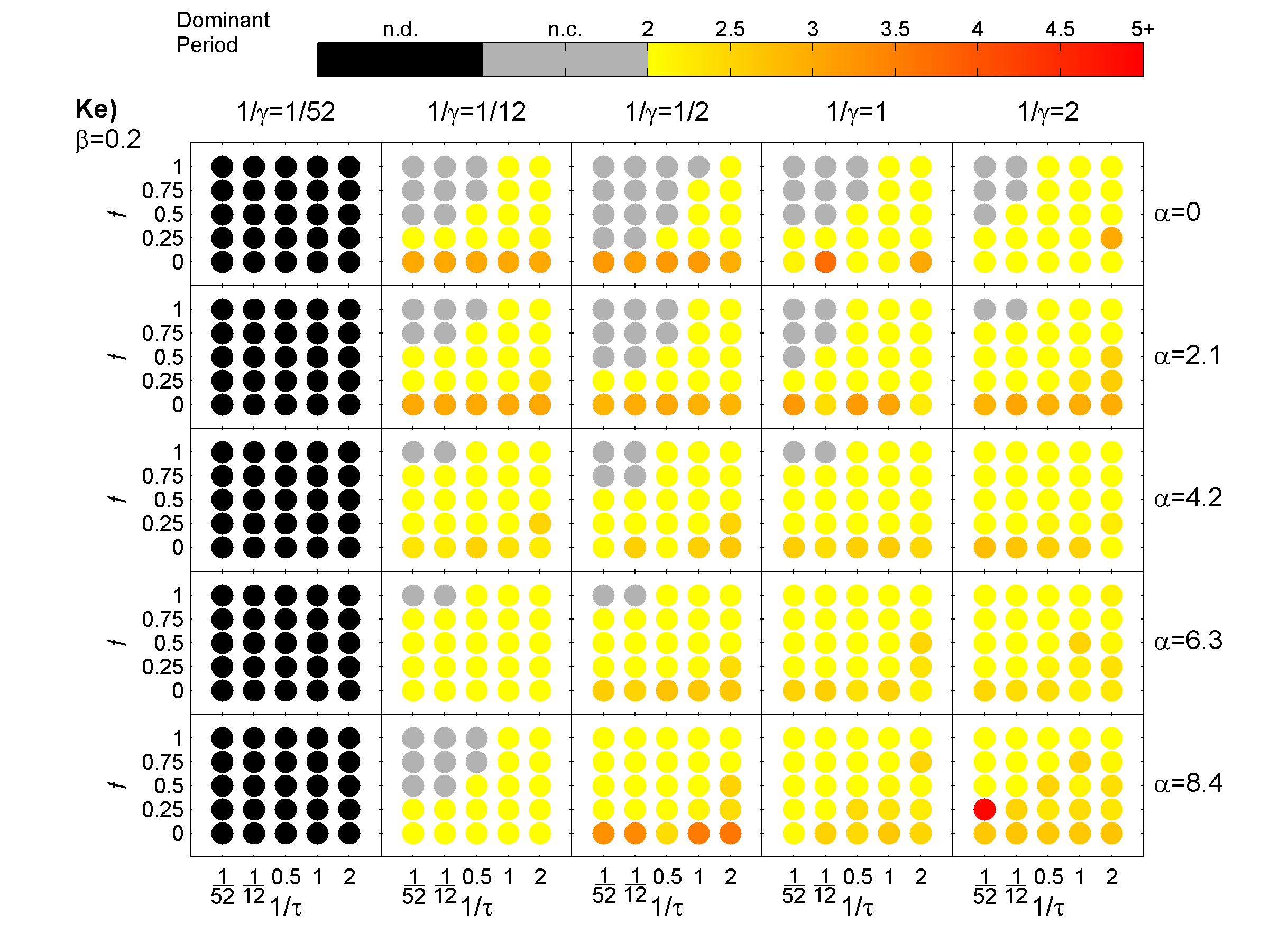

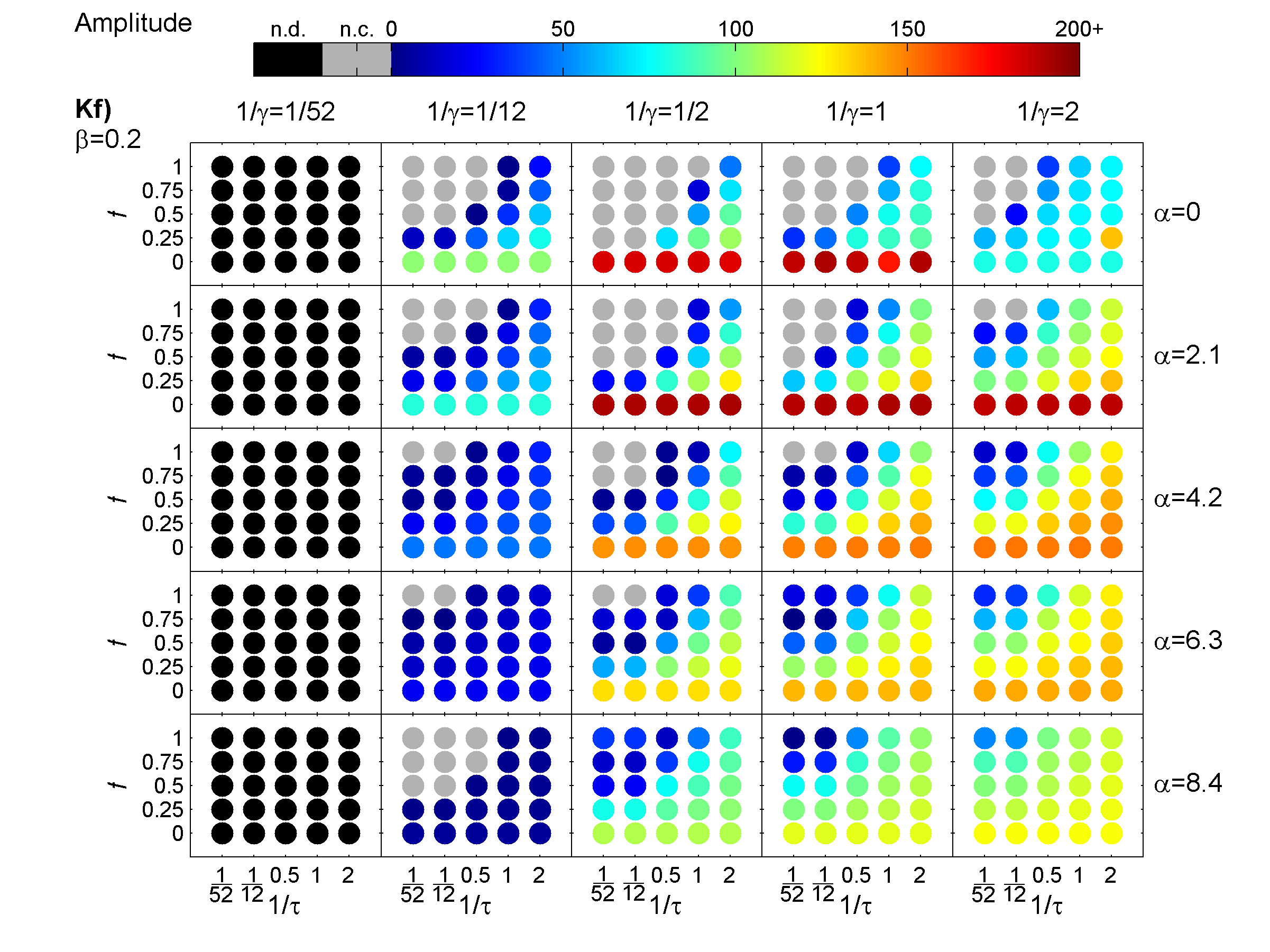

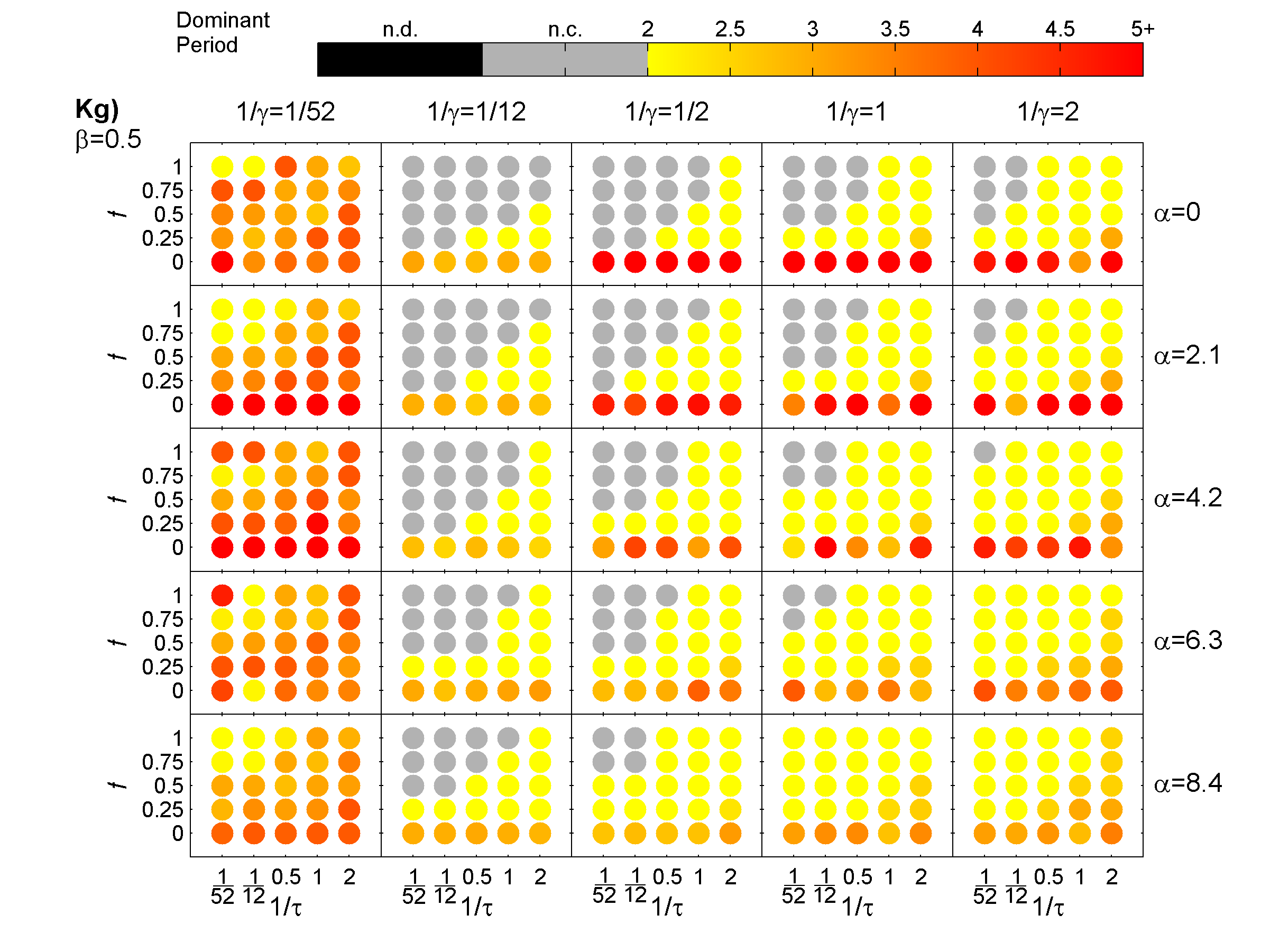

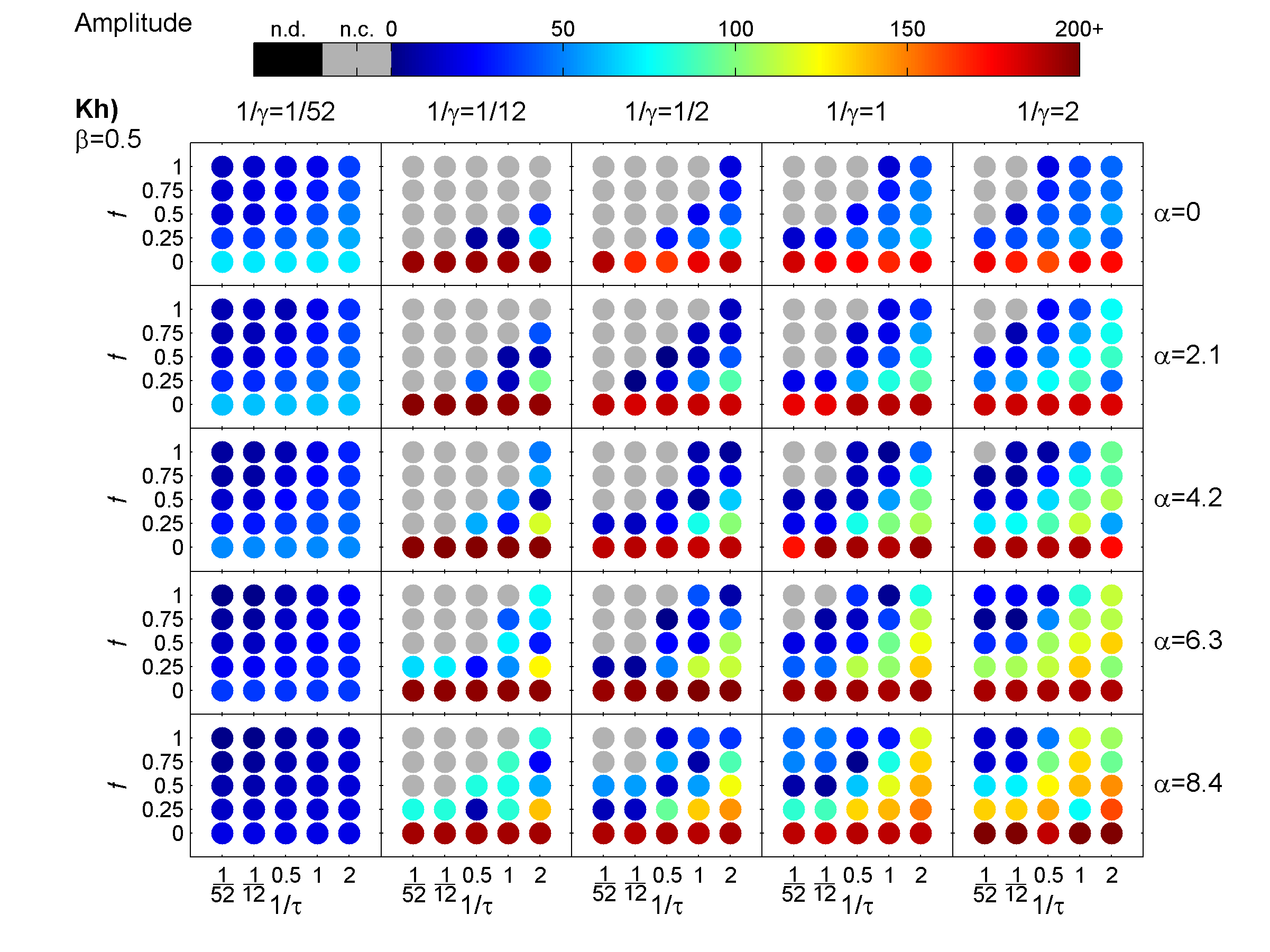

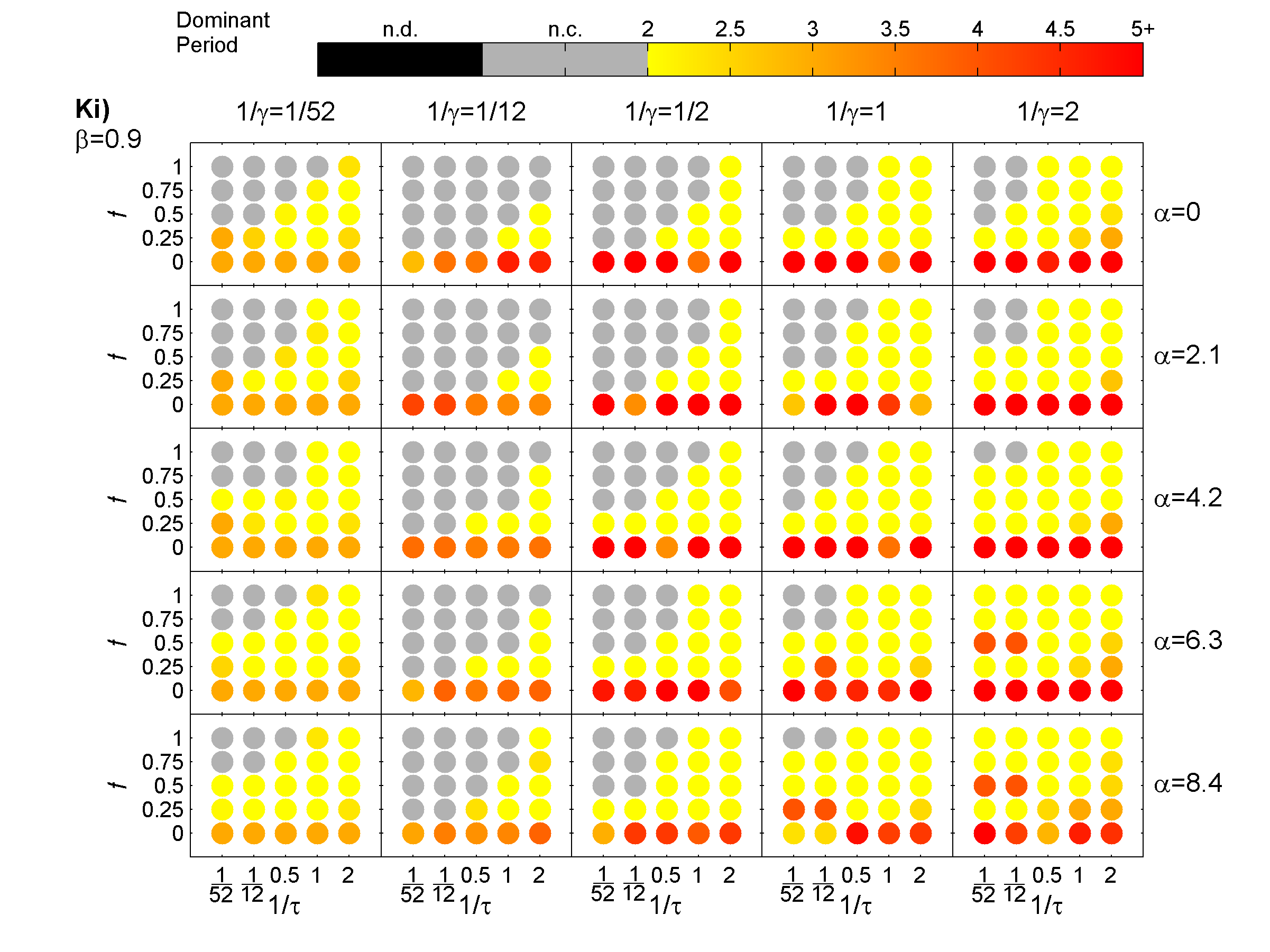

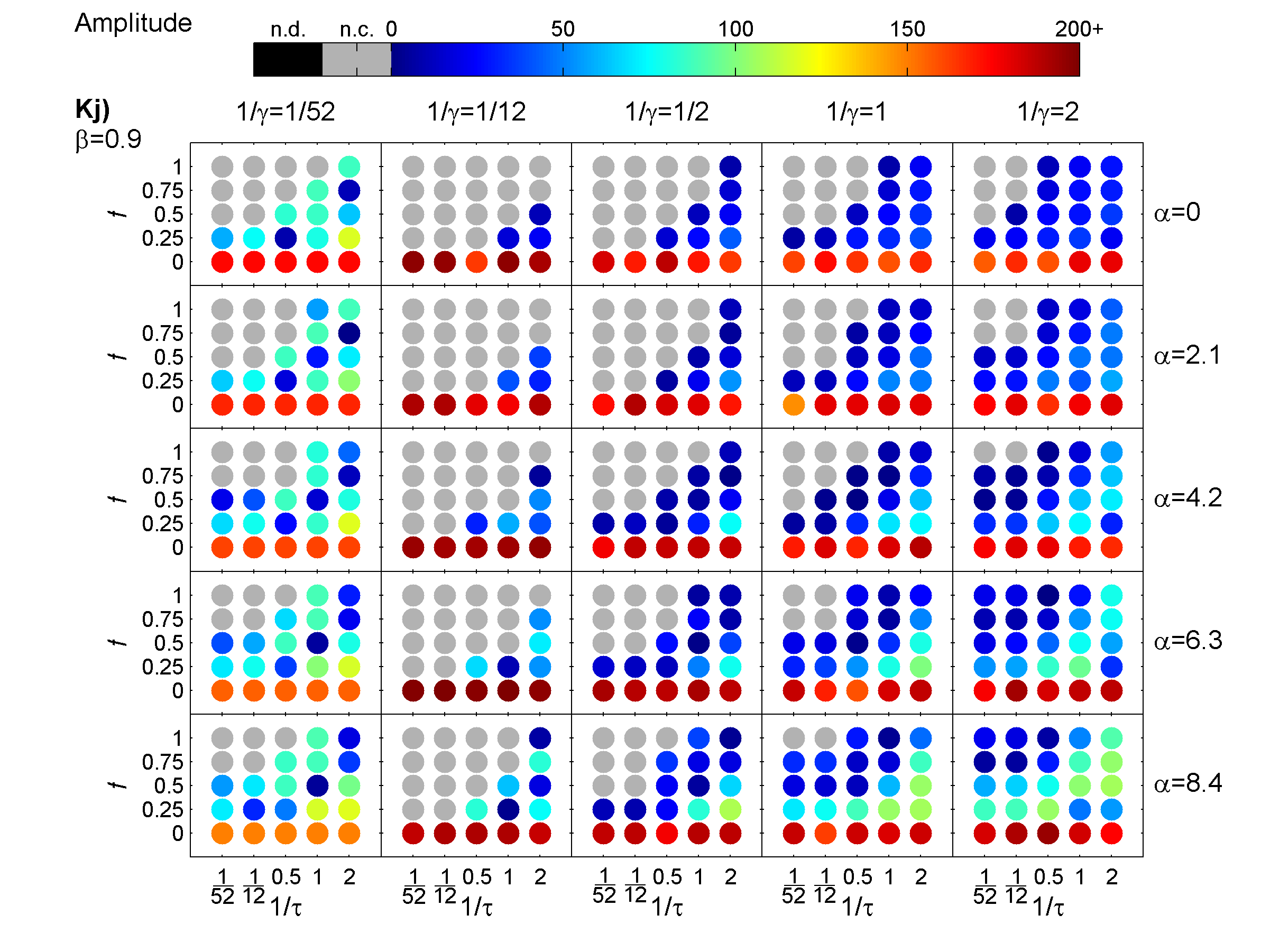
**

**Manor Wood bank voles**

**
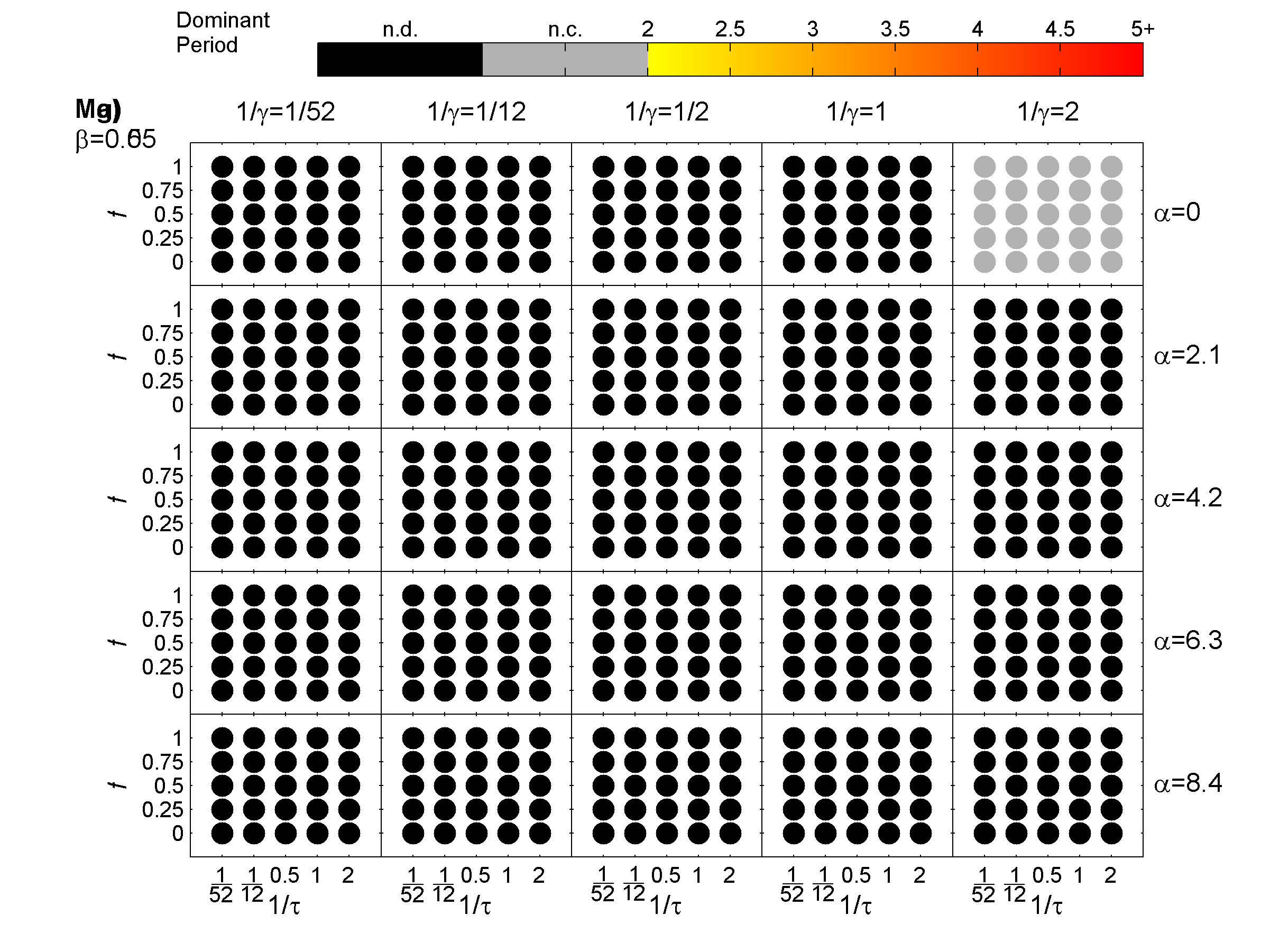

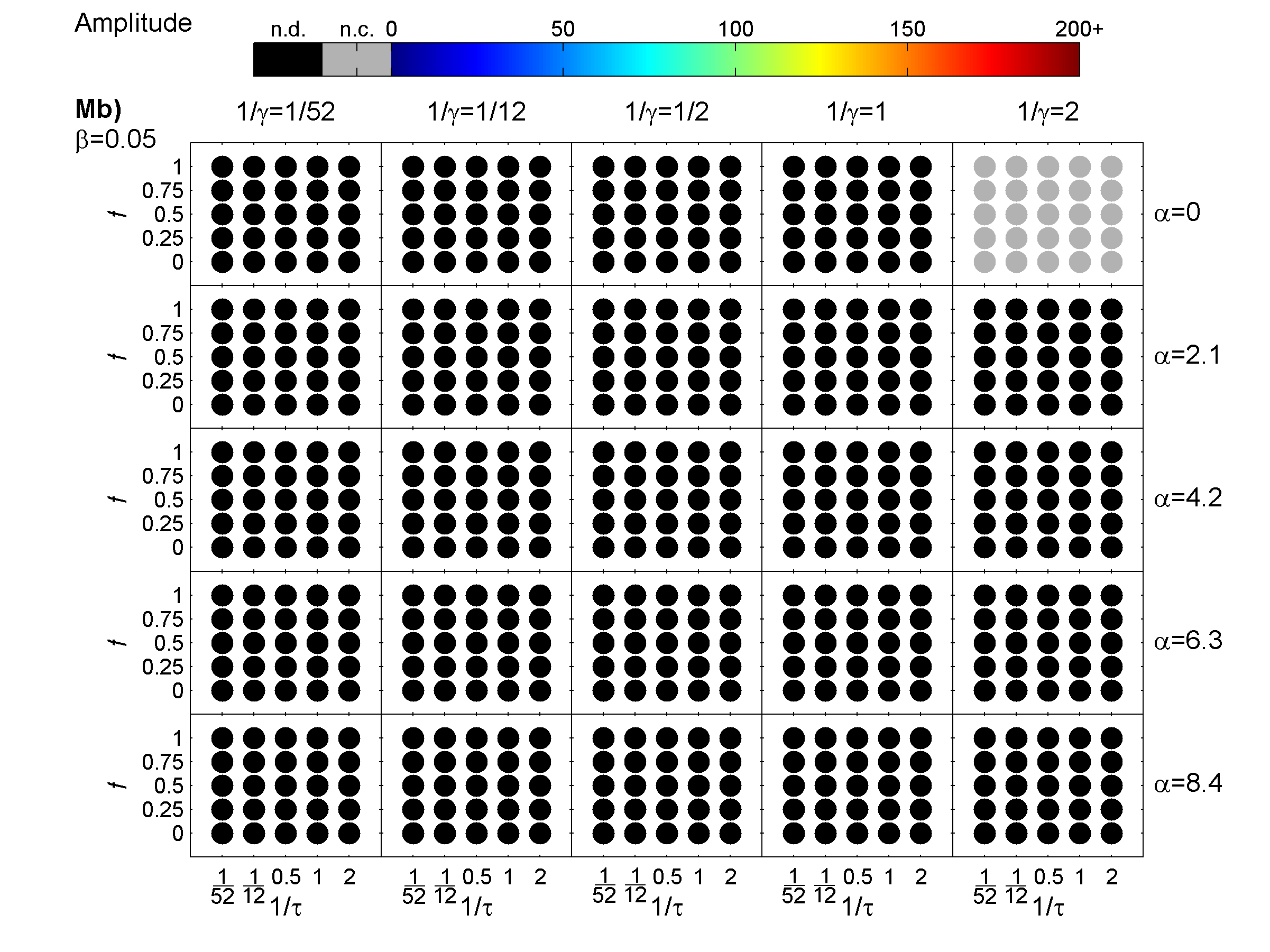

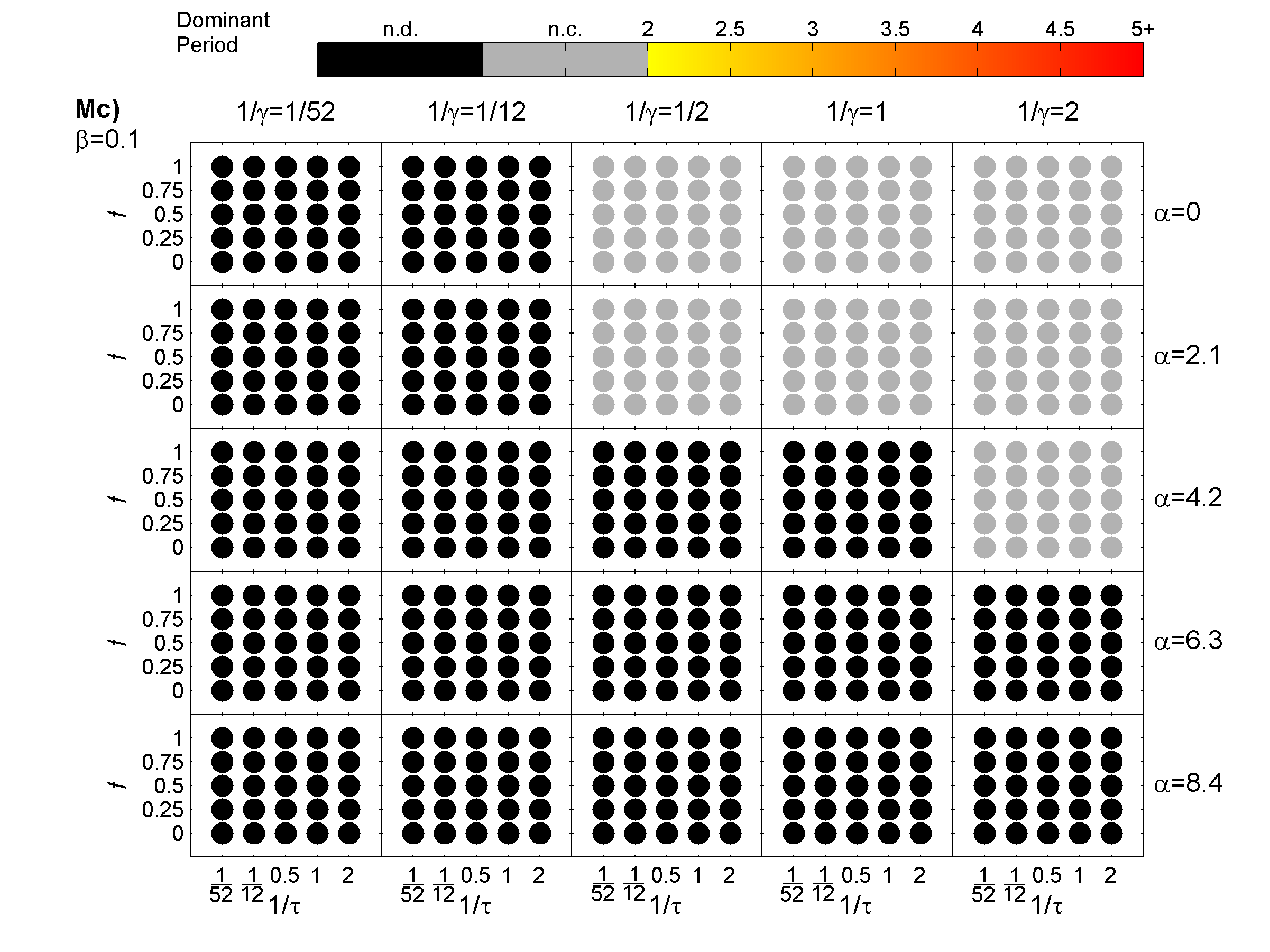

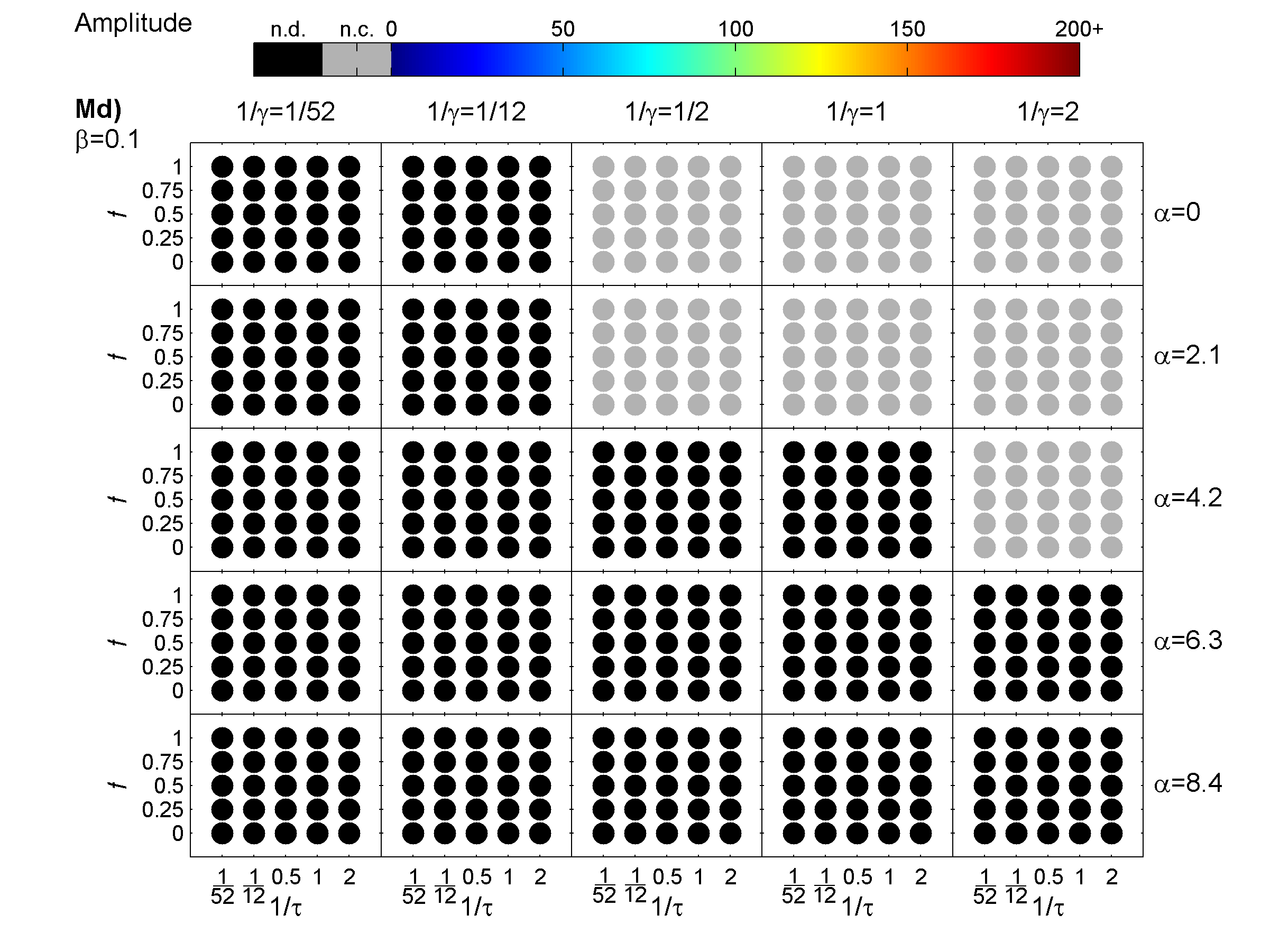

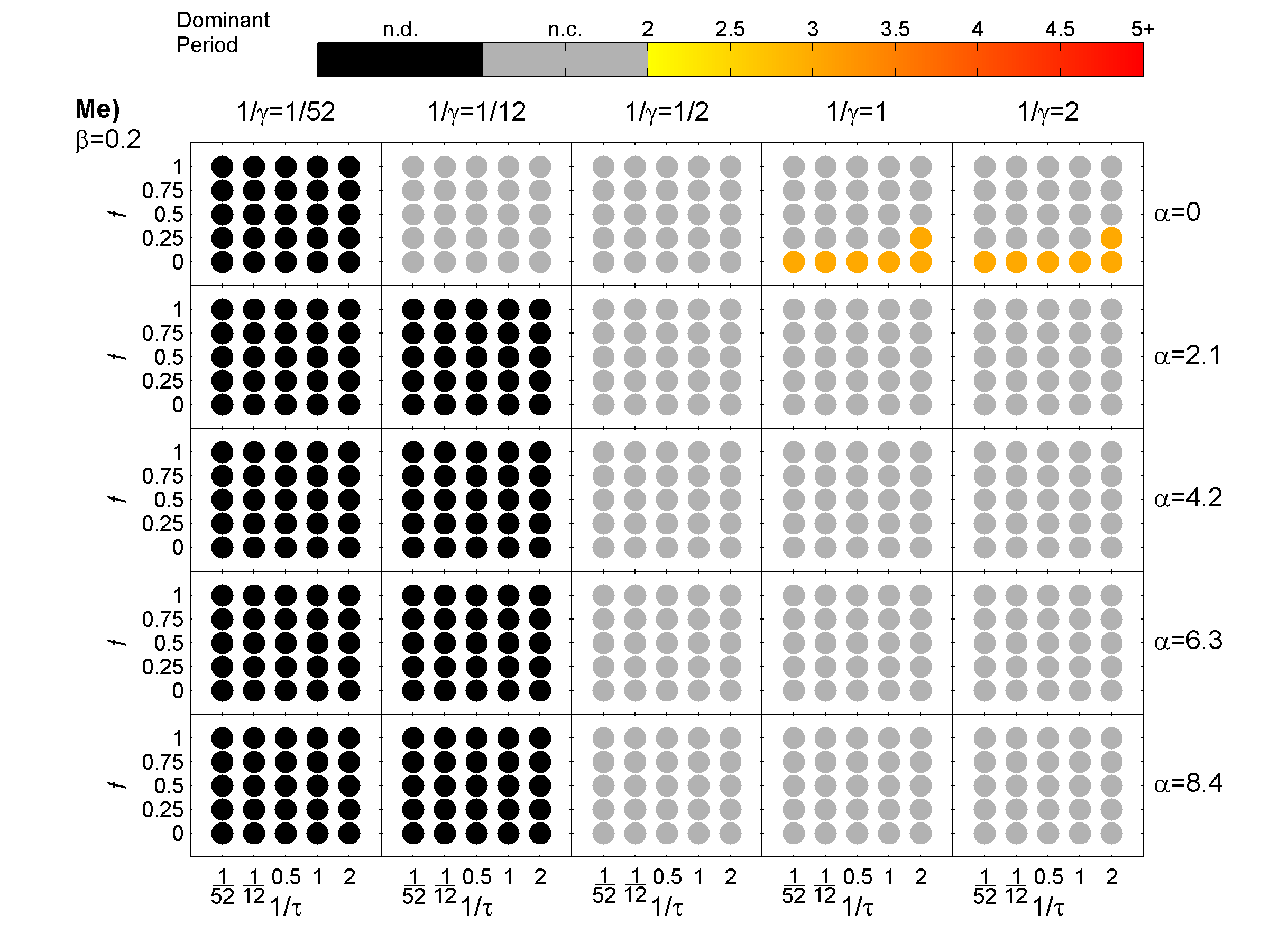

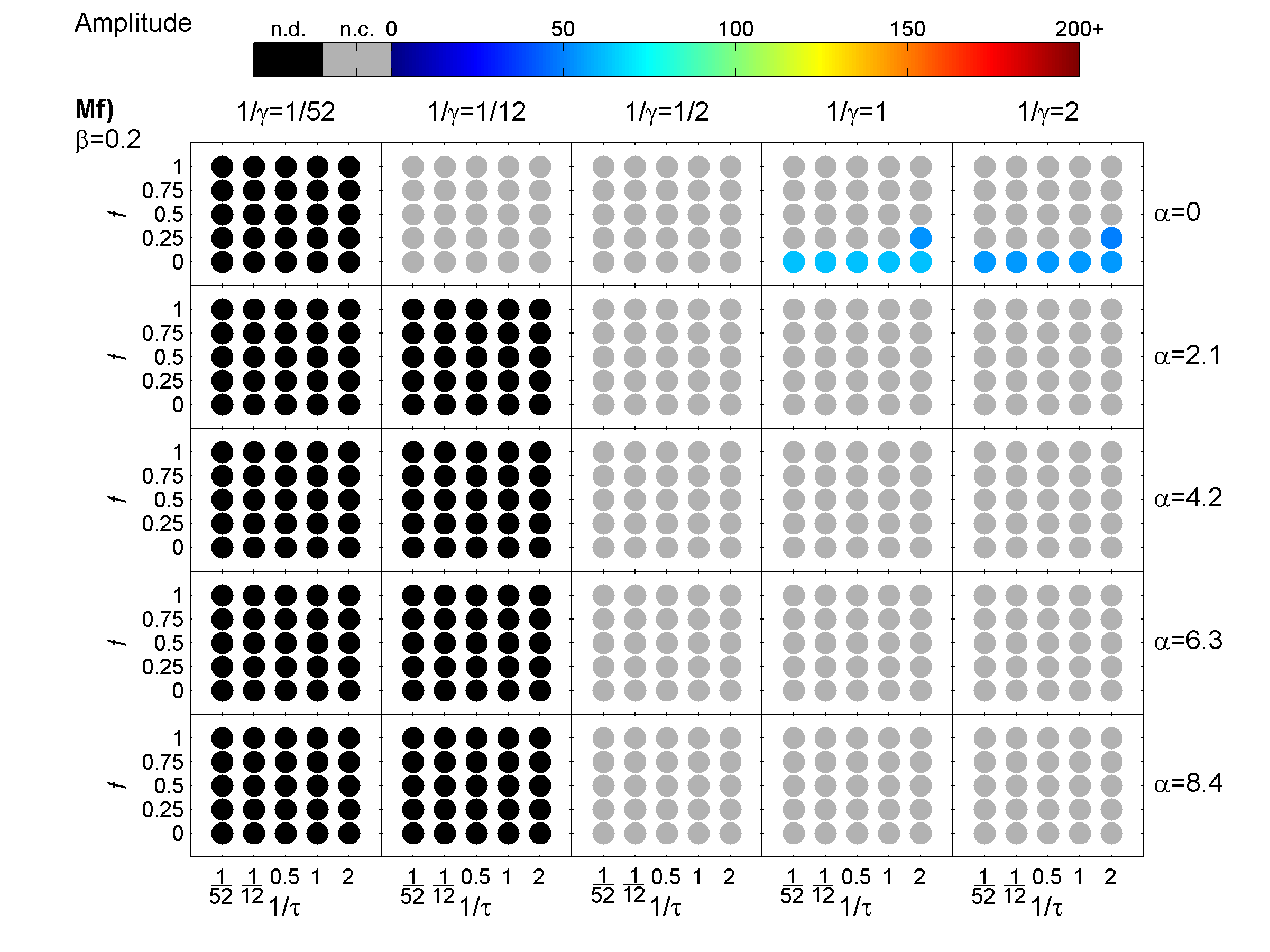

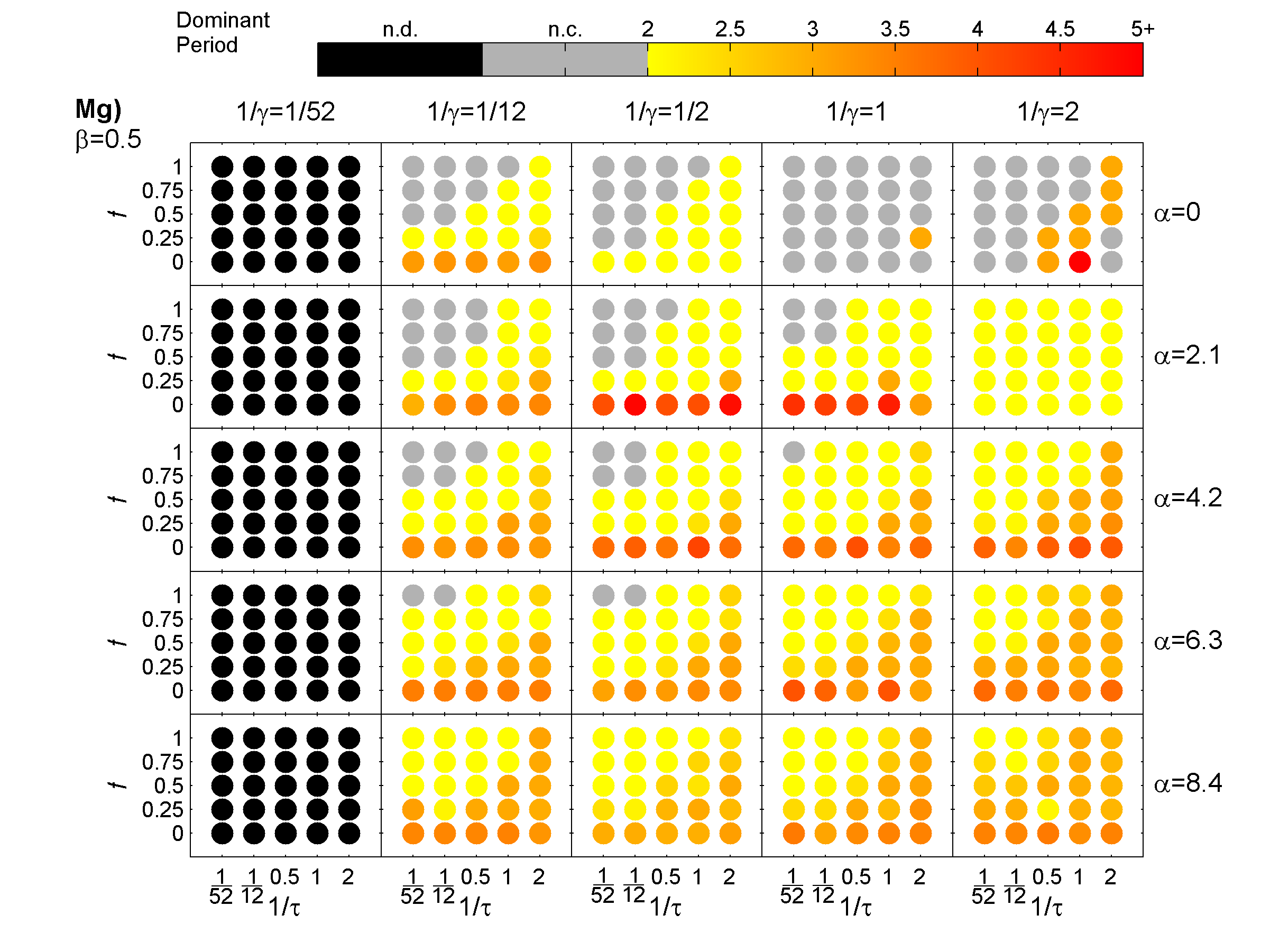

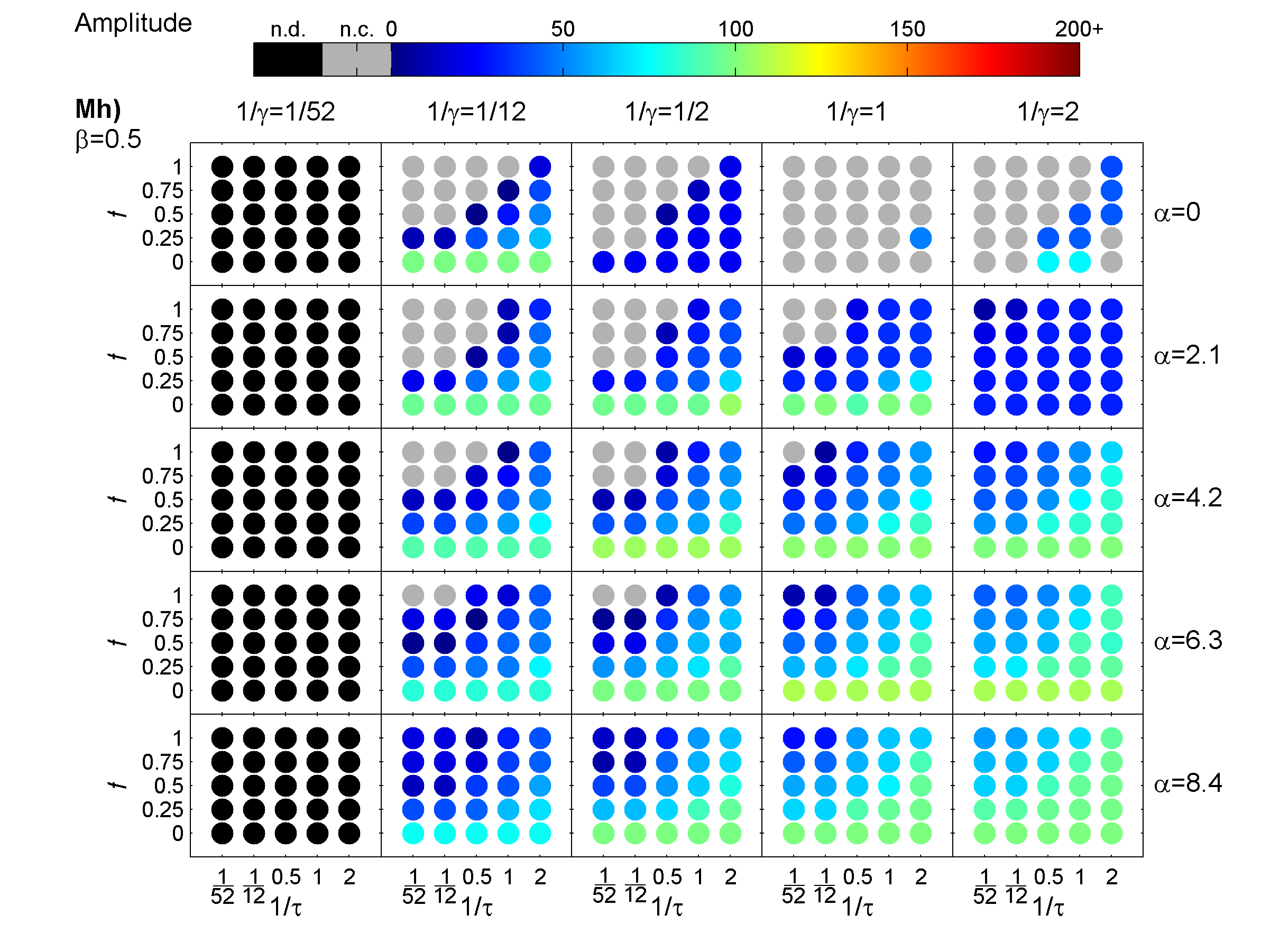

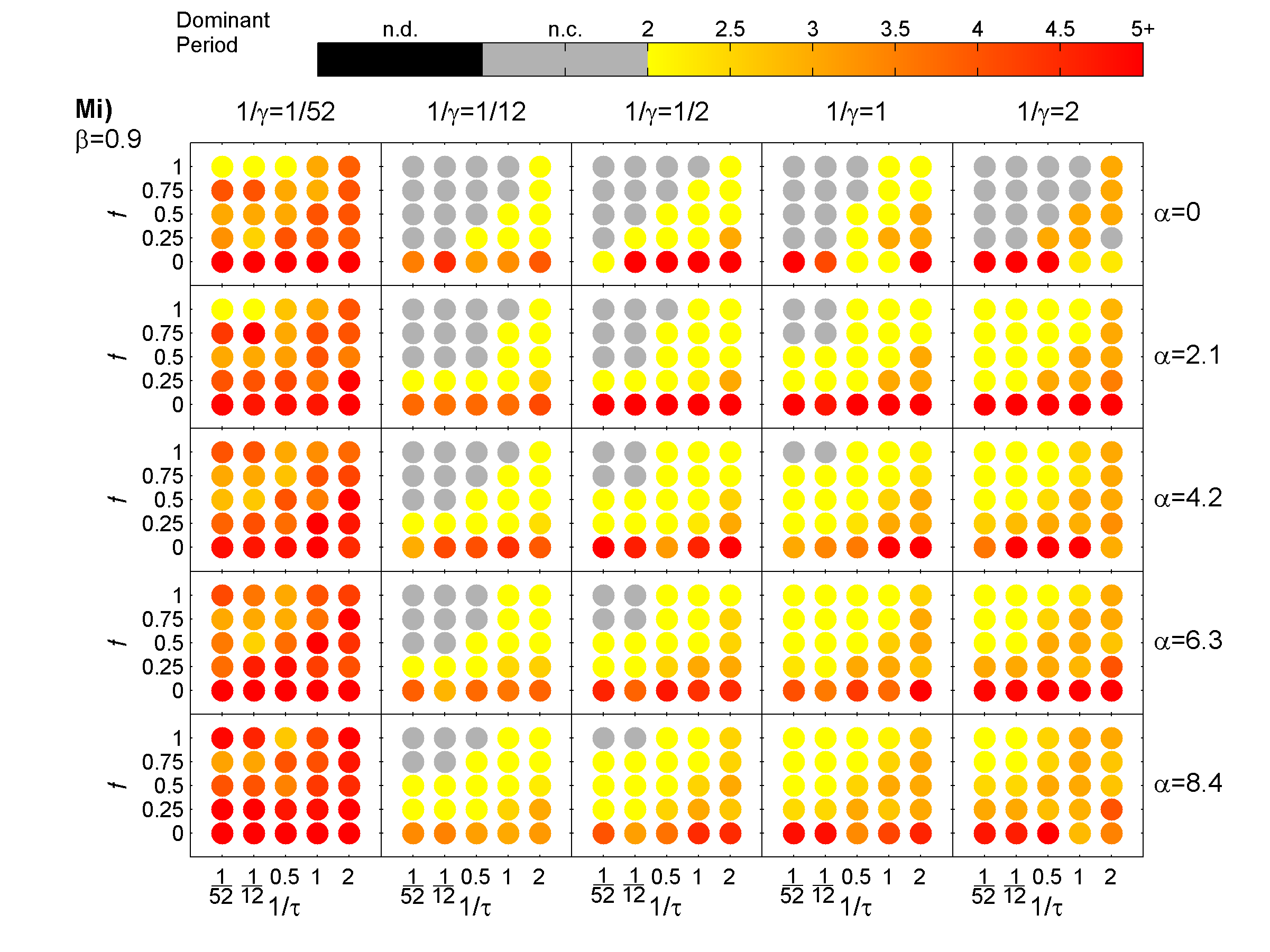

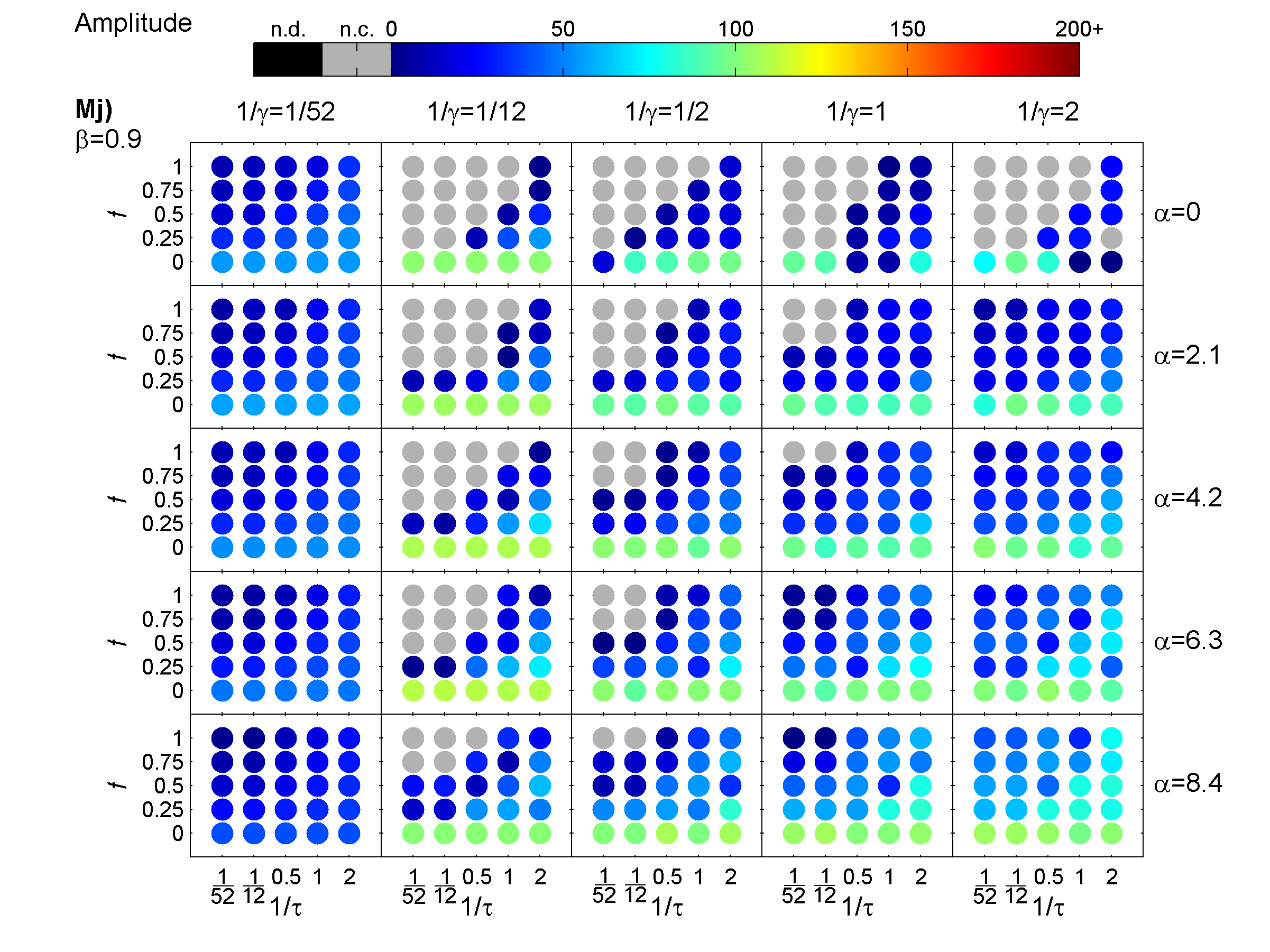
**

**French common voles**

**
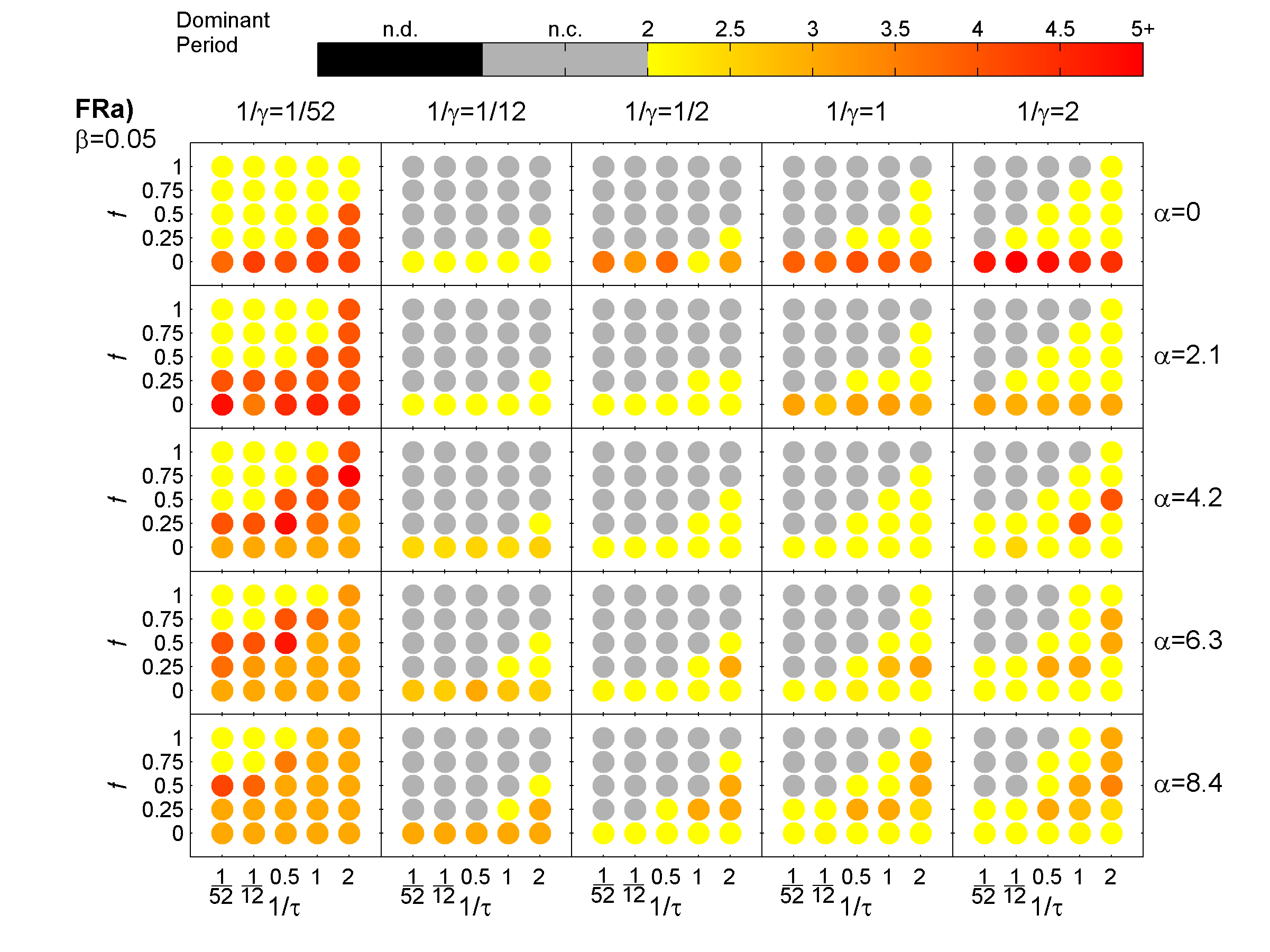

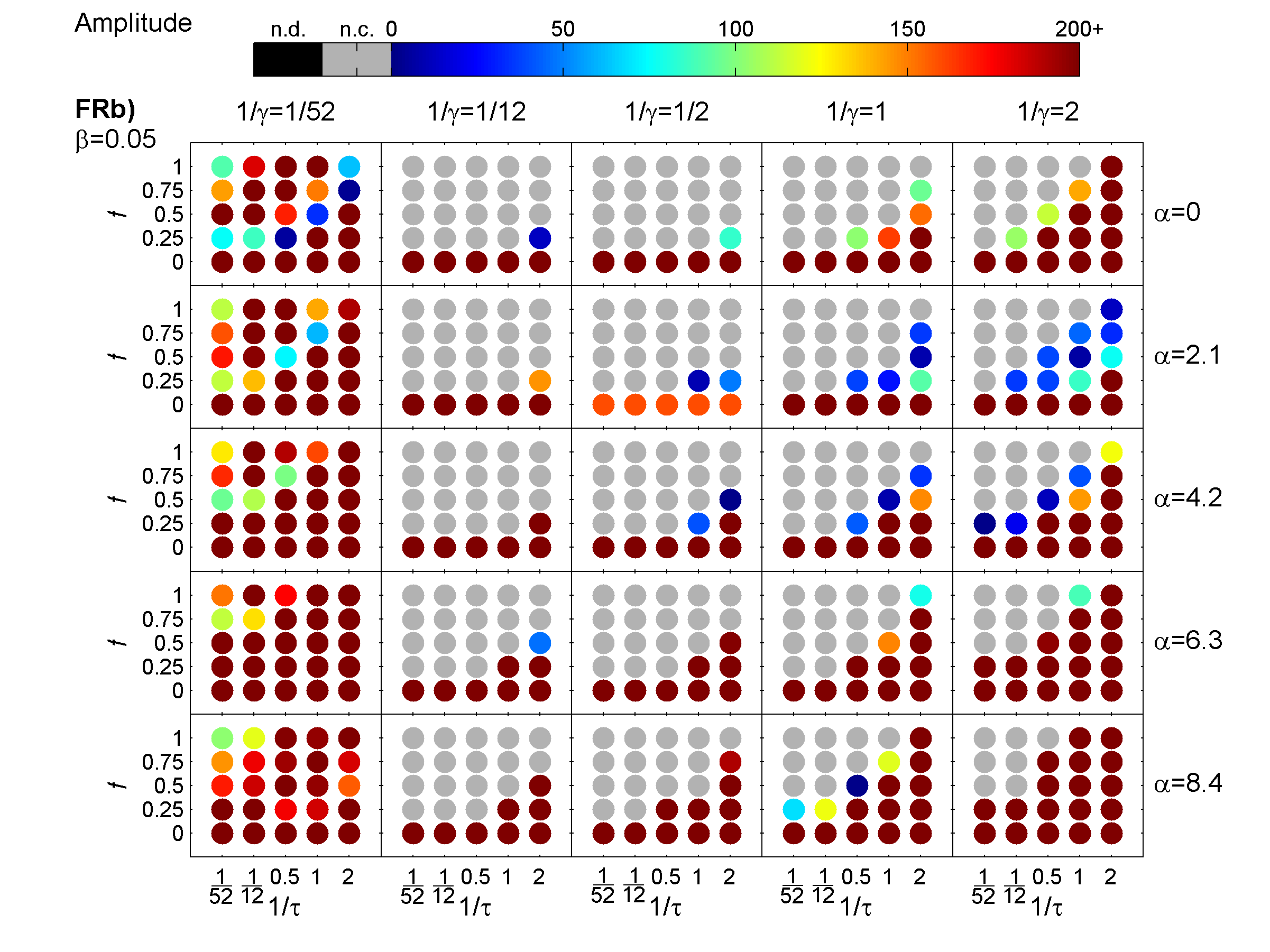

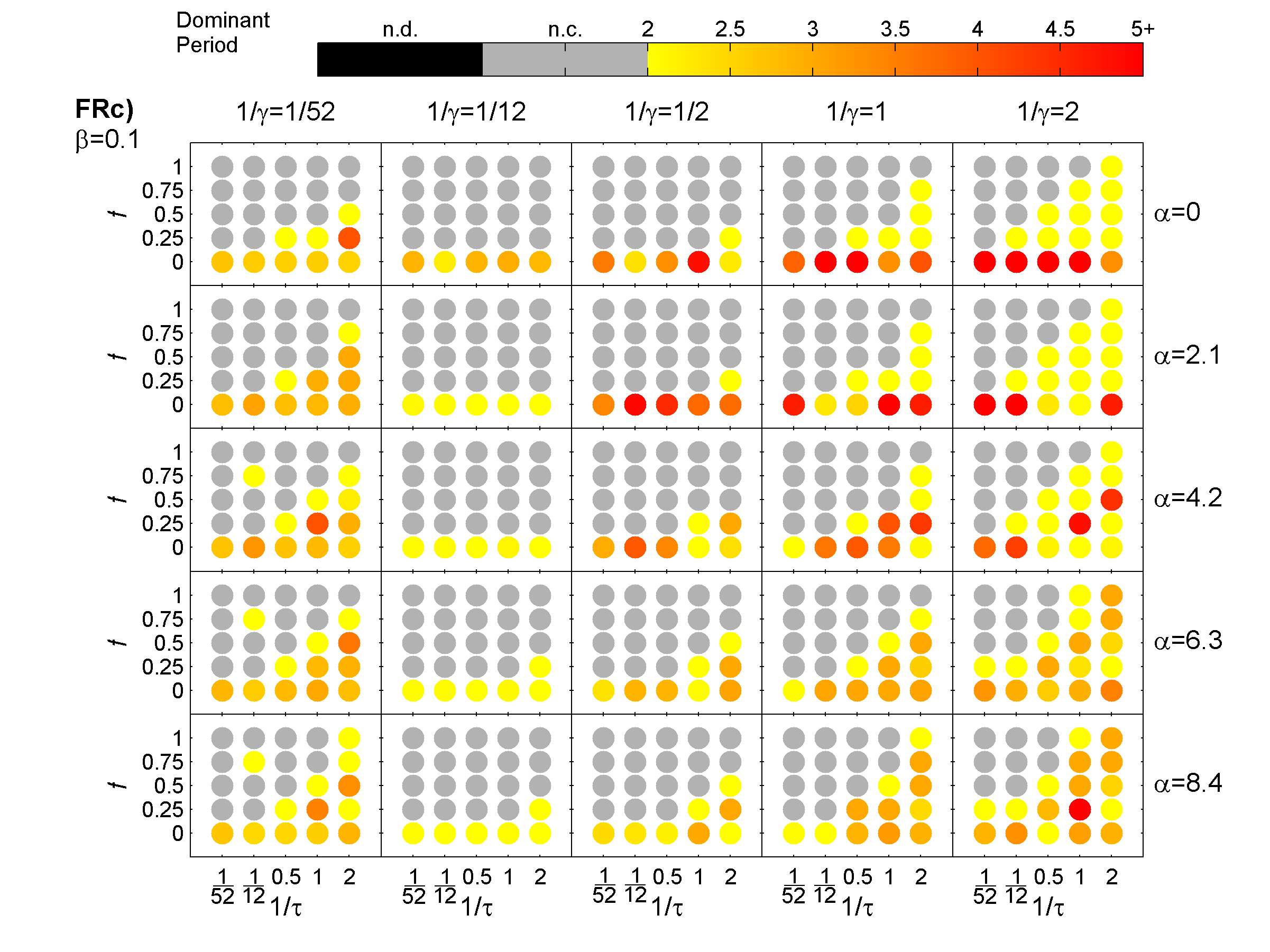

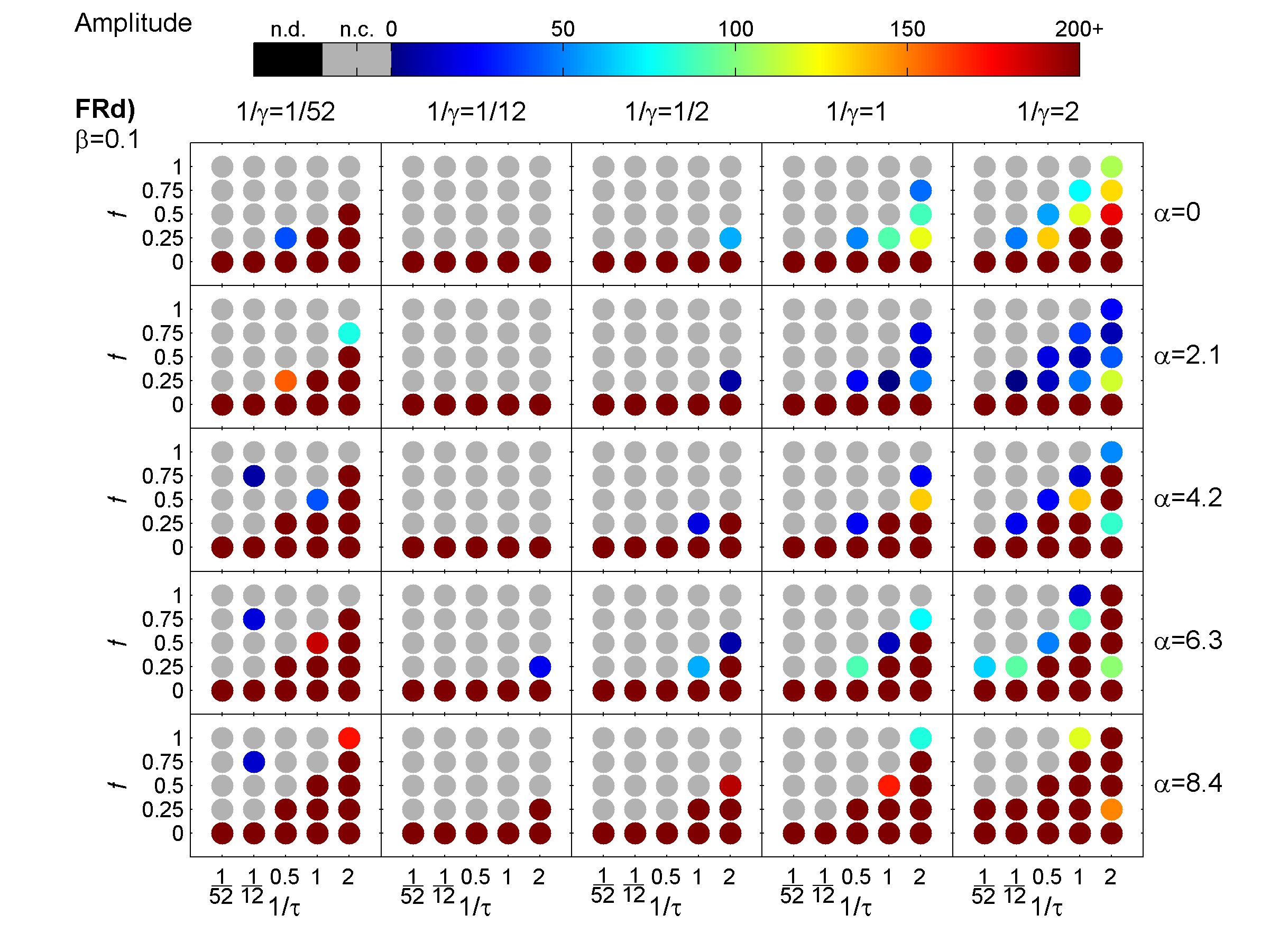

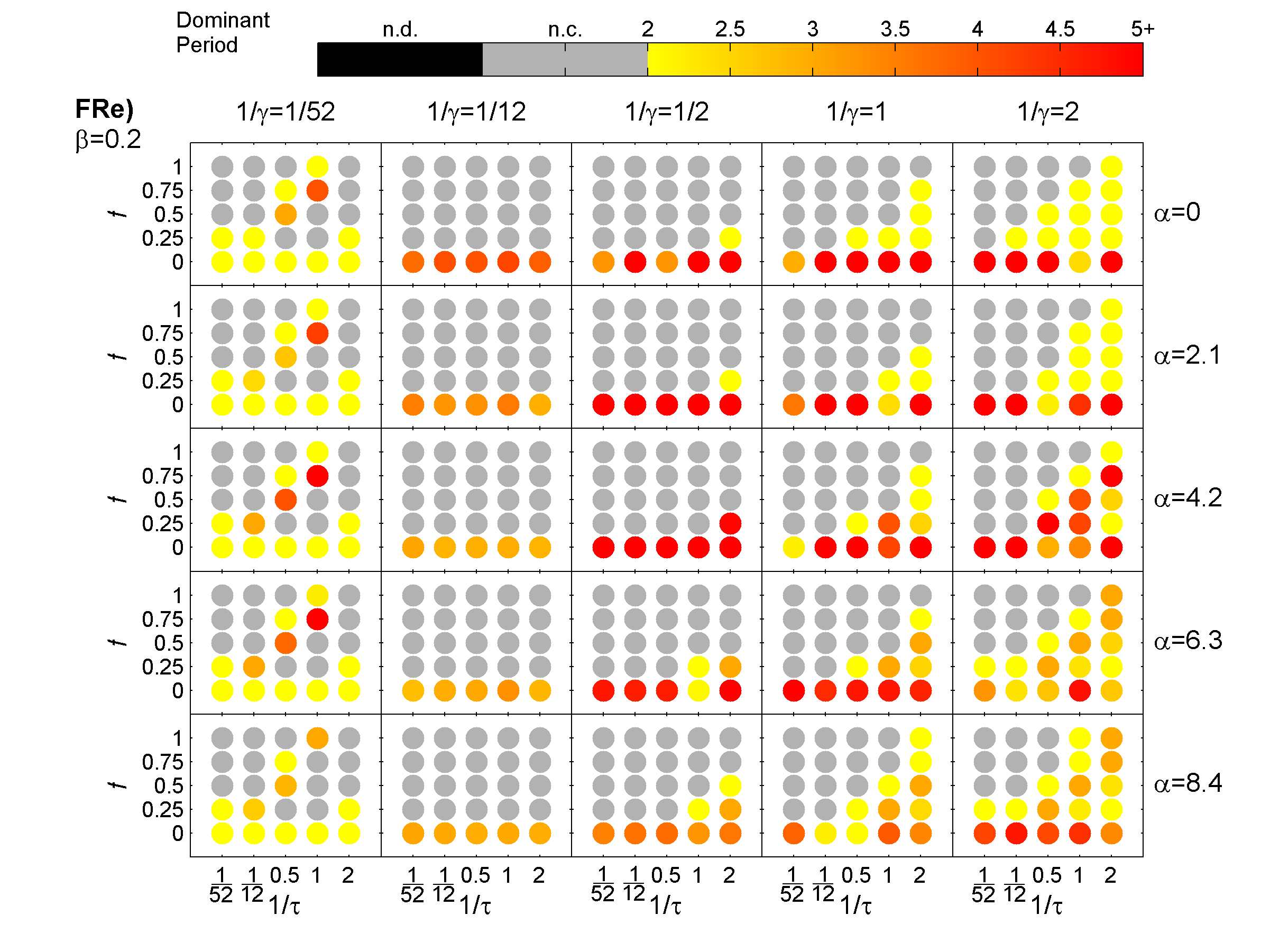

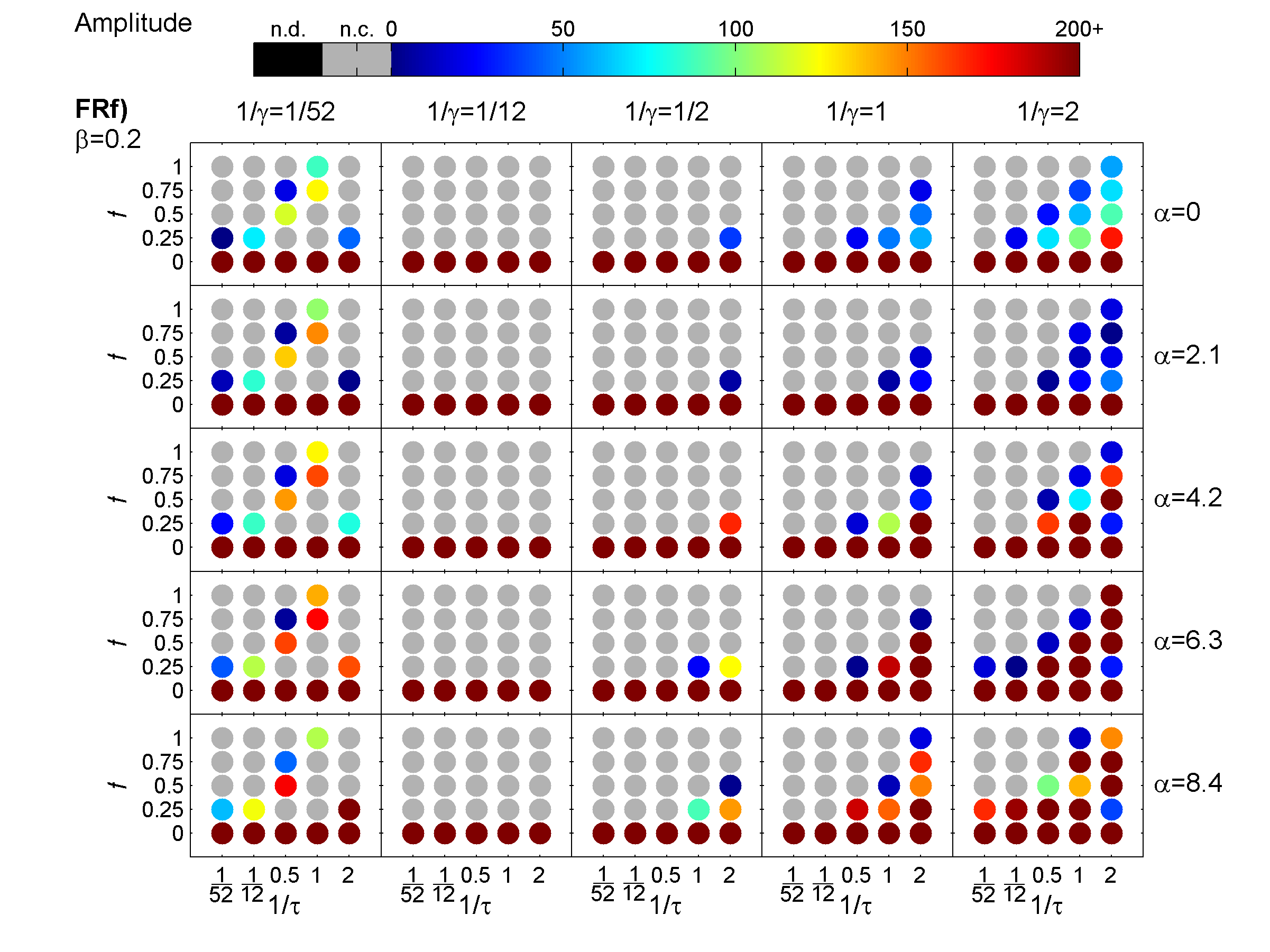

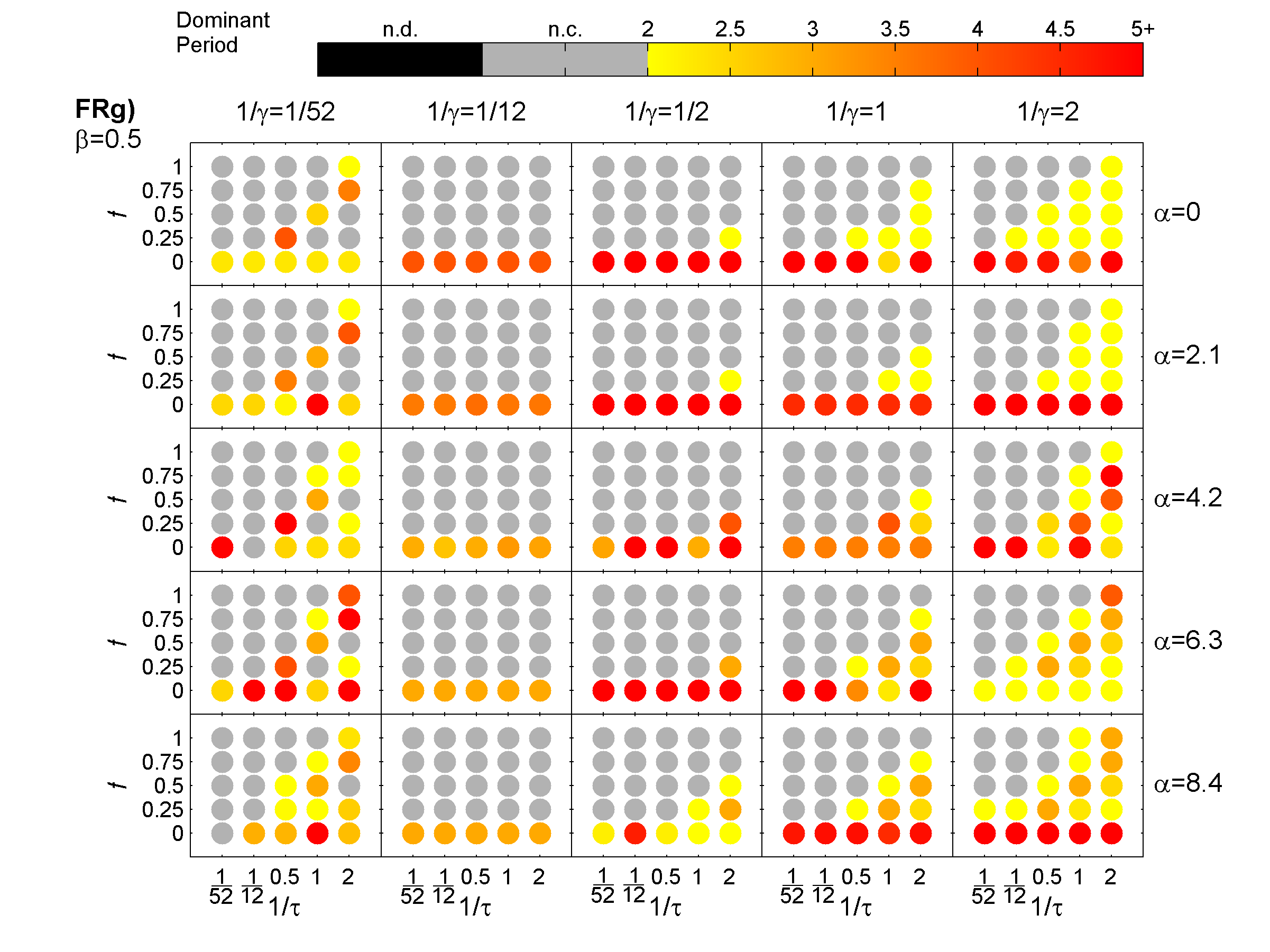

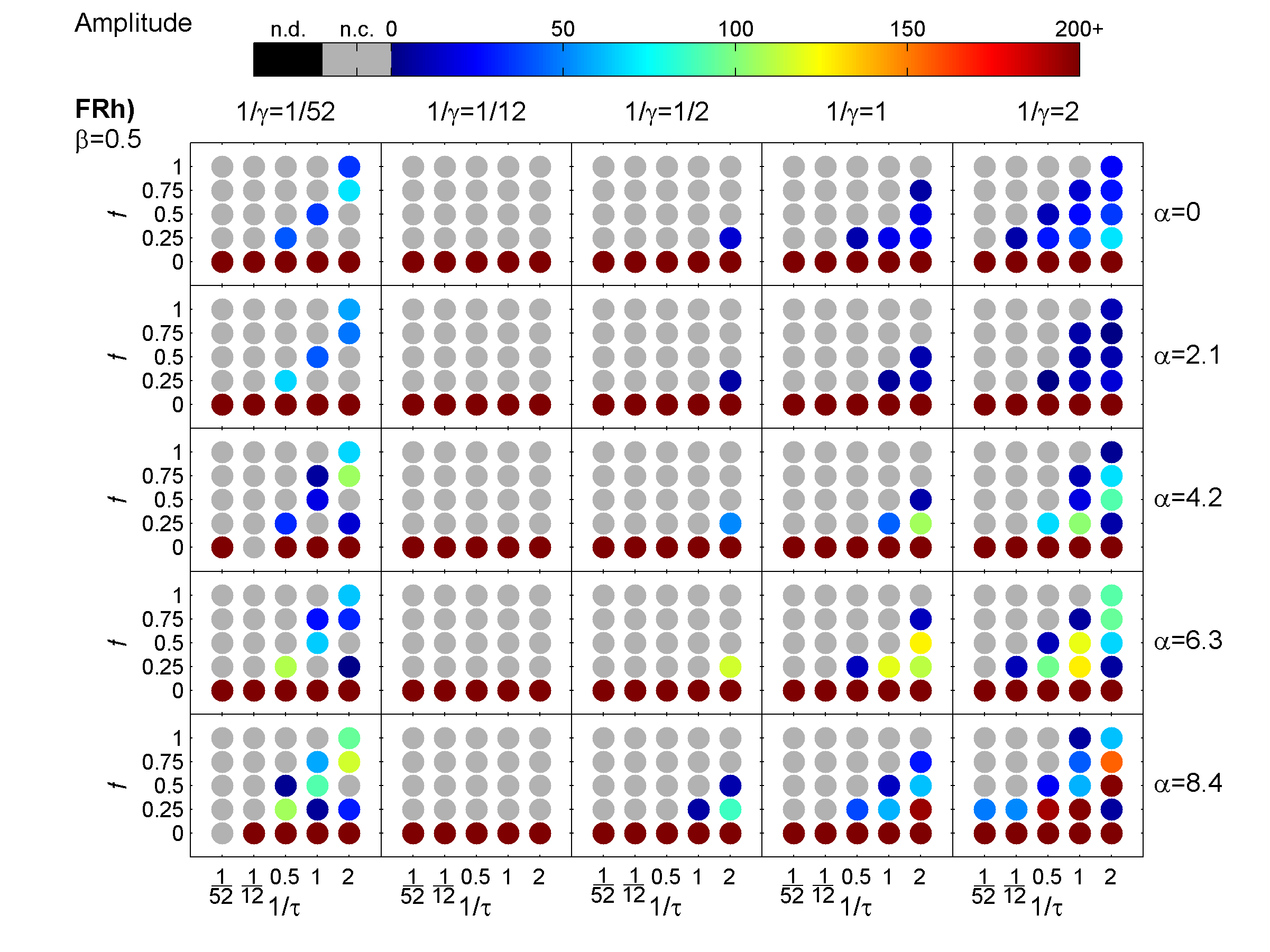

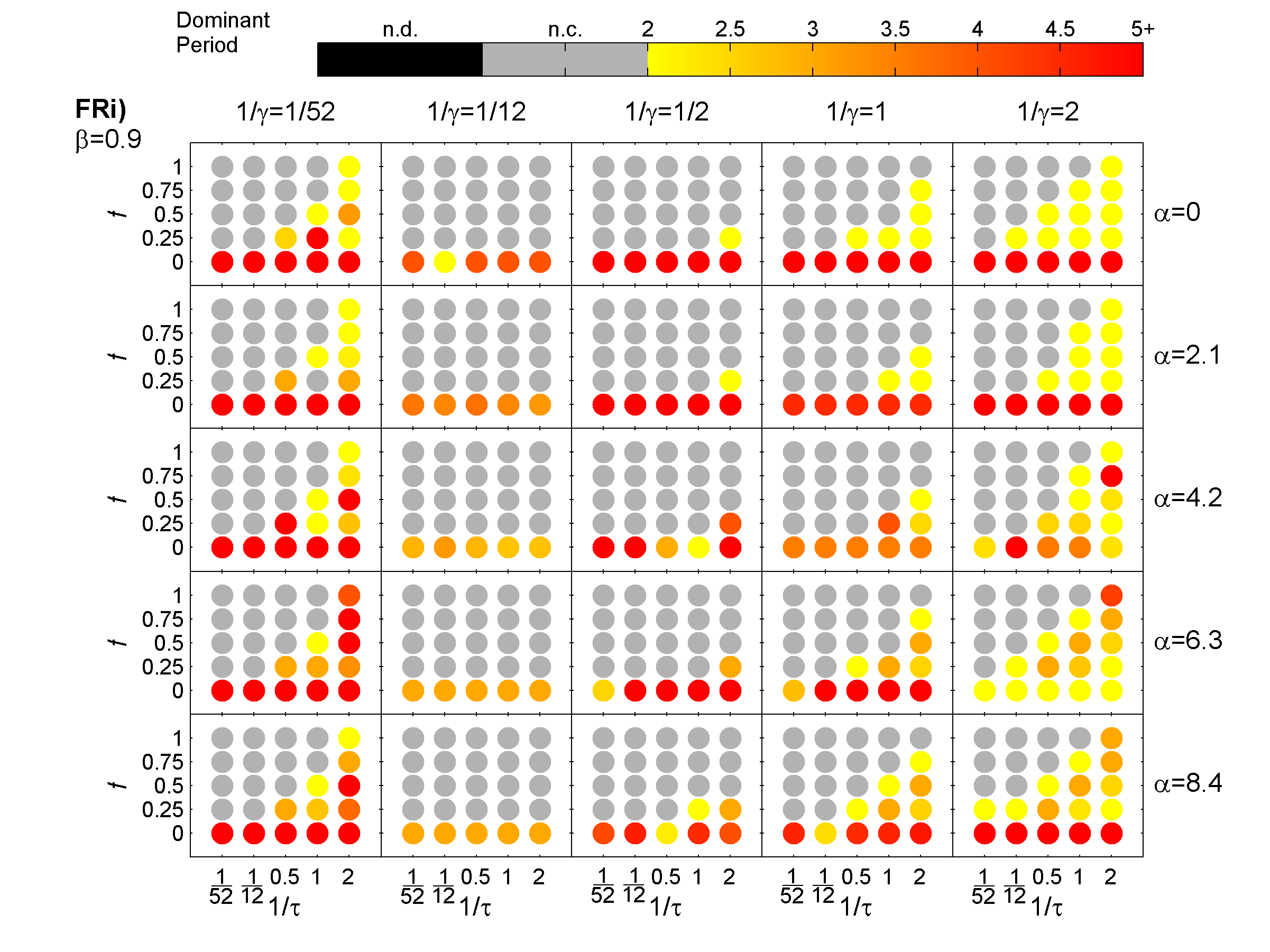

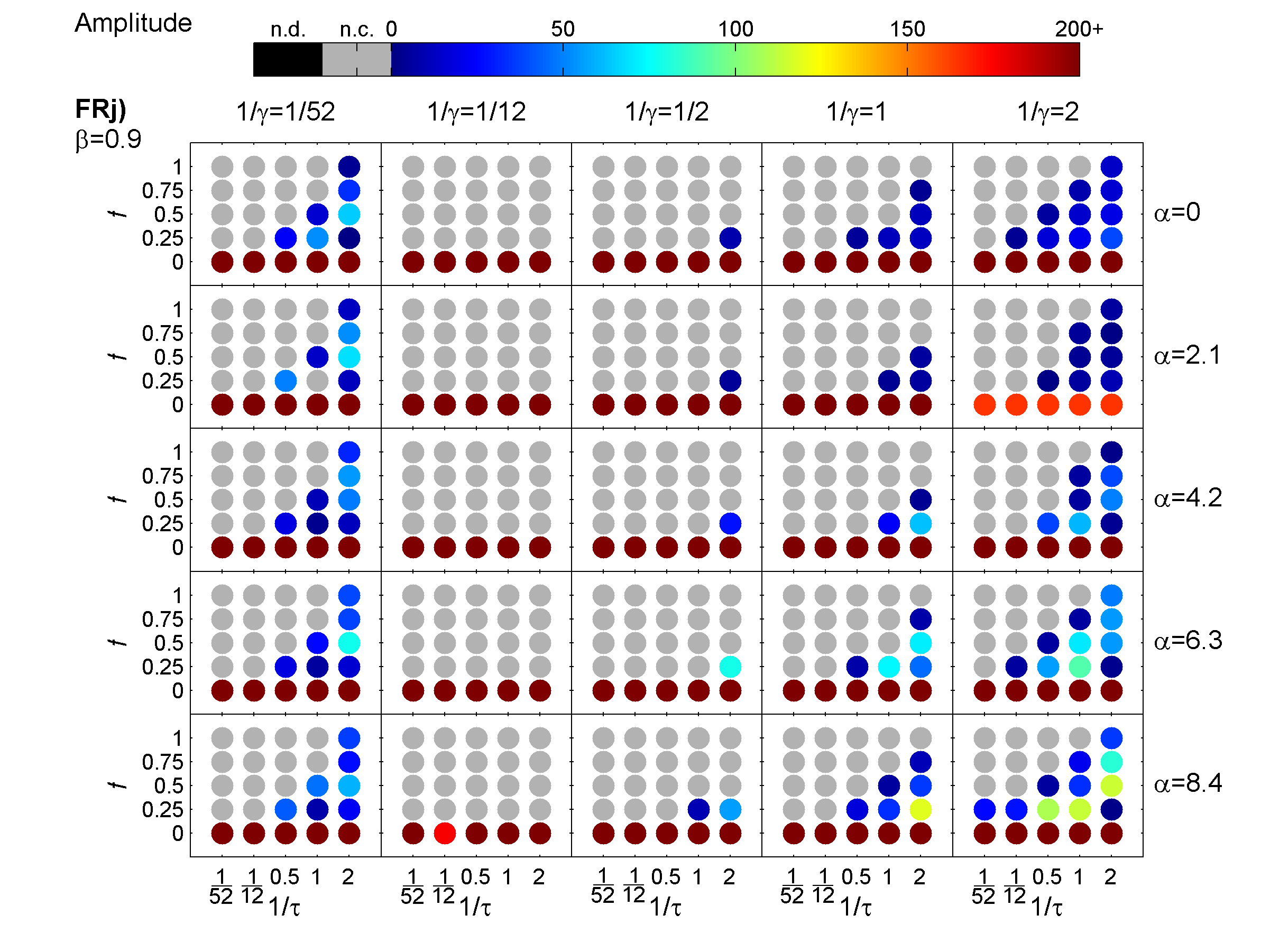
**

**Fennoscandian field voles**

**
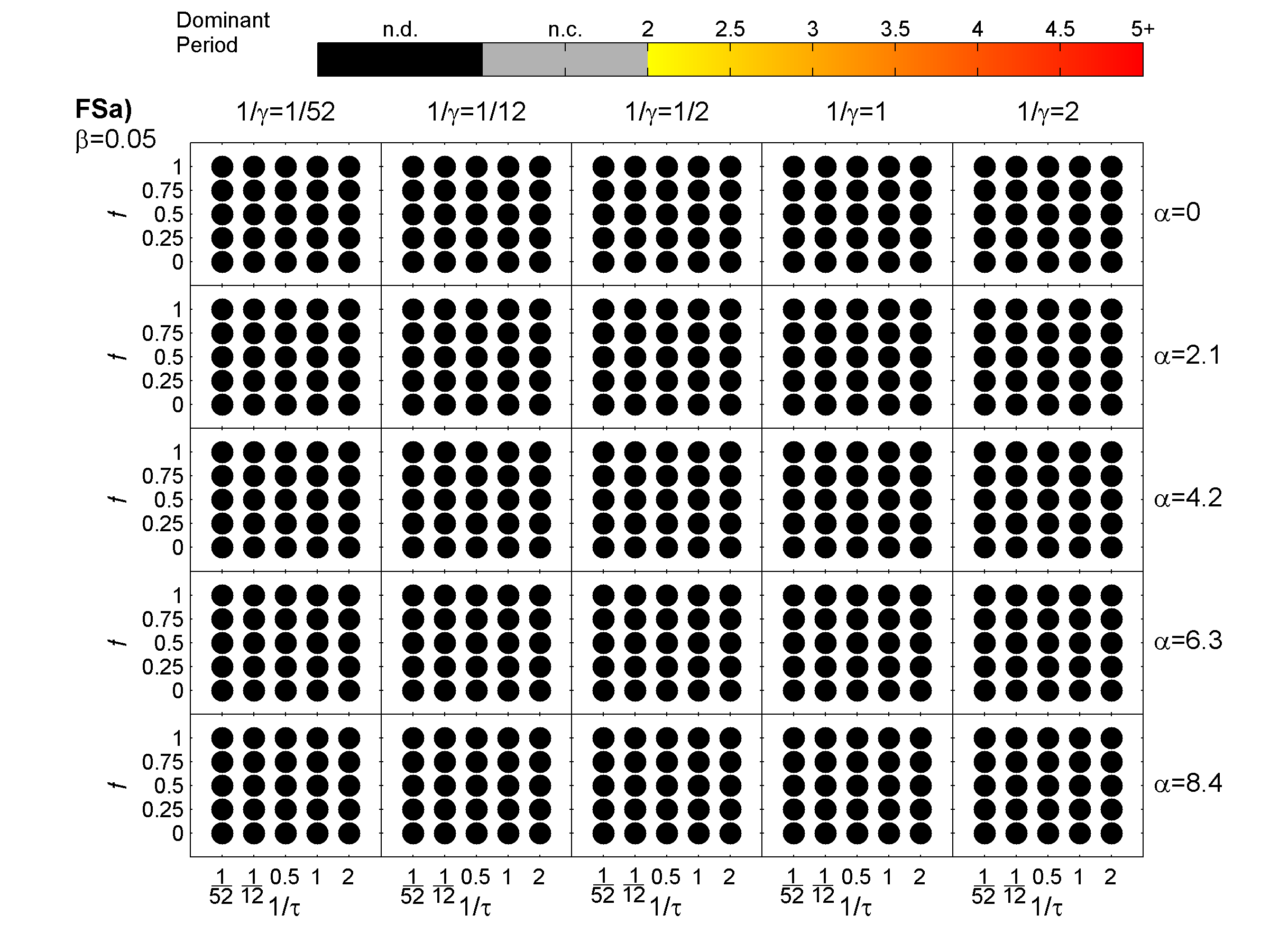

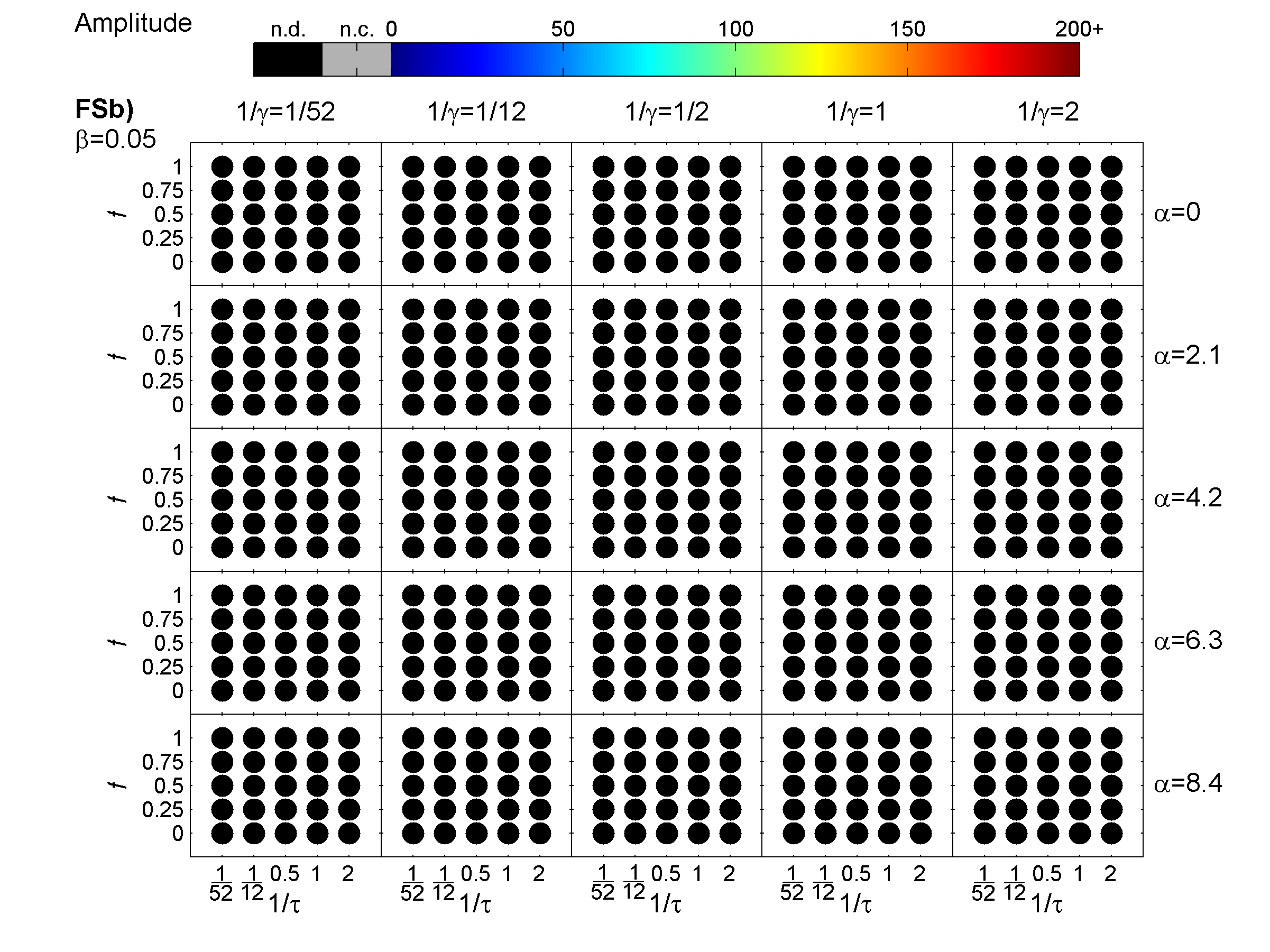

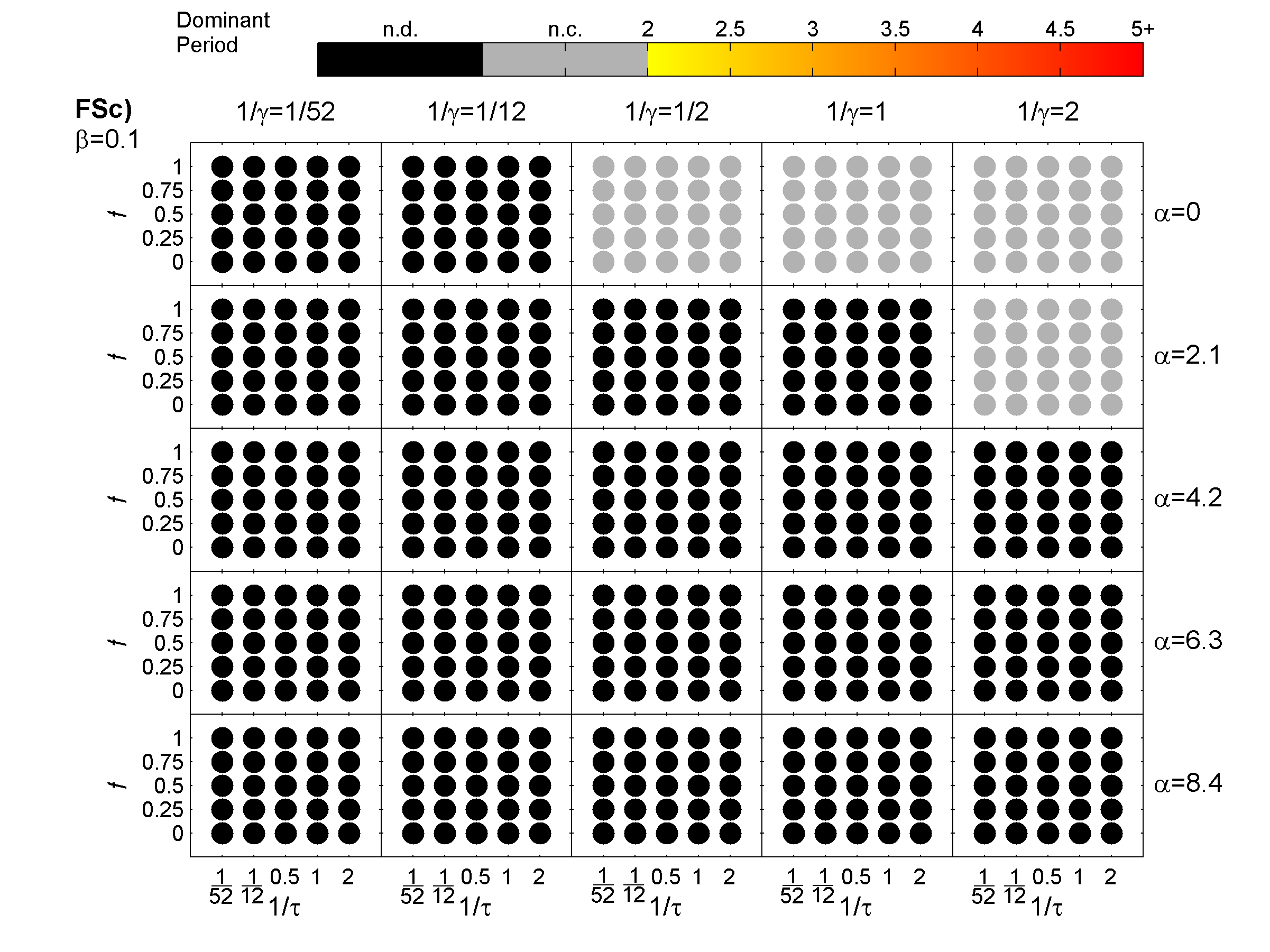

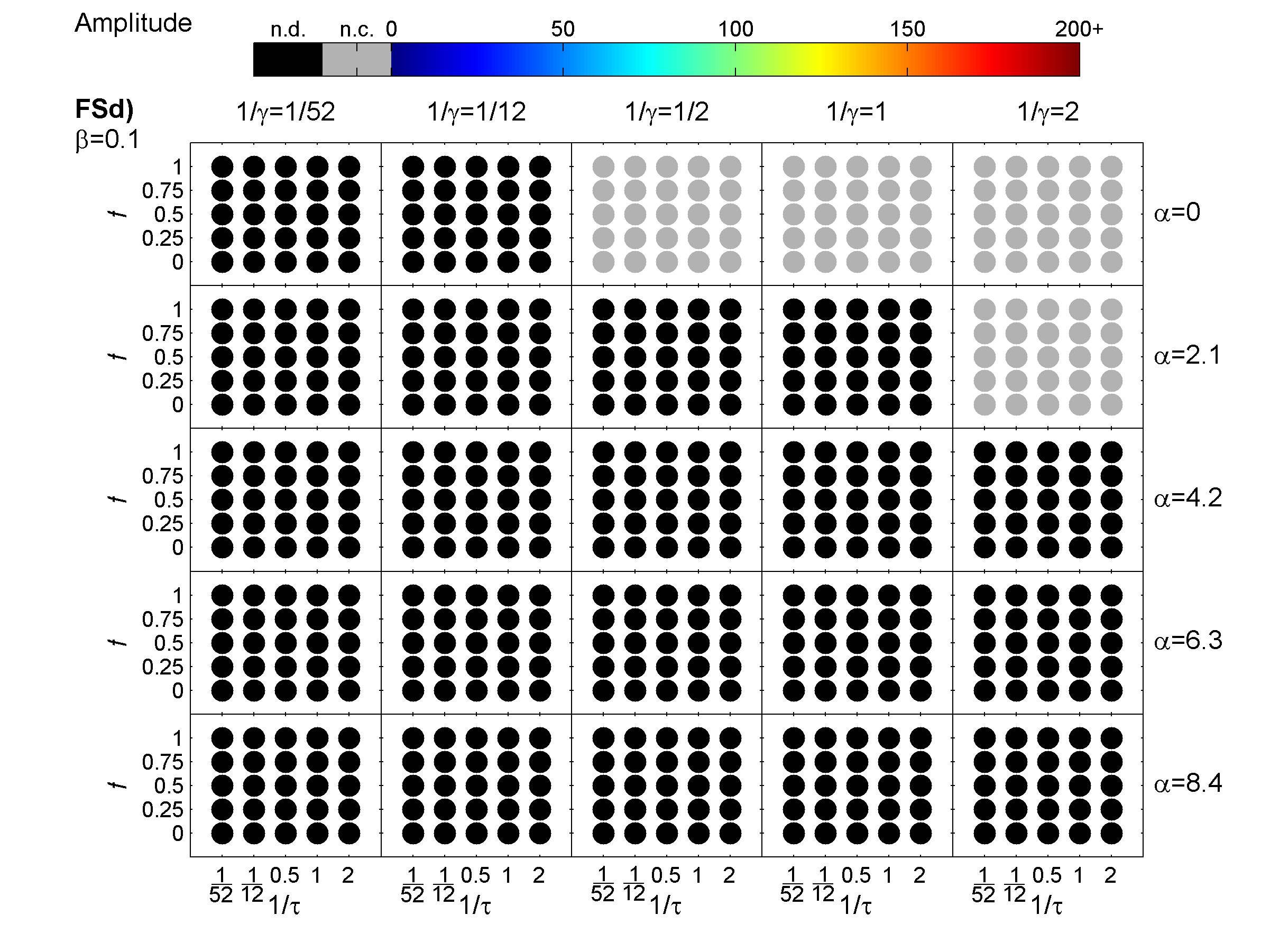

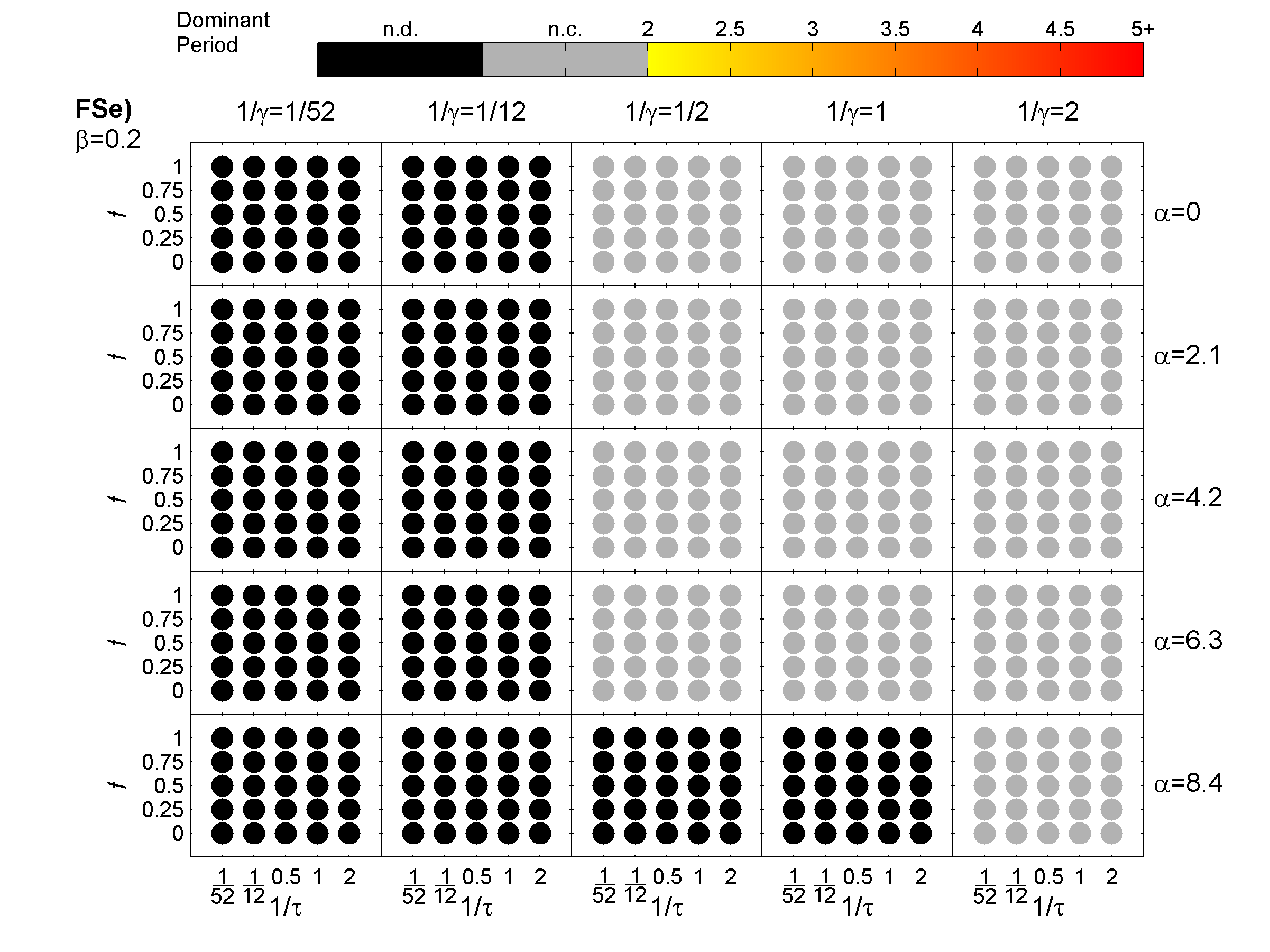

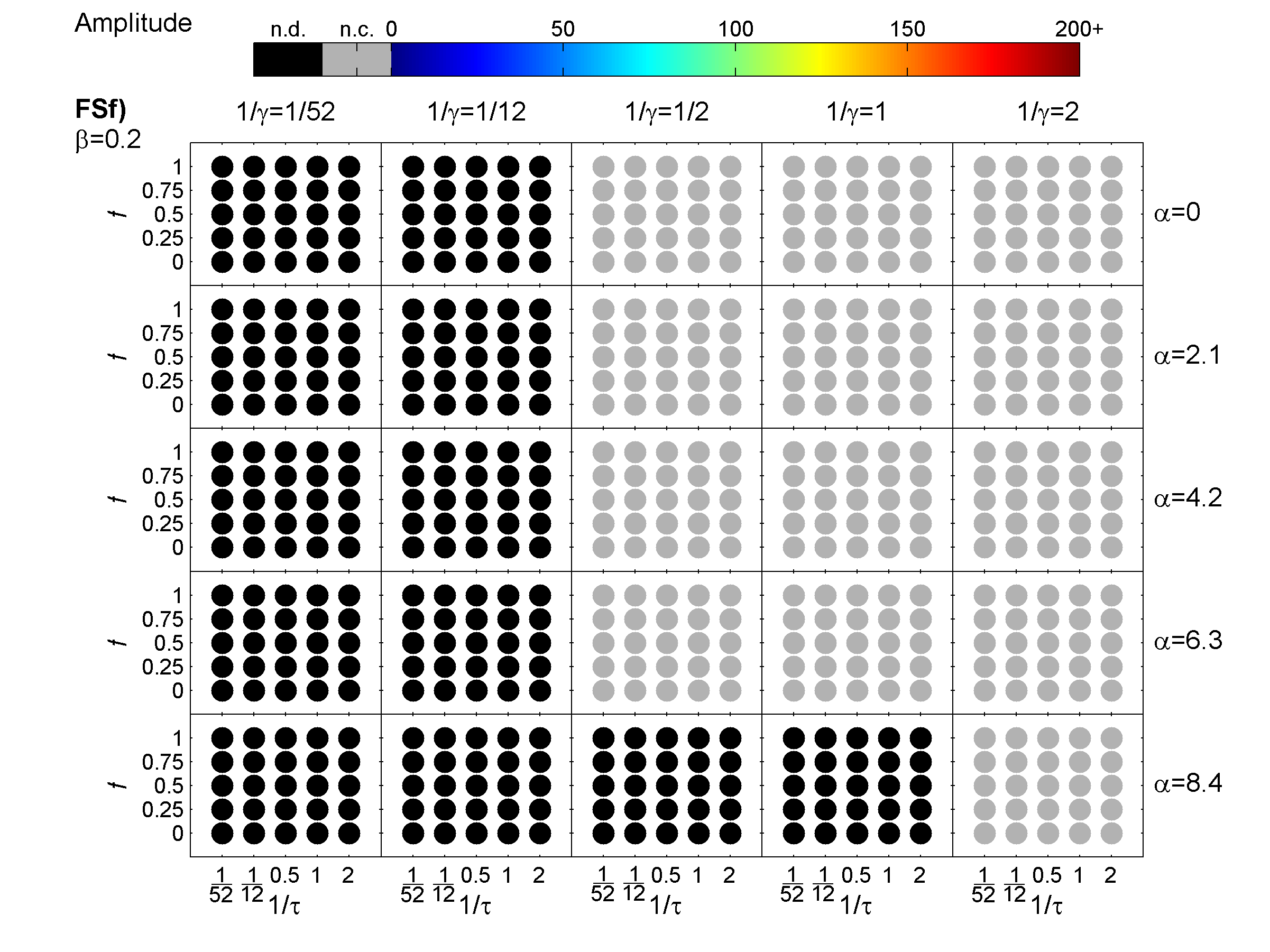

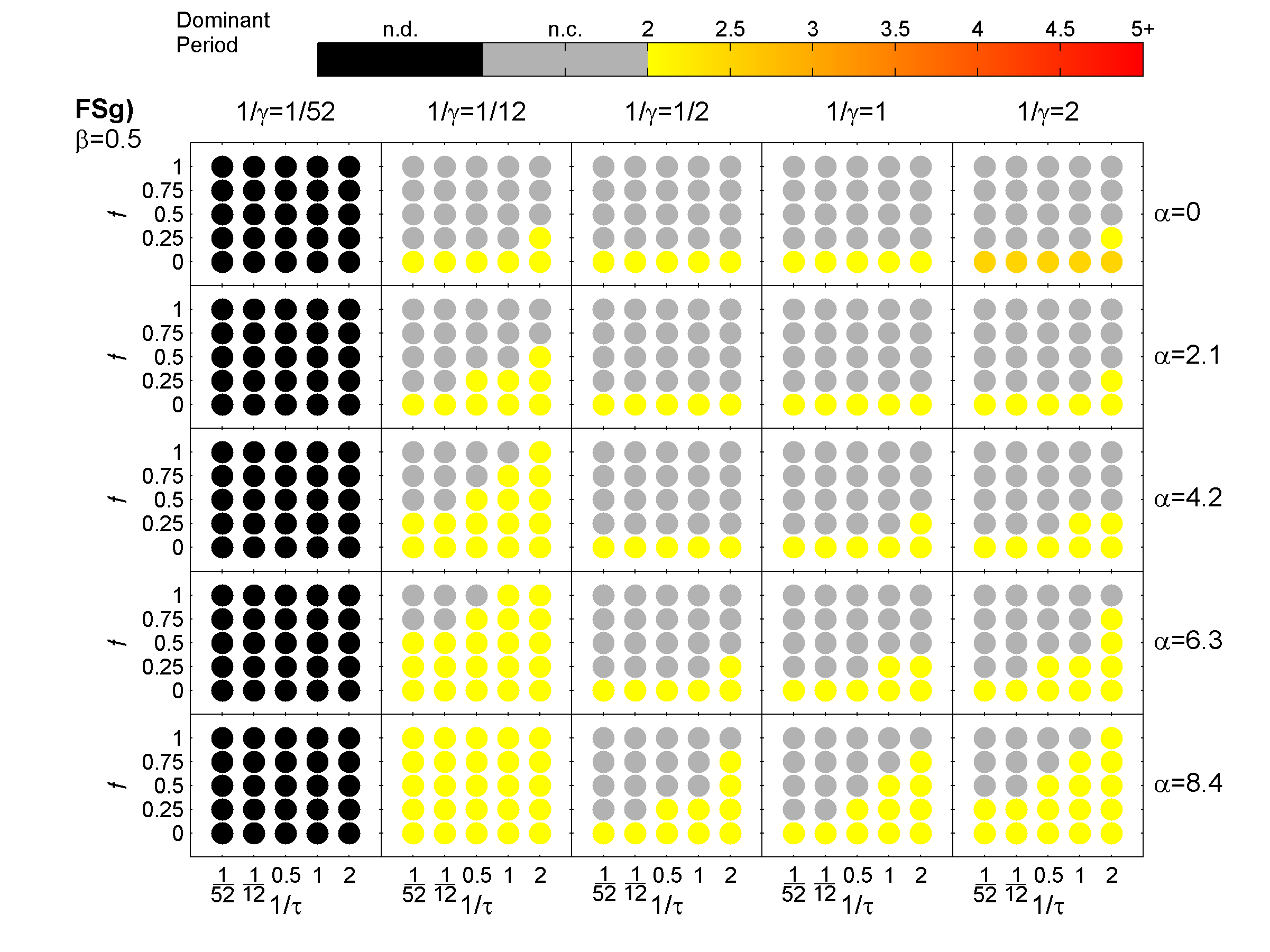

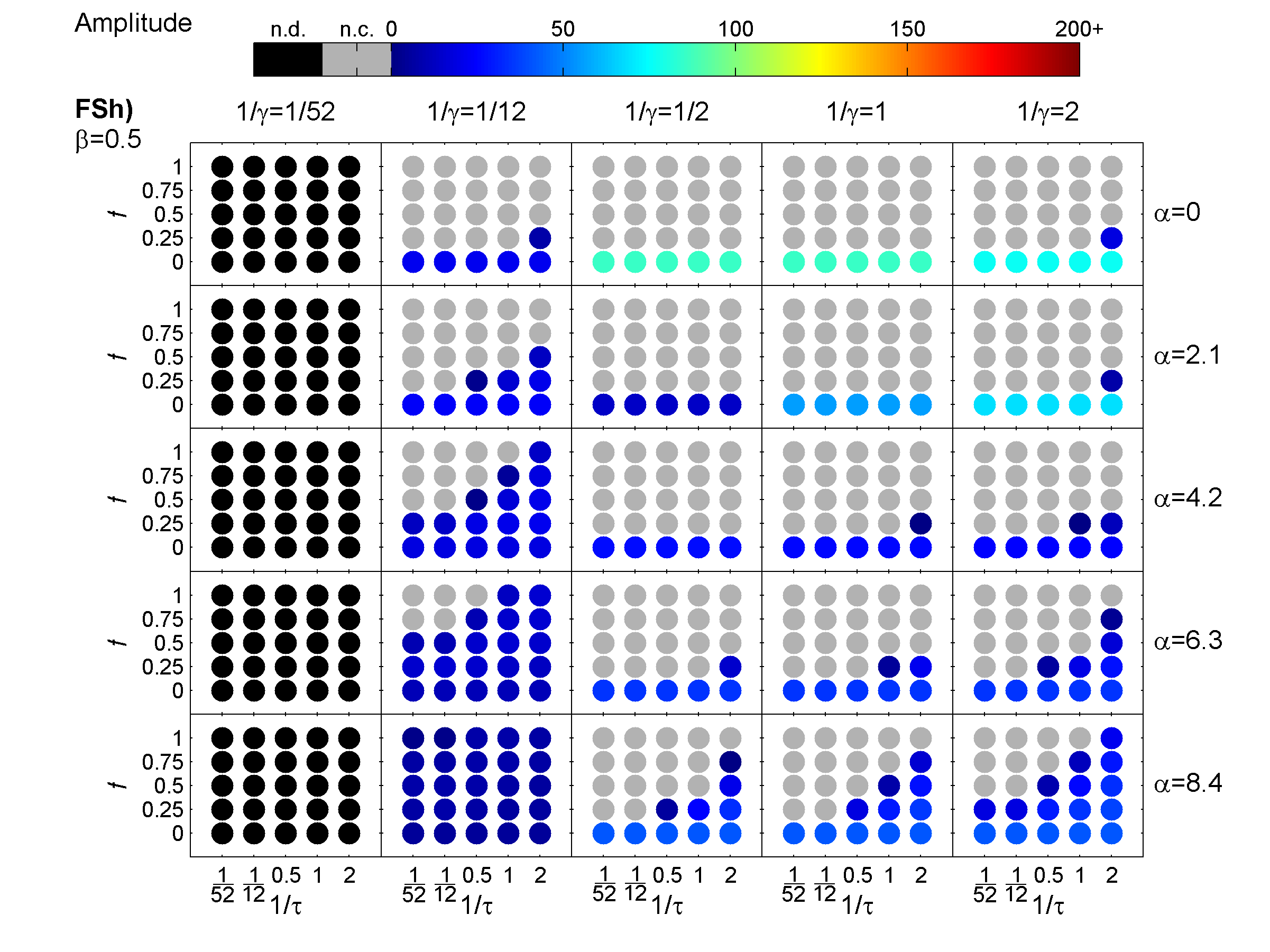

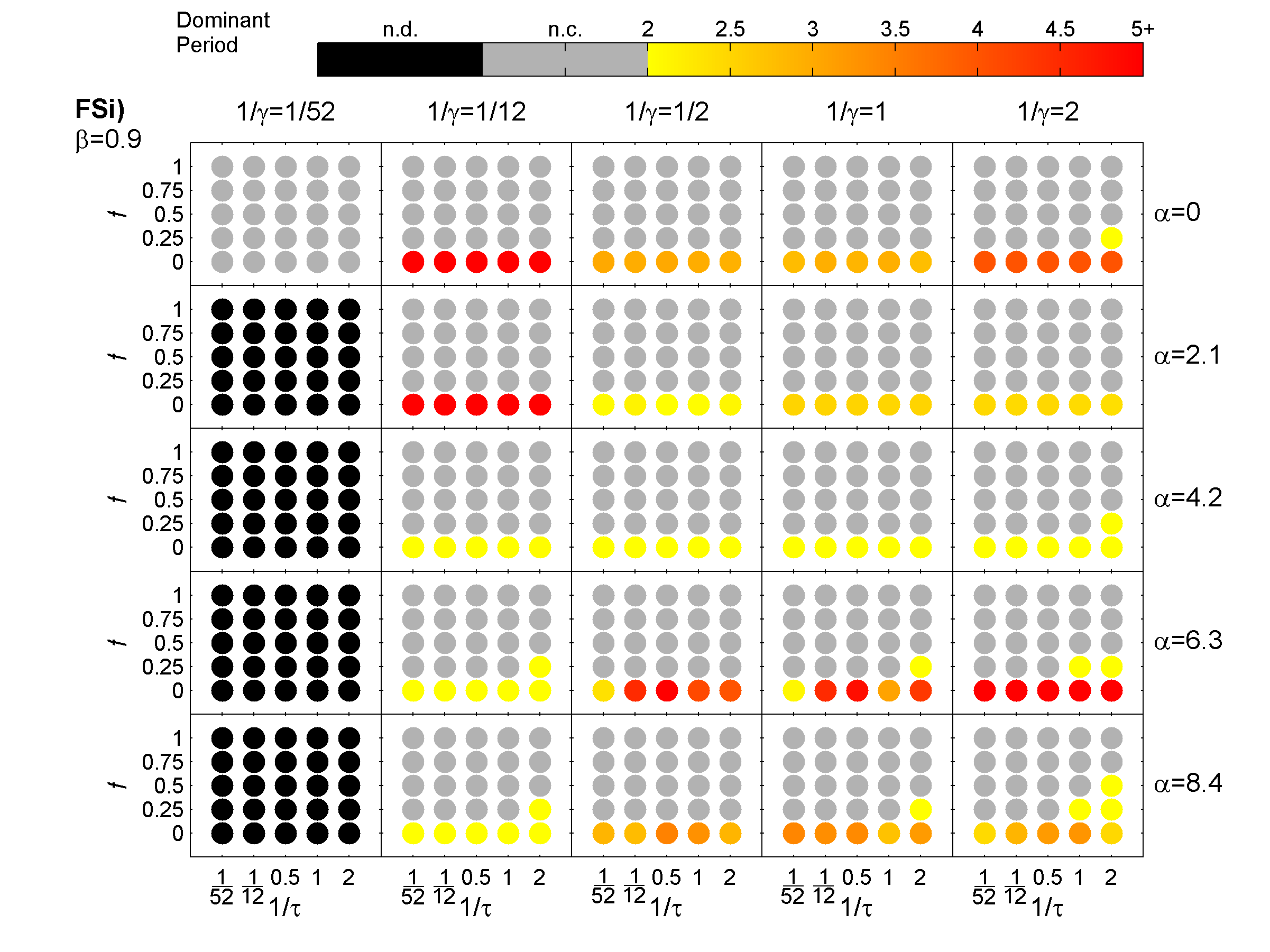

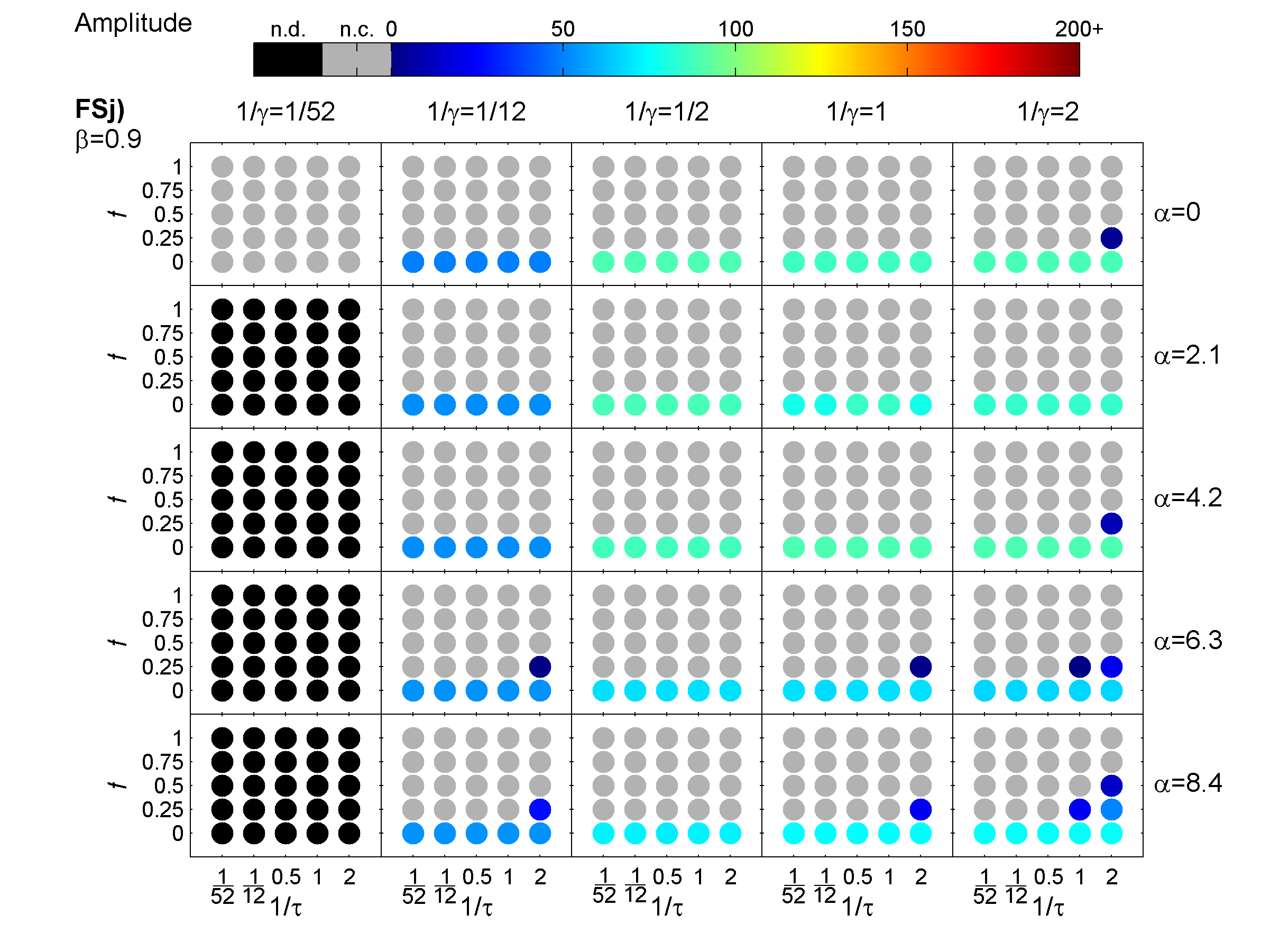
**

**Hokkaido grey-sided voles**

**
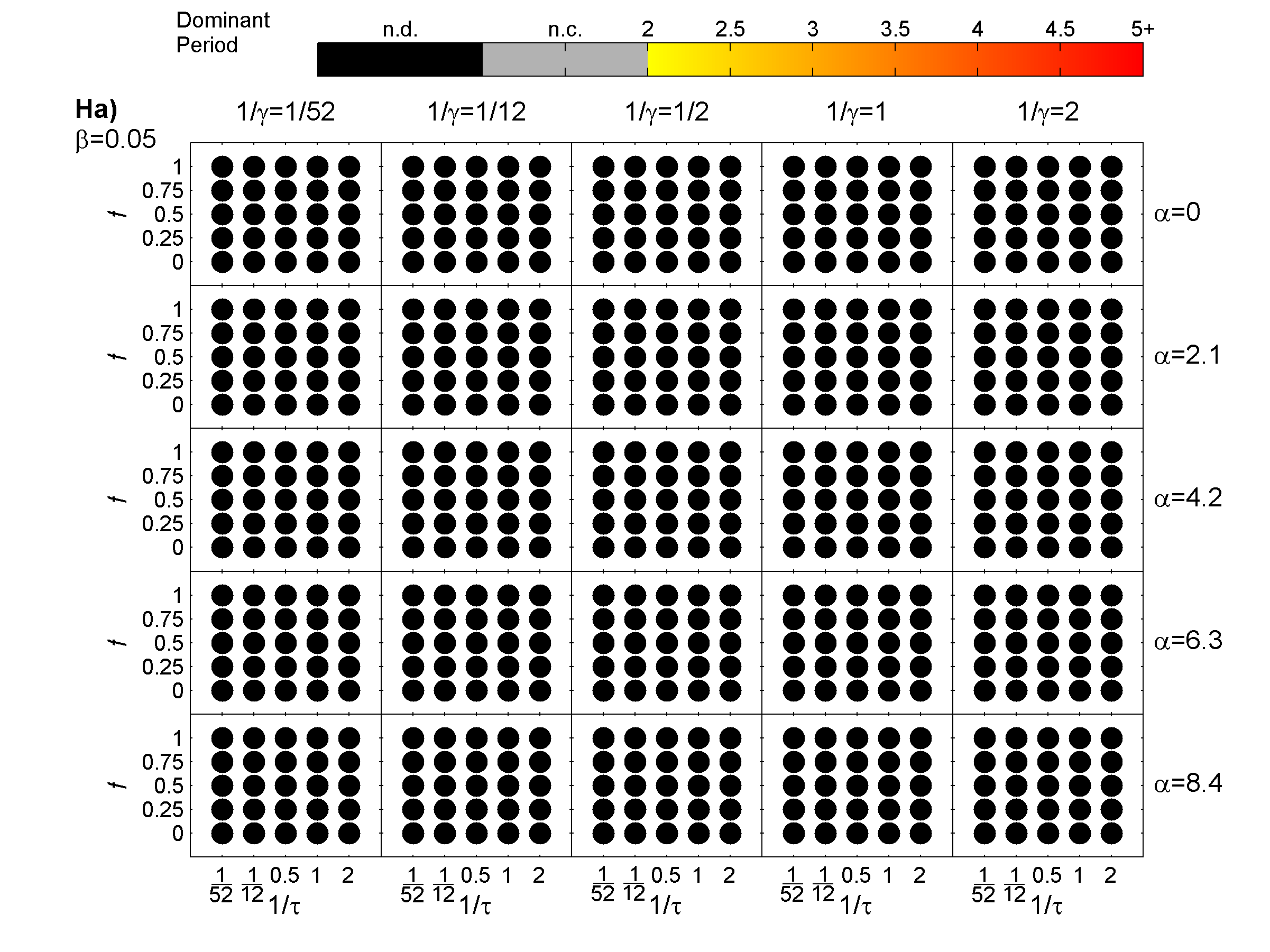

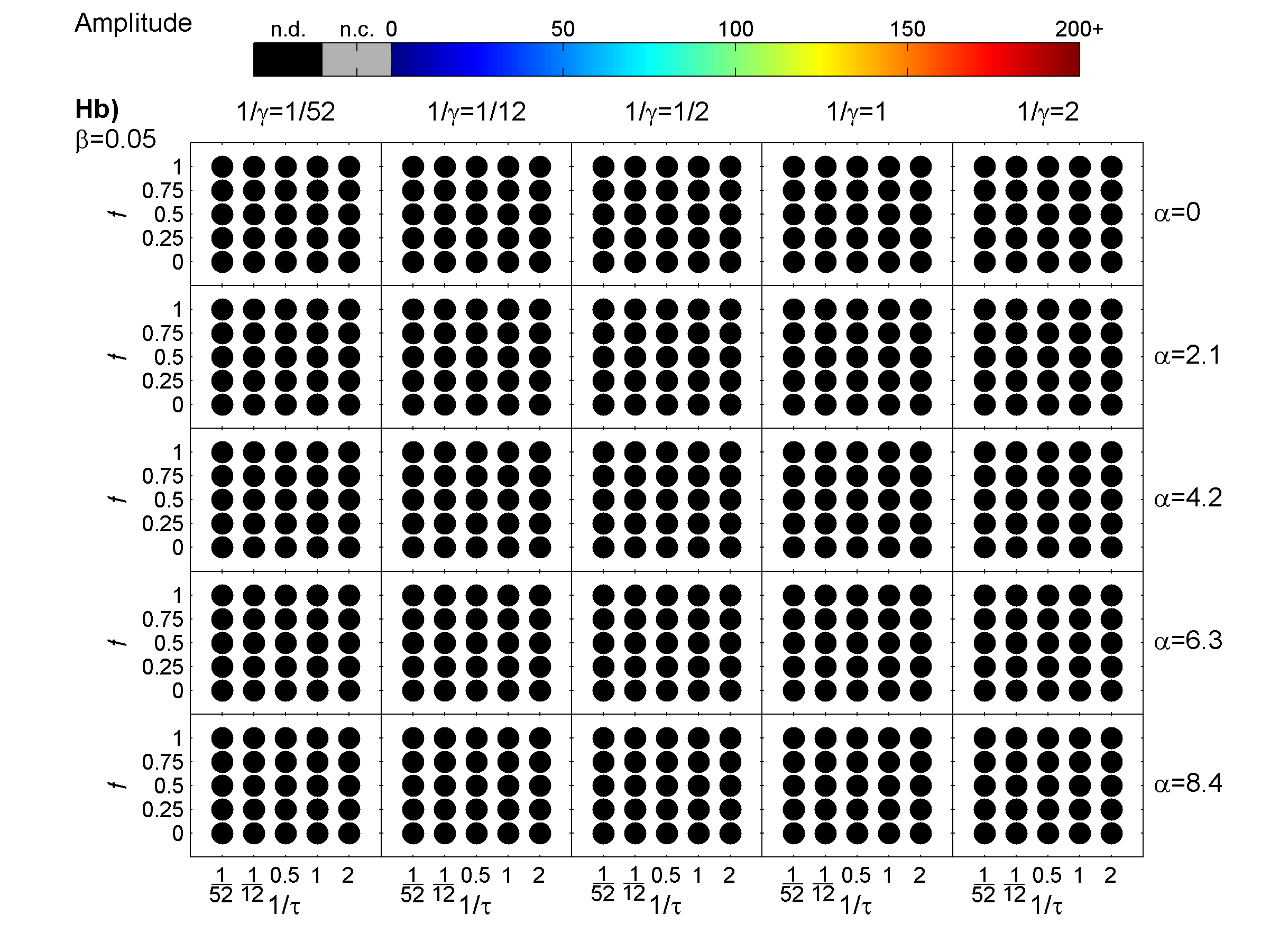

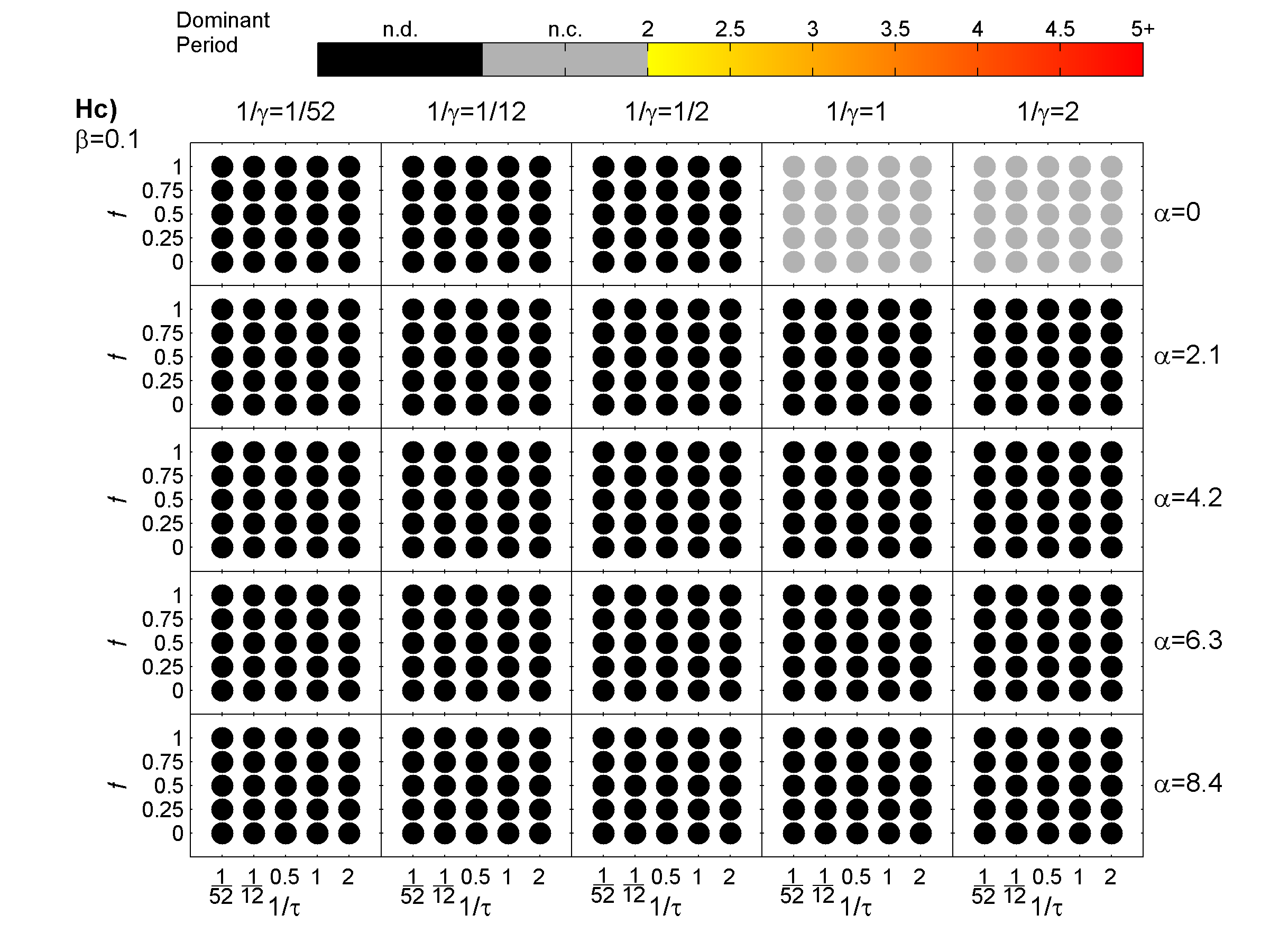

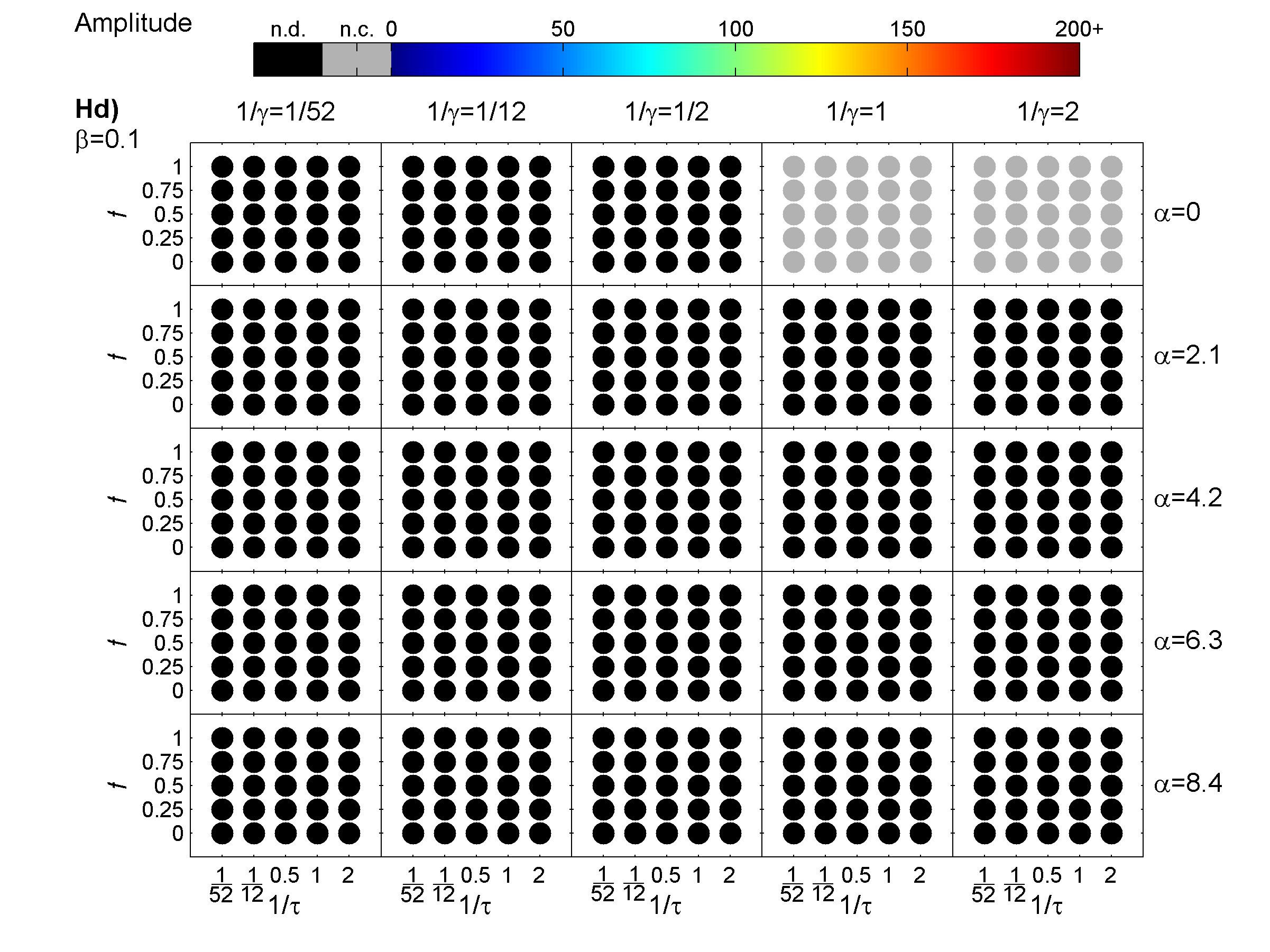

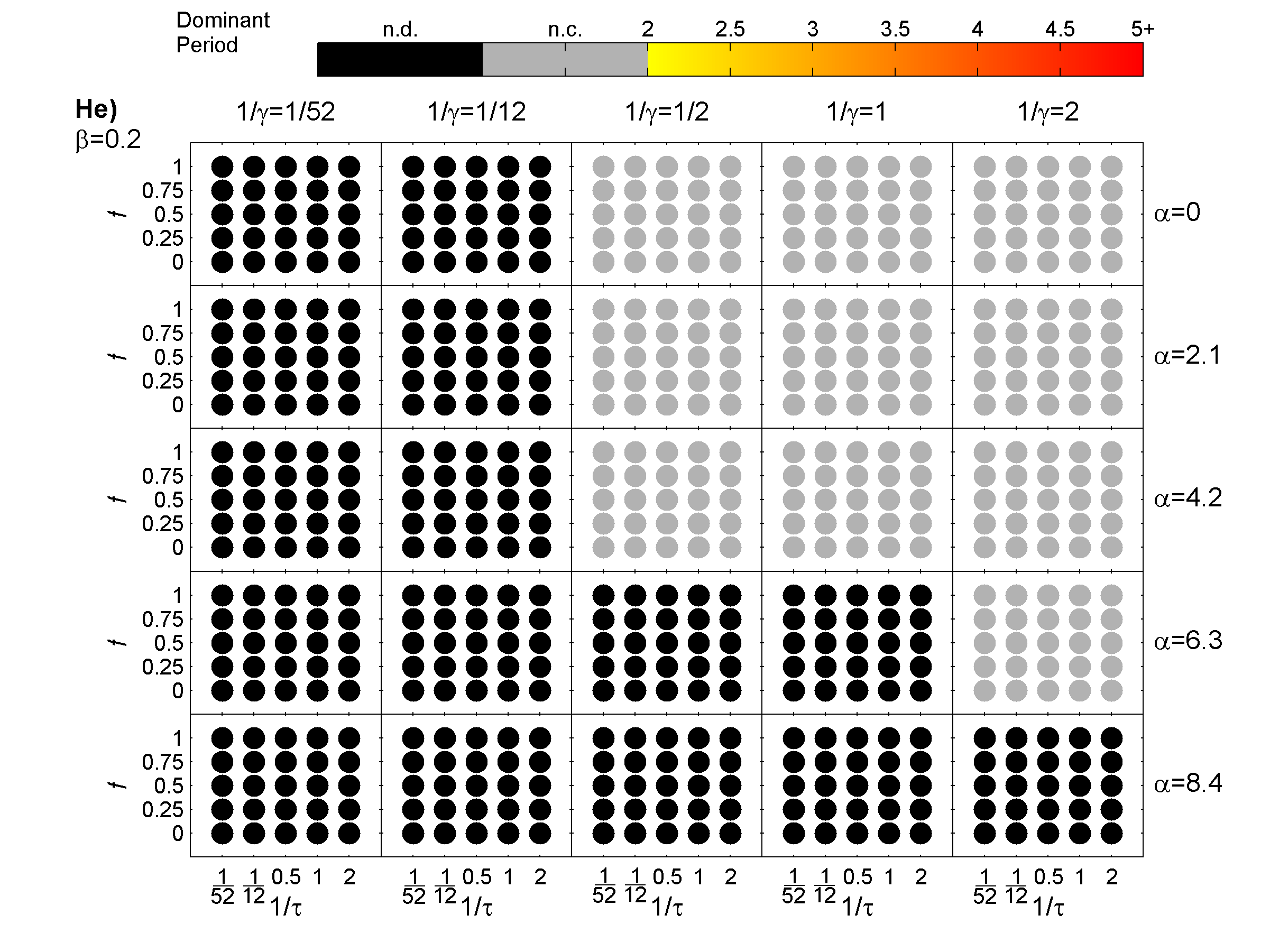

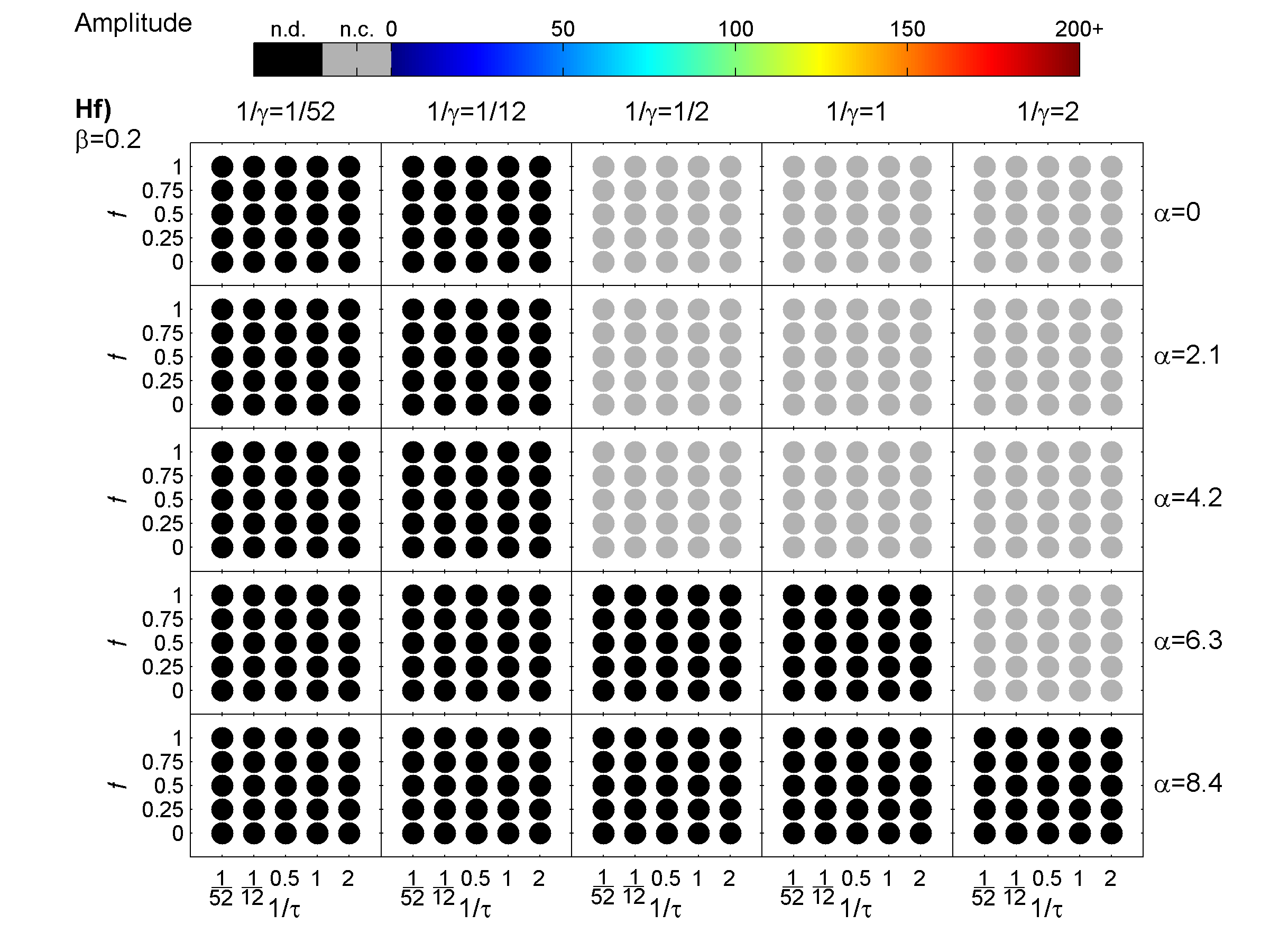

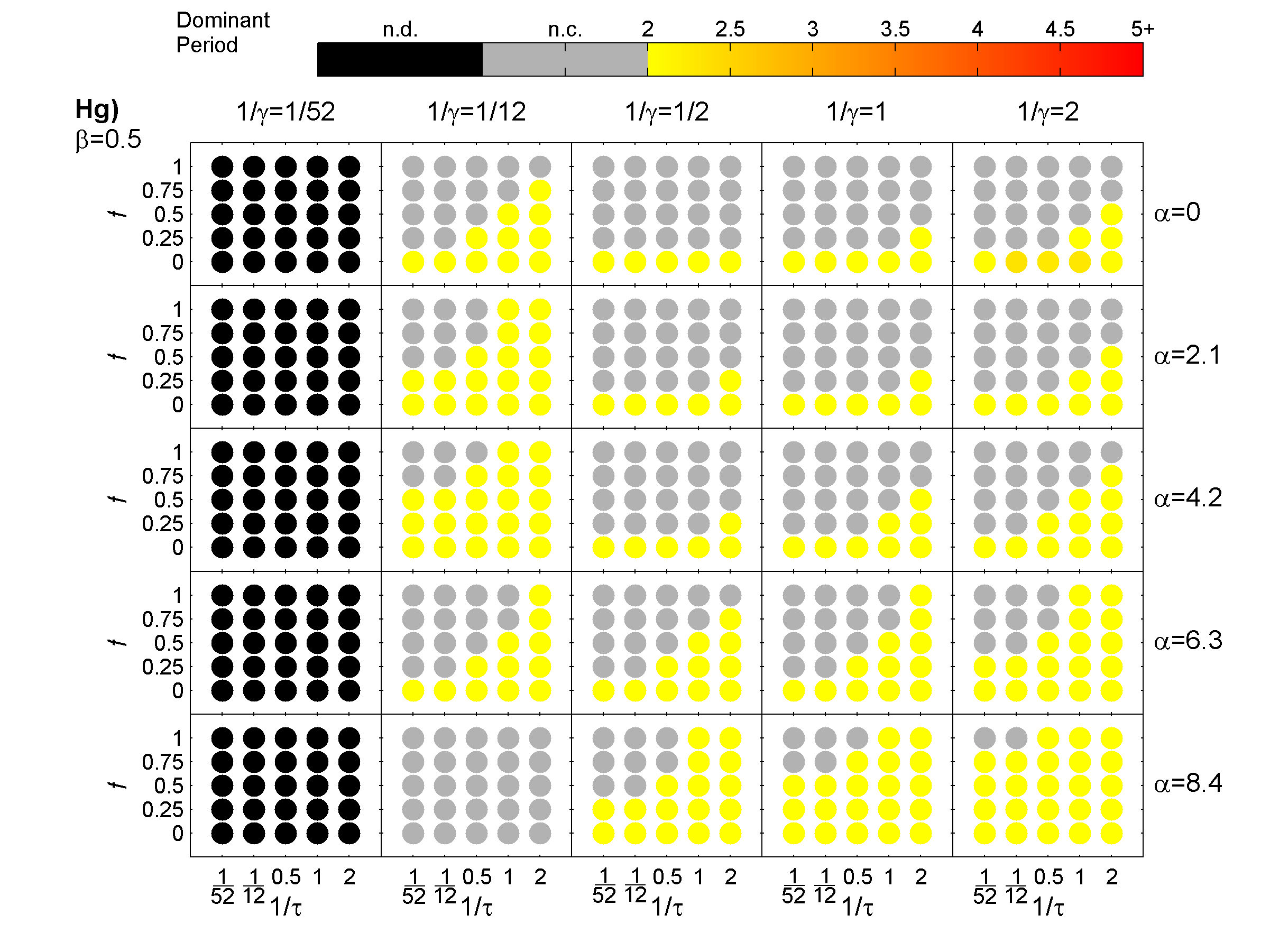

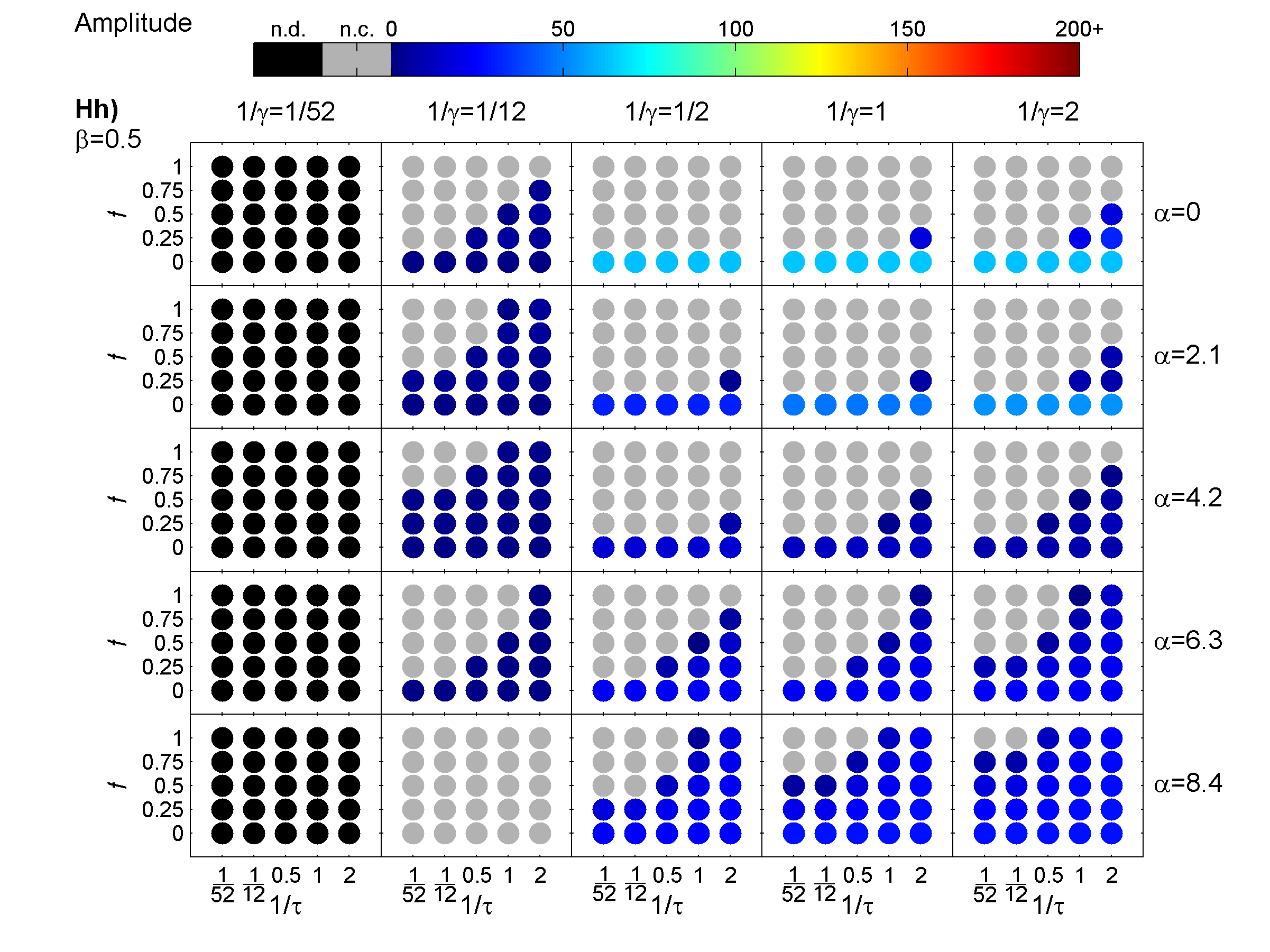

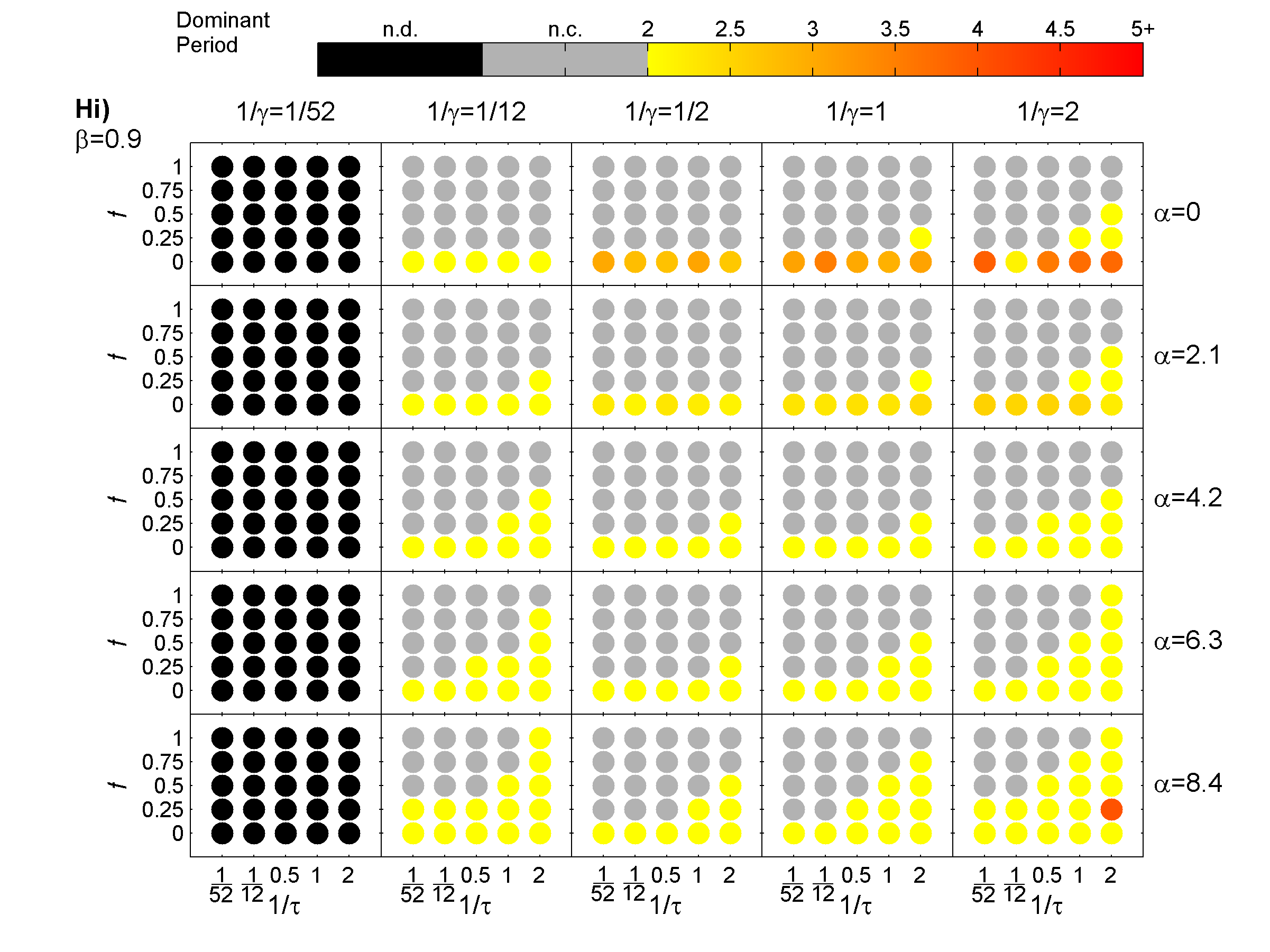

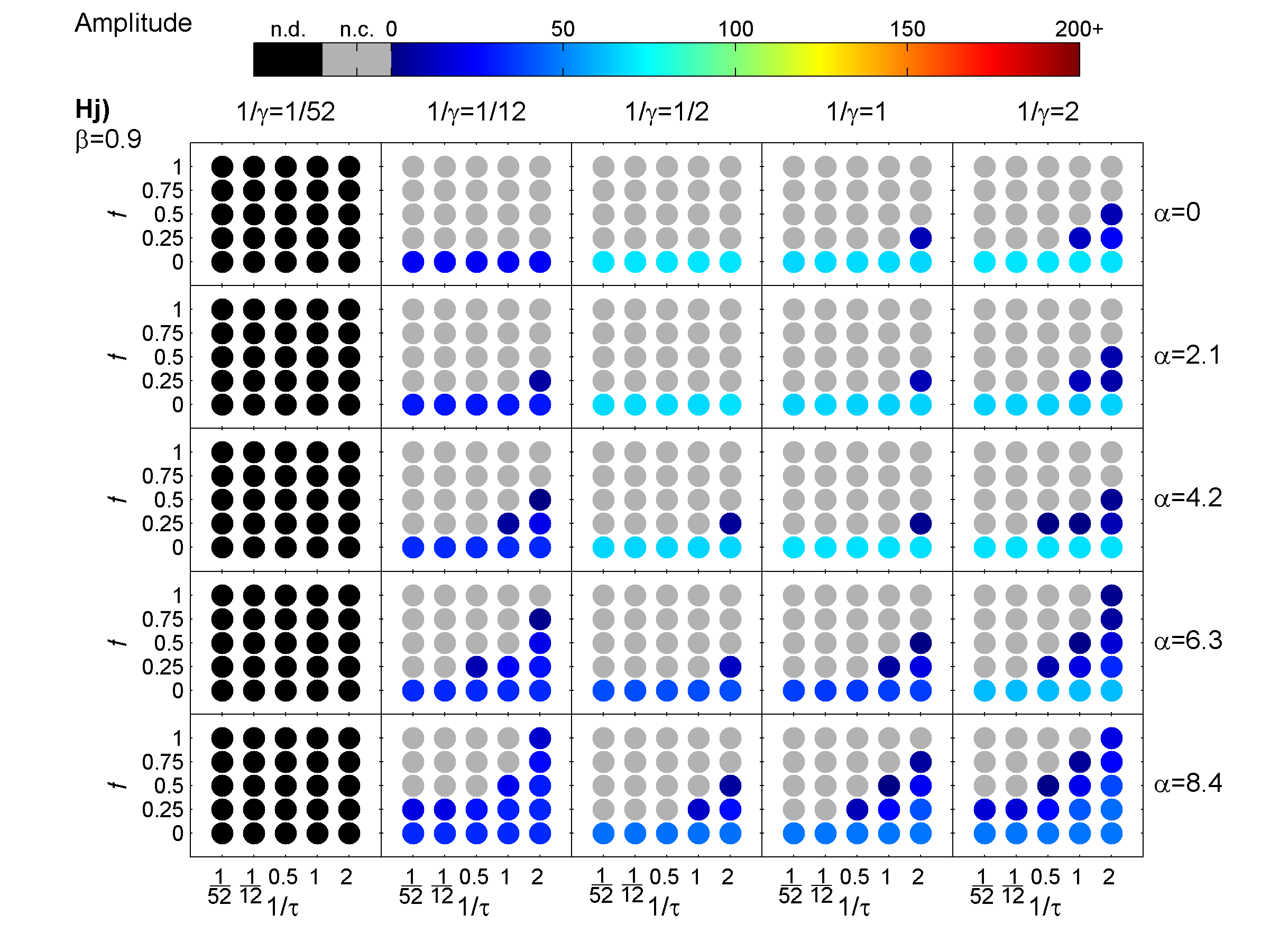
**

**References**

Begon, M., Feore, S.M., Brown, K., Chantrey, J., Jones, T., & Bennett, M. (1998) Population and transmission dynamics of cowpox in bank voles: testing fundamental assumptions. *Ecology Letters*, **1**, 82-86.

Begon, M., Hazel, S.M., Baxby, D., Bown, K., Cavanagh, R., Chantrey, J., Jones, T., & Bennett, M. (1999) Transmission dynamics of a zoonotic pathogen within and between wildlife host species. *Proceedings of the Royal Society of London Series B-Biological Sciences*, **266**, 1939-1945.

Blasdell, K.R. (2006) *Studies on lymphocytic choriomeningitis virus and cowpox virus in the natural rodent hosts.*, University of Liverpool.

Burthe, S. (2007) The effect of cowpox virus infection on the survival of wild field voles. In submitted.

Burthe, S., Telfer, S., Begon, M., Bennett, M., Smith, A., & Lambin, X. (in press) Cowpox virus infection in natural field vole Microtus agrestis populations: significant negative impacts on survival. *Journal of Animal Ecology*.

Carslake, D., Bennett, M., Bown, K., Hazel, S., Telfer, S., & Begon, M. (2005) Space-time clustering of cowpox virus infection in wild rodent populations. *Journal of Animal Ecology*, **74**, 647-655.

Cavanagh, R.D., Lambin, X., Ergon, T., Bennett, M., Graham, I.M., van Soolingen, D., & Begon, M. (2004) Disease dynamics in cyclic populations of field voles (Microtus agrestis): cowpox virus and vole tuberculosis (Mycobacterium microti). *Proceedings of the Royal Society of London Series B-Biological Sciences*, **271**, 859-867.

Ergon, T. (2003) *Fluctuating life-history traits in overwintering field voles (Microtus agrestis)*, University of Oslo.

Ergon, T., MacKinnon, J.L., Stenseth, N.C., Boonstra, R., & Lambin, X. (2001) Mechanisms for delayed density-dependent reproductive traits in field voles, Microtus agrestis: the importance of inherited environmental effects. *Oikos*, **95**, 185-197.

Hanski, I., Turchin, P., Korpimaki, E., & Henttonen, H. (1993) Population Oscillations of Boreal Rodents - Regulation by Mustelid Predators Leads to Chaos. *Nature*, **364**, 232-235.

Lambin, X., Bretagnolle, V., & Yoccoz, N.G. (2006) Vole population cycles in northern and southern Europe: Is there a need for different explanations for single pattern? *Journal of Animal Ecology*, **75**, 340-349.

Lambin, X., Petty, S.J., & MacKinnon, J.L. (2000) Cyclic dynamics in field vole populations and generalist predation. *Journal of Animal Ecology*, **69**, 106-118.

Norman, R., Begon, M., & Bowers, R.G. (1994) The Population-Dynamics of Microparasites and Vertebrate Hosts - the Importance of Immunity and Recovery. *Theoretical Population Biology*, **46**, 96-119.

Telfer, S., Bennett, M., Bown, K., Carslake, D., Cavanagh, R., Hazel, S., Jones, T., & Begon, M. (2005) Infection with cowpox virus decreases female maturation rates in wild populations of woodland rodents. *Oikos*, **109**, 317-322.

Telfer, S., Bennett, M., Bown, K., Cavanagh, R., Crespin, L., Hazel, S., Jones, T., & Begon, M. (2002) The effects of cowpox virus on survival in natural rodent populations: increases and decreases. *Journal of Animal Ecology*, **71**, 558-568.

Turchin, P. (2003) *Complex population dynamics: a theoretical/empirical synthesis*, 1st edn. Princeton University Press, Princeton and Oxford.

Turchin, P. & Hanski, I. (1997) An empirically based model for latitudinal gradient in vole population dynamics. *American Naturalist*, **149**, 842-874.

Turchin, P. & Ostfeld, R.S. (1997) Effects of density and season on the population rate of change in the meadow vole. *Oikos*, **78**, 355-361.

Yoccoz, N.G., Nakata, K., Stenseth, N.C., & Saitoh, T. (1998) The demography of Clethrionomys rufocanus: from mathematical and statistical models to further field studies. *Researches on Population Ecology*, **40**, 107-121.
